# Using a multiomics approach to unravel a septic shock specific signature in skeletal muscle

**DOI:** 10.1038/s41598-022-23544-8

**Published:** 2022-11-05

**Authors:** Baptiste Duceau, Michael Blatzer, Jean Bardon, Thibault Chaze, Quentin Giai Gianetto, Florence Castelli, François Fenaille, Lucie Duarte, Thomas Lescot, Christophe Tresallet, Bruno Riou, Mariette Matondo, Olivier Langeron, Pierre Rocheteau, Fabrice Chrétien, Adrien Bouglé

**Affiliations:** 1 Experimental Neuropathology Unit, Institut Pasteur, Paris, France; 2 Department of Anesthesiology and Critical Care Medicine, Cardiology Institute, University Hospital Pitié-Salpêtrière (AP-HP. Sorbonne Université), GRC 29, Assistance Publique, 47-83 Boulevard de L’Hôpital, 75013 Paris, France; 3 AP-HP, Department of Anesthesiology and Critical Care Medicine, Hôpital Henri Mondor, Créteil, France; 4 Institut Pasteur, Proteomics Core Facility, Mass Spectrometry for Biology Unit USR CNRS 2000, Bioinformatics and Biostatistics Hub Computational Biology Department USR CNRS 3756, Paris, France; 5 Département Médicaments Et Technologies Pour La Santé (MTS), Université Paris Saclay, CEA, INRAE, MetaboHUB, Gif-Sur-Yvette, France; 6 Department of Anesthesiology and Critical Care Medicine, Hôpital Saint-Antoine, Sorbonne Université, GRC 29, AP-HP, Paris, France; 7 Department of General and Endocrine Surgery, Hôpital La Pitié-Salpêtrière, Sorbonne Université, AP-HP, Paris, France; 8 Emergency Department, Hôpital La Pitié-Salpêtrière, Sorbonne Université, AP-HP, Paris, France; 9 Hôpital Sainte Anne, GHU Paris Psychiatrie Et Neurosciences, Paris, France

**Keywords:** Acute inflammation, Sepsis

## Abstract

Sepsis is defined as a dysregulated host response to infection leading to organs failure. Among them, sepsis induces skeletal muscle (SM) alterations that contribute to acquired-weakness in critically ill patients. Proteomics and metabolomics could unravel biological mechanisms in sepsis-related organ dysfunction. Our objective was to characterize a distinctive signature of septic shock in human SM by using an integrative multi-omics approach. Muscle biopsies were obtained as part of a multicenter non-interventional prospective study. Study population included patients in septic shock (S group, with intra-abdominal source of sepsis) and two critically ill control populations: cardiogenic shock (C group) and brain dead (BD group). The proteins and metabolites were extracted and analyzed by High-Performance Liquid Chromatography-coupled to tandem Mass Spectrometry, respectively. Fifty patients were included, 19 for the S group (53% male, 64 ± 17 years, SAPS II 45 ± 14), 12 for the C group (75% male, 63 ± 4 years, SAPS II 43 ± 15), 19 for the BD group (63% male, 58 ± 10 years, SAPS II 58 ± 9). Biopsies were performed in median 3 days [interquartile range 1–4]) after intensive care unit admission. Respectively 31 patients and 40 patients were included in the proteomics and metabolomics analyses of 2264 proteins and 259 annotated metabolites. Enrichment analysis revealed that mitochondrial pathways were significantly decreased in the S group at protein level: oxidative phosphorylation (adjusted *p* = 0.008); branched chained amino acids degradation (adjusted *p* = 0.005); citrate cycle (adjusted *p* = 0.005); ketone body metabolism (adjusted *p* = 0.003) or fatty acid degradation (adjusted *p* = 0.008). Metabolic reprogramming was also suggested (i) by the differential abundance of the peroxisome proliferator-activated receptors signaling pathway (adjusted *p* = 0.007), and (ii) by the accumulation of fatty acids like octanedioic acid dimethyl or hydroxydecanoic. Increased polyamines and depletion of mitochondrial thioredoxin or mitochondrial peroxiredoxin indicated a high level of oxidative stress in the S group. Coordinated alterations in the proteomic and metabolomic profiles reveal a septic shock signature in SM, highlighting a global impairment of mitochondria-related metabolic pathways, the depletion of antioxidant capacities, and a metabolic shift towards lipid accumulation.

ClinicalTrial registration: NCT02789995. Date of first registration 03/06/2016.

## Introduction

Sepsis is defined as a dysregulated host response to infection, resulting in acute life-threatening organ failure^1^. During septic shock, the most severe clinical presentation of sepsis, the overwhelming pro- and anti-inflammatory responses^2^ are accompanied by systemic alterations in non-immunologic pathways^3^ like vascular, autonomic nervous system, neuro-endocrine, metabolic or coagulation disorders, leading to multiple organ failure, including skeletal muscle dysfunction. In critically ill patients, muscle dysfunction is multifactorial, resulting both from structural alterations related to muscle wasting due to imbalanced protein turnover^4,5^, and from functional alterations related to mitochondrial dysfunction and bioenergetics failure^6,7^, microcirculatory disturbance^8^, muscle membranes inexcitability and ion channel dysfunction^9^.

Despite the progress over the last decades in the understanding of muscle dysfunction during sepsis, neither any drug nor targeted therapies have been found to be effective. This partly results from the difficulty and complexity of mechanistic studies in human muscle, for both ethical and practical reasons. Proteomics and metabolomics as high throughput technologies aim to identify and quantify proteins/metabolites at a large scale in biological samples. Plasma metabolomic profiles have been used for the diagnosis^10,11^ or the prognosis^12,13^ of sepsis, but fewer studies have investigated the mechanisms underlying organ dysfunction using these innovative techniques, although their integration could allow delineating more holistic effects of sepsis in skeletal muscle and providing a better understanding of sepsis-related muscle dysfunction.

Our goal was to unravel a specific proteomic/metabolomic septic shock signature in skeletal muscle biopsies (S group). Two control populations were included, a cardiogenic shock group (C group) and a brain dead group (BD group), both suffering a variety of non-specific muscle insults, and subjected to an inflammatory process resembling to sepsis^14–16^, in order to control partially for the inflammatory pathways and to focus on the non-immunologic pathways that could be associated with or responsible for sepsis-related muscle dysfunction. Multi-omic data-integration was conducted to compare septic shock patients to controls in order to identify the multi-omic signature of septic shock in skeletal muscle.

## Methods

### Study design and populations

This non-randomized prospective observational investigation enrolled adult patients (≥ 18 years of age) from June 2016 to November 2018, at three intensive care units (ICUs) from university hospital in Paris, France. Three populations of patients were prospectively included after written informed consent by the patient or his/her relatives: patients with septic shock (S group), patients with cardiogenic shock (C group), and brain dead patients (BD group). For the S group, the inclusion criterion was an intra-abdominal septic shock requiring emergent surgery, septic shock being identified by a vasopressor requirement to maintain a mean arterial blood pressure ≥ 65 mmHg and/or serum lactate level > 2 mmol/L^1^. For the C population, the inclusion criterion was a refractory cardiogenic shock requiring extra corporeal life support. For the BD population, the inclusion criterion was a brain dead patient scheduled for multi-organ retrieval. Extended inclusion criteria for the three groups are available in the digital supplemental material methods. The exclusion criteria were the same for all three populations: patients under 18 years of age, pregnancy and or preexisting neuromuscular diseases. An additional exclusion criterion for the C and BD groups was septic shock. For the BD population, the non-objection from relatives for biological sample donation was mandatory.

The results described here are prespecified ancillary study of the Dysfunction of Human muscle stem cells in sepsis study (ClinicalTrial.gov NCT02789995, date of first registration 03/06/2016), this study having inconclusive results. The research protocol was approved by an Institutional Review Board (Comité de Protection des Personnes “Ile de France 5”, #15,051), and the study was declared to the French National Commission on Information Technology and Liberties (DR-2016–271), and all experiments were performed in accordance with relevant guidelines and regulations. The study size was computed for the original study, this prespecified ancillary study included patients with sufficient biological material available. Our report complies with the STROBE statement for transparent reporting of an observational study^17^.

### Muscle samples

The 2 cm^3^ surgical muscle biopsy was performed under general anesthesia (except in the BD group). It aimed to be as minimally invasive as possible and interested skeletal muscle exposed by the surgical incision. The *rectus abdominis* muscle was harvested during laparotomy surgery in the S group. The *vastus lateralis* muscle was harvested during the surgical approach of the iliac vessels during extracorporeal life support implantation in the C group. The *psoas major* was harvested during multi-organ retrieval procedure in the BD group. The muscle samples were stored in a minimal storage medium (F12 Nutri Mix [Thermo Fisher Scientific, Waltham, Massachusetts, USA]; 1% HEPES buffer) at 4 °C and processed within 12 h. The non-muscular tissues (adipose tissue, tendons) were carefully removed, to ensure that subsequent analyses were specific to muscle tissue. The muscle was subsampled for the different analyses, these subsamples were snap-frozen in liquid nitrogen, then preserved at − 80 °C until further use.

### Proteins and metabolites extraction, identification and quantification

Protein and metabolite extractions were performed in parallel, but simultaneously for all the samples to avoid any batch effect. After mechanical lysis of the muscle samples, extracted proteins and metabolites (see supplemental material methods) were subjected to high-resolution liquid chromatography coupled to high resolution mass spectrometry (LC-HRMS, using two distinct platforms for metabolomics, see supplemental material methods). Raw data extraction, peak identification, quality check and processing were carried out using MaxQuant freeware (v. 1.5.3.8) for proteomics, and Xcalibur (Thermo Fisher Scientific, version 2.1) coupled to XCMS software package (W4M platform^18^) for metabolomics analyses. Proteins were identified using the UniprotKB human database^19^. The metabolites annotation was first accomplished using an in-house spectral database according to accurately measured masses and chromatographic retention times (see eTable 1). This chemical database includes ~ 1000 pure authentic standards mainly of human origin^20^. The annotation of relevant metabolites was further validated by performing additional tandem mass spectrometry experiments (MS/MS, eTable 2). A label-free quantification workflow was used for both proteomic and metabolomic analyses. Peptides and metabolites were quantified by integrating their corresponding chromatographic peaks.

### Bioinformatic analyses

Quality checks were conducted according to the state of the art, causing the exclusion of several samples (Figure S1 A–F). The proteomic and metabolomic datasets were treated similarly: after log2transformation, a median centering normalization was performed to remove any source of systematic variability that may have biased the analyte quantifications (overall analyte concentration, pipetting variation, batch effect; Figure S2 and S3). High-throughput data usually generate missing values, even if samples with more than 70% of missing values were excluded (see supplemental material methods). Several imputation methods were performed to handle missing values in the proteomics dataset, under different hypotheses (see supplemental material for details, Figures S4 and S5). The main enrichment analysis was conducted using the probabilistic minimum imputation method^21^, adapted for the missing not-at-random (MNAR) missing values hypothesis (Figures S4A and S5A). Two other imputation algorithms adapted for missing completely-at-random (MCAR) missing values hypothesis were used to refine the results of enrichment analyses: imputations with the maximum likelihood estimation^22^ (Figures S4B and S5B) and with the structured least squares algorithm^23^ (Figure S4C and S5C). Finally, a complete case analysis without any imputation was performed (removal of the proteins with at least one missing value). The proportion of missing values being low in metabolomics (less than 3%), only the probabilistic minimum imputation method was used.

### Statistical and functional analyses

Continuous variables were reported as medians with interquartile range [IQR] or means with standard deviation (SD) depending on their distribution. Categorical variables were reported as count (percentage). To check for outliers and sample clustering, a principal component analysis (PCA) was performed on imputed data matrices of proteomics and metabolomics. First, the differential abundance of proteins and metabolites in the three groups was assessed using an analysis of variance test, and the p-values were corrected for multiple imputation by the adaptive Benjamini–Hochberg procedure. Differentially abundant metabolites were represented in heat maps. Then, an enrichment analysis was performed on the proteomic dataset, based on the Kyoto Encyclopedia of Genes and Genomes (KEGG) pathways database^24,25^. The aim of enrichment analysis is to assess which relevant functional or mechanistic biological processes are significantly enriched between two or more experimental groups. Two different enrichment analysis algorithms were used: the covariance analysis (Ancova) global test^26,27^ which allows the comparison of three groups of patients, and the generally applicable gene-set enrichment (GAGE)^28^ algorithm which allows pairwise comparisons. A competitive null hypothesis was used for enrichment analysis (H_0_) “The proteins in the pathway are at most as often differentially expressed as the proteins in the entire dataset”. Following proteomics enrichment analyses, the results of the metabolomics were manually integrated to identify points of convergence between proteomics and metabolomics, that could be interpreted as corresponding relevant biological modifications.

The statistical analyses were performed using R Software (version 3.6.0 for Mac OS, The R foundation for statistical computing). The “imp4p” package^29^ was used for missing values imputation. The “gage”^28^ and the “GlobalAncova”^27^ packages were used for enrichment analyses and the “pathview” package^30^ was used to generate the KEGG pathway graphs. Heat maps were generated using the “ComplexHeatmap”^31^ package, with scaling of relative intensities along the samples for each analyte. Except otherwise stated, a *p* value < 0.05 was considered for significance and all comparisons were two-tailed. The Benjamini–Hochberg procedure^32^ was performed when required to account for multiple comparisons, significance being defined by a false discovery rate (FDR) adjusted *p* values < 0.01. Raw *p* values are presented unless stated differently.

### Ethics approval and consent to participate

Patients were prospectively included after written informed consent by the patient or his/her relatives. The research protocol was approved by an Institutional Review Board, Comité de Protection des Personnes “Ile de France 5”, under the approval number #15051, and the study was declared to the French National Commission on Information Technology and Liberties (DR-2016-271).

## Results

### Patients

**Figure 1:**
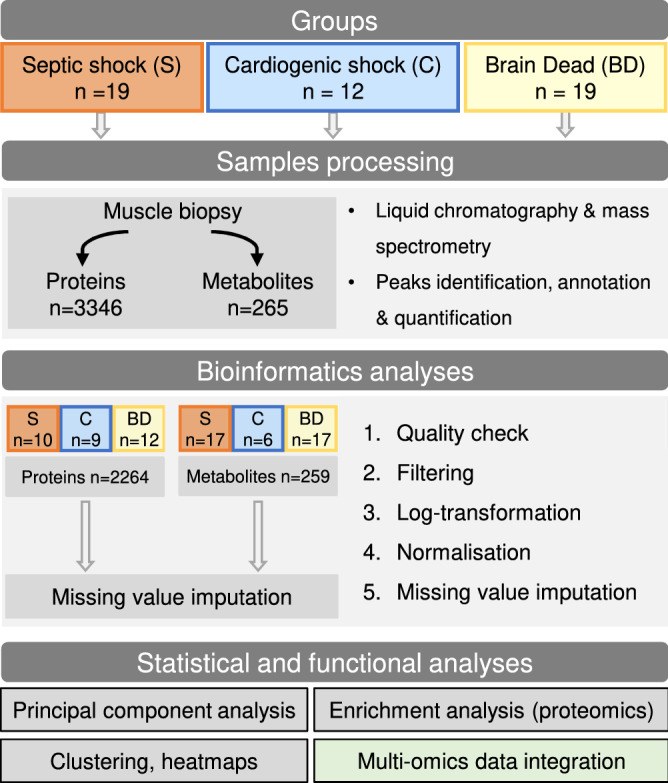
Study design and general overview.

Fifty patients which all required mechanical ventilation were included in the study (S group n = 19, C group n = 12, BD group n = 19, see Fig. 1 and Table 1), allowing the identification of 3346 unique proteins and the annotation of 265 metabolites (Fig. 1). After the filtering step, 2264 proteins in 31 patients (S n = 10, C n = 9, BD n = 12) and 259 metabolites in 40 patients (S n = 17, C n = 6, BD n = 17) remained for further analysis (metabolites are detailed in eTable 1 and eTable 2). Regarding demographics, no differences were found between the groups (Table 1). The SAPS2 score was significantly higher in the BD group, related to the neurologic failure. The vasopressors used differed significantly in type and dose due to good medical practices regarding disease management in each group. The muscle biopsies were performed early, in median 3 days [interquartile range 2, 4] after ICU admission, without significant difference between the three groups. Although biopsies were harvested from patients of different age, different sex and also different muscles, the proportions of myosin heavy chain, troponin and tropomyosin isoforms did neither show any difference in muscle composition (eTable 3), nor did non-muscle tissue-specific proteins reveal any significant contamination like Schwann cells, adipocytes, connective tissue cells, endothelial cells, immune cells or serum (eTable 4). Pro- and anti-inflammatory mediators were not detected, as well as interleukins, TNF-alpha or NK-κB in the muscle biopsies. Nevertheless, some chemokines and prostaglandins were detected, however without any differences between the groups (chemokines CXCR2, CCL18, CXCL12 and prostaglandins PG A1/B1/F1 and PGF1-alpha).

**Table 1:** Basal characteristics of the three patient groups.

Characteristics	Proteomics (n = 31)				Metabolomics (n = 40)			
	Septic shock (n = 12)	Cardiogenic shock (n = 9)	Brain dead (n = 10)	*p*	Septic shock (n = 17)	Cardiogenic shock (n = 6)	Brain dead (n = 17)	*p*
Sex, male	6 (50%)	5 (89%)	5 (50%)	0.13	9 (53%)	4 (67%)	11 (65%)	0.73
Age, years	64 (15)	64 (15)	59 (11)	0.52	65 (18)	65 (5)	57 (10)	0.22
Body weight, kg	77 (19)	79 (134	66 (10)	0.20	74 (18)	76 (18)	68 (14)	0.50
SAPS II	42 (12)	43 (17)	56 (11)	0.044	45 (15)	37 (6)	57 (10)	0.001
Antibiotic, yes	12 (100%)	3 (33%)	2 (20%)	< 0.001	17 (100%)	1 (17%)	4 (24%)	< 0.001
Vasopressor use*								
Norepinephrine, n (%)	12 (100%)	4 (44%)	9 (90%)	0.030	17 (100%)	2 (33%)	14 (82%)	0.005
Dose, µg kg^−1^ min^−1^	0.6 [0.4–1.0]	0.2 [0.1–0.3]	0.2 [0.1–0.3]	0.001	0.6 [0.5–0.9]	0.4 [0.3–0.5]	0.1 [0.1–0.3]	0.002
Epinephrine, n (%)	0	2 (22%)	0	0.023	0	2 (33%)	0	< 0.001
Dose, µg kg^−1^ min^-1^	NA	0.8 [0.6,1.0]	NA	–	NA	0.9 [0.8, 1.1]	NA	–
Dobutamine, n (%)	0	5 (56%)	0	< 0.001	0	4 (67%)	0	< 0.001
Dose, µg kg^−1^ min^−1^	NA	12 [11–13]	NA	–	NA	11 [11–12]	NA	–
ICU admission to biopsy, days	3 [1–8]	2 [2–4]	3 [2–4]	0.50	2 [1–4]	2 [1–3]	3 [2–4]	0.20
Days ventilated	3 [3–4]	3 [2–36]	3 [2–4]	0.79	3 [2–4]	4 [3–15]	3 [2–4]	0.46
ICU length of stay, days	10 [7–23]	31 [8–45]	3 [2–4]	0.002	10 [7–24]	21 [6–35]	3 [2–4]	< 0.001
In-hospital death	2 (16.7%)	5 (55.6%)	10 (100%)	–	5 (33.3%)	3 (50%)	17 (100%)	–

The results are presented in count (%), mean (standard deviation) or median [interquartile range]. The doses of vasopressors are the highest recorded on the day of the muscle biopsy.

* The patients in the C group may have receive vasopressors combinations.

SAPS II, Simplified acute physiology score II. ICU, Intensive care unit. NA, Non applicable.

### Omics profiling of skeletal muscle in ICU patients

**Figure 2:**
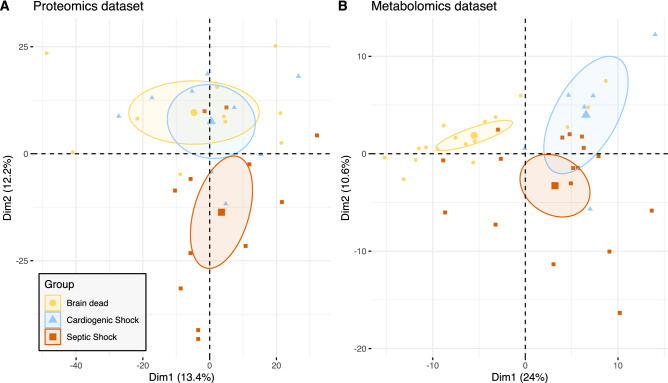
Principal component analyses of proteomic and metabolomic datasets. The two first dimensions are shown, defining the subspace maximizing the variance of the dataset. Every point represents an individual muscle proteome (**A**) or metabolome (**B**). The individuals are color-coded according to their group. Points that are close together tend to have similar proteome/metabolome. Large points and ellipses represent respectively the barycenter of each group and its 95% confidence interval. Dim indicates Dimension. Some samples were discarded due to quality checks. N = 31 patients analyzed for the proteomics (Septic shock n = 12, cardiogenic shock n = 9, brain dead n = 10) and N = 40 for the metabolomics (Septic shock n = 17, cardiogenic shock n = 6, brain dead n = 17).

Dimensionality reduction using principal PCA in muscle proteome and metabolome partially discriminated patients from the three groups (Fig. 2). The first 20 proteins that accounted for this difference (second principal component) were all mitochondrial proteins and ten (n = 10/20) were involved in the oxidative phosphorylation (eTable 5). Other proteins comprised enzymes of mitochondrial fatty acid degradation, pyruvate carrier2, mitochondrial apoptosis inducing factor1, Nipsnap homolog2 and dihydrolipoyl dehydrogenase. Similarly, the first 20 metabolites that help to distinguish the groups of patients based on the PCA are presented in the eTable 6. The PCA analyses stratified on confounding factors such as the sex, the age or the patient’s severity (SAPS II) did not reveal any noticeable effect of these covariates (Figure S6). One hundred and fifty-five proteins and 39 metabolites were differentially abundant between the three groups (eTable 7 and eTable 8). Histidine was significantly down regulated in the S group compared to the controls. Histidine metabolism is interconnected with nucleotide formation as the intermediate aminoimidazolecarboxamide ribonucleotide (down regulated in the S group) can be recycled via the purine pathway and purines are up-regulated in the control groups. Inosine and Inosine monophosphate were also among the top metabolites down regulated in the S group and contribute to precursor supply of purine metabolism.

**Figure 3:**
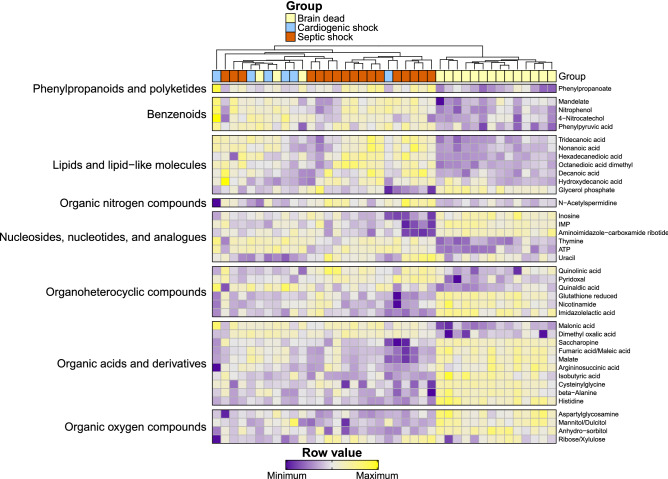
Heat map of the differentially abundant metabolites. Each row represents a metabolite, each column a patient color-coded according to its group. The overlying dendrogram is a graphical representation of patient similarity assessed by the euclidean distance: patients in the same cluster are more similar than patients in two separate clusters. The relative intensities were scaled by rows. Some samples were discarded due to quality checks. N = 40 patients analyzed for the metabolomics (Septic shock n = 17, cardiogenic shock n = 6, brain dead n = 17).

Hierarchical clustering of these metabolites found a clear discrimination of the metabolic pattern according to the S group (Fig. 3). Fatty acids and lipids were significantly higher in the S group (eTable 9).

### Enrichment analyses of proteomics data

**Figure 4:**
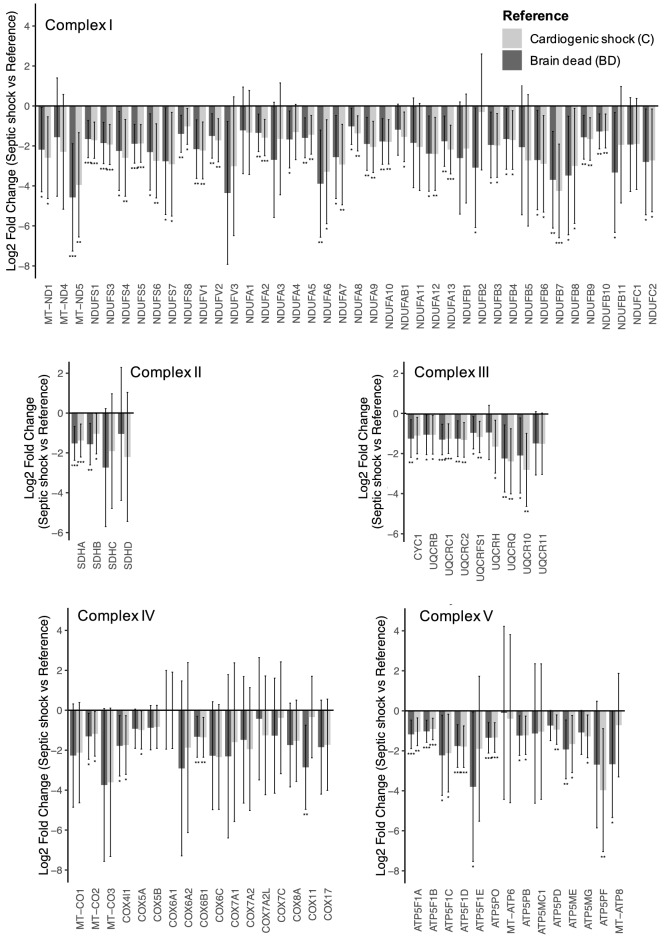
Mitochondrial complexes were decreased in the S group. The subunits of each mitochondrial complexes are represented. The subunits that were not identified in the proteomic dataset are not shown (Complex I : MT-ND2, MT-ND3, MT-ND6; Complex III : MY-CYB, UQCRHL; Complex IV : COX4l2, COX6B2, COX7B, COX7B2, COX8C, COX10, COX15; Complex V : ATP5MF). The bar represents the mean difference (Log 2 Fold Change) between the S group and the two control groups, the error bar represents the 95% confidence interval. N = 31 patients analyzed for the proteomics (Septic shock n = 12, cardiogenic shock n = 9, brain dead n = 10). *p < 0.05; **p < 0.01; ***p < 0.001 (Tukey’s post-hoc test, Group S versus Reference).

Enrichment analysis applying the ANCOVA global test were performed separately on the KEGG metabolic pathways and the KEGG signaling pathways databases respectively, shown in Table 2. In accordance with the PCA, the most down regulated pathways were metabolic pathways belonging to mitochondrial metabolism, being negatively impacted in the S group: oxidative phosphorylation (adjusted *p* = 0.007, Figure S7); branched chained amino acids degradation (adjusted *p* = 0.005, Figure S8); citrate cycle (adjusted *p* = 0.005, Figure S9); ketone body metabolism (adjusted *p* = 0.003, Figure S10) or beta oxidation (Fatty acid degradation, adjusted *p* = 0.007, Figure S11). The signaling pathways differentially abundant were the peroxisome proliferator-activated receptors (PPARs) signaling pathway (adjusted *p* = 0.007, Figure S12) and the retrograde endocannabinoid signaling pathway (adjusted *p* = 0.007). The robustness of these results was challenged with another enrichment analysis algorithm (GAGE algorithm) which confirmed the results (eTable 10 and 11, Figure S13). However, the pairwise comparisons lacked the power to detect small changes regarding ketone body metabolism, sulfur metabolism, arginine and proline metabolism, or differences in the signaling pathways database. The use of different algorithms for missing values imputation (eTable 12 and 13) and the complete case analysis (n = 555 proteins, eTable 14) showed consistent results, although PPARs signaling pathway was not found differently abundant in the complete case analysis. All the mitochondrial respiratory complexes were deficient in the S group (Fig. 4). Heat maps of the oxidative phosphorylation pathway (Figure S14) confirmed the partial clustering of the S group compared to the two control groups on the basis of mitochondrial respiratory complexes subunits.

**Table 2:** Enrichment analysis displaying Kyoto encyclopedia of genes and genomes (KEGG) pathways.

Pathways	Number of proteins			
	Pathway total (n)	Identified (n, %)	Raw *p* value	Adjusted *p* value
**KEGG metabolic pathways**				
Metabolism of ketone body	10	6 (60	< 0.001	0.003
Butanoate metabolism	28	12 (43)	< 0.001	0.003
Citrate cycle (TCA cycle)	30	26 (87)	< 0.001	0.005
Val, Leu and Ile degradation	48	34 (71)	< 0.001	0.005
Propanoate metabolism	34	22 (65)	< 0.001	0.005
Oxidative phosphorylation	133	93 (70)	< 0.001	0.007
Sulfur metabolism	10	7 (70)	0.001	0.007
Fatty acid degradation	44	27 (61)	0.001	0.007
Arginine and proline metabolism	50	21 (42)	0.001	0.007
**KEGG signaling pathways**				
PPARs signaling pathway	77	27 (35)	< 0.001	0.007
Retrograde endocannabinoid signaling	148	53 (36)	< 0.001	0.007

This enrichment analysis used the covariance analysis global test on the KEGG pathways database. Missing values were imputed using the probabilistic minimum imputation method. The pathways that are not considered differentially abundant (adjusted *p* value > 0.01) are not shown. Ile, Isoleucine; Leu, Leucine; Val, Valine; TCA, Tricyclic acid; PPARs, Peroxisome proliferator-activated receptors.

### Energy metabolism

The mitochondrial shutdown noticed in proteomic enrichment analysis was coupled with a significant decrease in mitochondrial carriers in the S group like the mitochondrial pyruvate carriers (MPC1 *p* = 0.031; MPC2 *p* = 0.021), the oxoglutarate/malate carrier (SLC25A11 *p* < 0.001), the voltage dependant anion-selective channels (VDAC1 *p* < 0.001; VDAC2 *p* = 0.014; VDAC3 *p* = 0.018), the adenine nucleotide translocator (SLC25A4 *p* < 0.001), and the carnitine-acylcarnitine translocase (SLC25A20 *p* = 0.02). Consistently, we observed metabolites accumulating upstream the mitochondria in the S group, like phosphoenolpyruvate (*p* = 0.003) or fatty acids, in particular octanedioic acid dimethyl (ANOVA *p* < 0.001, S versus C log2 fold change + 1.08 [0.05; 2.10], S vs BD + 1.84 [1.10; 2.58]) and hydroxydecanoic acid (ANOVA *p* < 0.001, S vs C 1.03 [0.28; 1.78], S vs BD 0.81 [0.27; 1.36]) which were more abundant in the S group regardless of the control group (Fig. 3, eTable 11). Other pathways highlighted by the proteomics were not reflected in the results obtained by the metabolomics. The decrease in branched-chain amino acids catabolism observed in proteomics (enrichment analysis) had no apparent resonance in metabolomics with no significant difference in the amino acids Valine (*p* = 0.12), Leucine (*p* = 0.07) or Isoleucine (*p* = 0.10). Similarly, despite a decrease in the ketone body metabolism enzymes in the S group, there was no significant difference regarding the sole ketone body identified, 3-Hydroxybutyrate (*p* = 0.18).

### Oxidative stress

**Figure 5:**
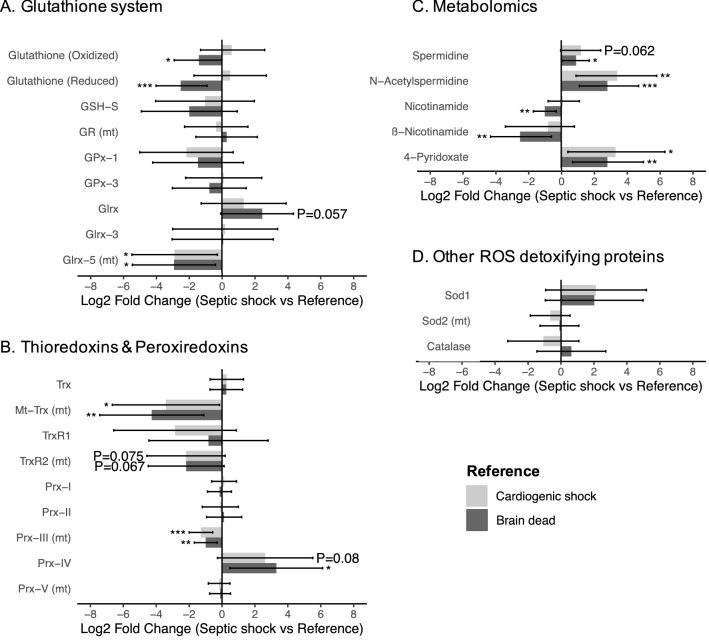
Alterations of the antioxidant and ROS detoxifying proteins and metabolites. The bar represents the mean difference (Log 2 Fold Change) between the S group and the two control groups, the error bar represents the 95% confidence interval. N = 31 patients analyzed for the proteomics (Septic shock n = 12, cardiogenic shock n = 9, brain dead n = 10) and N = 40 for the metabolomics (Septic shock n = 17, cardiogenic shock n = 6, brain dead n = 17). *p < 0.05; **p < 0.01; ***p < 0.001 (Tukey’s post-hoc test, Group S versus Reference). Glrx, Glutaredoxin; GPx, Glutathione peroxidase; GR, Glutathione disulfide reductase; GSH-S, Glutathione synthetase; Prx, Peroxiredoxin; Sod, Superoxide dismutase; Trx, Thioredoxin; TrxR, Thioredoxin reductase. The mitochondrial location of an enzyme is indicated by (mt).

Our results highlight another point of convergence between proteomics and metabolomics regarding oxidative stress related proteins and metabolites (Fig. 5, eTable 15). Mitochondrial isoforms of key antioxidant enzymes were depleted in the S group, such as the thioredoxin/peroxiredoxin system. The glutathione reductase (mitochondrial) was not differentially abundant (ANOVA *p* = 0.74), nor were the cytoplasmic glutathione peroxidase 1 (*p* = 0.17) and 3 (*p* = 0.61), the cytoplasmic glutaredoxin 1 (*p* = 0.07) and 3 (*p* > 0.99). Only the glutaredoxin 5 (mitochondrial) was decreased in the S group (*p* = 0.010). Metabolomics results mainly emphasized differences between the S and the BD groups, with a decrease in reduced glutathione (Tukey’s post-hoc test, S versus BD *p* = 0.001), a decrease in vitamin derivatives that are substrates for redox reactions (nicotinamide *p* = 0.002; ß-nicotinamide mononucleotide *p* = 0.007), an increase in the polyamines (spermidine *p* = 0.050; N-acetylspermidine *p* = 0.001) and in oxidative catabolites from vitamin B6 (4-pyridoxic acid *p* = 0.007).

## Discussion

This study provides a unique picture of the bioenergetics of human skeletal striated muscle in sepsis, with the integration of proteomics and metabolomics data providing a broad snapshot of the muscular state as a whole. The main result is the characterization of a septic shock signature associating a global alteration of mitochondrial metabolic pathways specifically in the septic shock group, with a metabolic reprogramming towards lipid accumulation and increased oxidative stress.

These results are innovative considering (i) the control groups being ICU patients and (ii) the advanced integration of proteomics and metabolomics data. The few studies that have performed muscle biopsies in critically ill patients have mainly compared septic shock individuals to healthy controls^7,33,34^. The originality of our study is the comparison of a septic group to two control groups of critically ill patients. Both control groups suffer a variety of muscle insults occurring in ICU^8^ (impaired perfusion and oxygen delivery, hyperglycemia, inflammation, bedridden status and muscle discharge, use of glucocorticoids or neuromuscular blocking agents), and are subjected to an inflammatory state resembling sepsis^14–16^. These insults make them more appropriate controls to single out the pathobiology of muscle dysfunction in septic shock than healthy patients. In muscle biopsies, only few inflammatory mediators could be detected, either because of a lack of sensitivity of the mass spectrometry technique used or the absence of a detectable inflammatory mediators in the examined muscle tissue.

Proteomics and metabolomics provide high dimensionality data on limited cohorts, which raises the challenge of the discrimination between background noise and biological phenomena to avoid false positive discoveries. We then focused on the biological processes that were significantly impacted in both datasets, to provide an integrated view of mechanisms occurring in skeletal muscles during sepsis. Part of our results is consistent with those of historical studies, increasing the external validity of our study. In the septic shock group, the exhaustion of antioxidant defenses was highlighted by proteomics (decreased in thioredoxin, peroxidredoxin, glutaredoxin systems) while metabolomics revealed a high level of oxidative stress (increased in polyamines^35^ and 4-pyridoxic acid^36^, decreased nicotinamide). Taken together, these results confirm the uncontrolled oxidative stress occurring in skeletal muscle during septic shock, coupled with a quantitative decrease of the mitochondrial respiratory chain, already extensively discussed^7,33^. However, our results emphasize a comprehensive impairment of the mitochondria, with a global impact on the metabolic pathways, energetic or not, as well as on the mitochondrial carriers allowing exchanges of metabolites through the mitochondrial membranes. The glycolysis (with cytoplasmic localization) was one of the rare unaffected metabolic pathways. The enzymes of anaplerosis, which replenish tricarboxylic acid cycle (Krebs cycle) intermediates, like the catabolism of branched-chain amino acids or the beta-oxidation show a significant reduction in the S group and highlight mitochondrial functional impairment. Accordingly, major metabolites accumulate in the S group upstream of their mitochondrial metabolism, such as phosphoenolpyruvate or lipid metabolites, suggesting the inability of skeletal muscle mitochondria of septic patients to metabolize fatty acids efficiently, as already hypothesized by Puthucheary et al.^34^. Lipid accumulation has also been associated with mitochondrial dysfunction in diaphragmatic muscle fibers^37^, and metabolic signature of the efficiency of fatty acid catabolism in the plasma of septic patients has been associated with increased survival^38,39^. Our results underline, at the muscle level, the lipidic metabolic signature associated with a poor prognosis at the plasma level. Moreover, these results are consistent with the metabolic adaptations thought to have a central role in other organ failures that occur during septic shock, like sepsis-induced acute kidney injury^40^.

The descriptive nature of our study prevents us any causal inference about the extensive metabolic reprogramming observed; however, the proteomics provides further insights. The PPARs signaling pathway, involved in metabolism, lipid transport and adipocyte differentiation, was differentially abundant in the S group. This result is consistent with pathological examinations that describe an adipocyte infiltration in human skeletal muscles during sepsis^41^. The PPAR family of transcription factors have wide-ranging critical regulatory and signal transducing roles in skeletal muscle and other tissues, from inflammation to fuel selection and contractile function. The modification of the PPARs signaling pathway could be related to skeletal muscle dysfunction in sepsis. Notably, the PPARγ agonists and the co-activator PGC-1ß have been implicated in the reduction of muscle protein catabolism during sepsis in animal models^42,43^. In Humans, PPARγ co-activator 1-alpha has been shown to be involved in mitochondrial biogenesis, a process associated with survival in septic shock^7^. Moreover, in a small sample phase I randomized controlled trial, pioglitazone (a PPARγ agonist) was associated with a decrease in inflammatory mediators in the serum such as interleukins-6 and 8 and TNF-alpha in young adults hospitalized in intensive care for sepsis^44^. These results underline the role of PPAR signaling in sepsis and that PPARs modulators hold the potential to be of a therapeutic interest in sepsis-related muscle dysfunction.

The limitations of this study should be mentioned. First, as a non-randomized study, differences in baseline characteristics of the groups could have induced selection bias. However, the external validity of the results and the consistent findings when comparing the S group to the two control groups increase our overall confidence in these results. Second, the sampling of different muscle groups due to ethical constraints may have induced a bias related to muscle fiber types. Historical data suggest fairly close ratios of type I fiber between the *vastus lateralis* (52%)^45^, *rectus abdominis* (46.1%)^46^ and *psoas major* (49.2%)^46^. Moreover, the relative abundances of myosin heavy chain, troponin, and tropomyosin isoforms were not different in the whole muscle sample. These results cannot substitute for the reference technique of fiber typing, consisting of the characterization of isolated muscle fibers, but the absence of global differences in specific isoforms coupled with historical data decrease the probability that a major difference in muscle composition has impacted our results. Third, at each experimental or analytical step, quality check may result in the exclusion of samples. This induces an attrition bias but is mandatory for the reliability of the results. Fourth, as an ancillary exploratory study, no study size was computed. The results, even though being considered as hypothesis generating, give a rare and extensive view on changes in muscle proteome and metabolome in sepsis compared to two control groups.

## Conclusion

Our study describes a septic shock signature in skeletal muscle that associates an uncontrolled oxidative stress and a fundamental modification of mitochondrial energetic metabolism with a metabolic shift towards lipid accumulation. The integration of these data allows the formulation of hypotheses and distinguishes the PPARs signaling pathway as a pathway of interest in sepsis-related muscle dysfunction. Future trials concerning muscle injury in critically ill septic patients should focus on this pathway to investigate its relationship with metabolic reprogramming.

## Supplementary Information

Supplementary Information 1.

Supplementary Information 2.

Supplementary Information 3.

Supplementary Information 4.

Supplementary Information 5.

Supplementary Information 6.

Supplementary Information 7.

Supplementary Information 8.

Supplementary Information 9.

Supplementary Information 10.

Supplementary Information 11.

Supplementary Information 12.

Supplementary Information 13.

Supplementary Information 14.

Supplementary Information 15.

## Data Availability

The mass spectrometry data have been deposited to the ProteomeXchange Consortium via the PRIDE partner repository for proteomics (identifier PXD0228839) and to the MassIVE repository for metabolomics (identifier MSV000088078) to be available on request.
