# Supplementary material for: Using a multiomics approach to unravel a septic shock specific signature in skeletal muscle
Source: Sci Rep. 2022 Nov 5;12:18776. doi: 10.1038/s41598-022-23544-8 (PMC9637214; doi:10.1038/s41598-022-23544-8)
Supplement: Supplementary file 1 — Supplementary Information 1. [file 41598_2022_23544_MOESM1_ESM.docx]

Supplemental digital material

Using a multiomics approach to unravel a septic shock specific signature in skeletal muscle

Baptiste Duceau*, Michael Blatzer*, Jean Bardon, Thibault Chaze, Quentin Giai Gianetto, Florence Castelli, François Fenaille, Lucie Duarte, Thomas Lescot, Christophe Tresallet, Bruno Riou, Mariette Matondo, Olivier Langeron, Pierre Rocheteau, Fabrice Chrétien**, Adrien Bouglé**

* These authors contributed equally to this work as co-first authors: Baptiste Duceau and Michael Blatzer

** These authors contributed equally to this work as co-last authors: Fabrice Chrétien and Adrien Bouglé

METHODS 4

Inclusion and exclusion criteria 4

Muscle biopsy and processing 6

Proteomics analysis by high-resolution liquid chromatography coupled to mass spectrometry (HR LC-MS) 6

Proteins extraction and samples preparation 6

High resolution LC-MS/MS analyses (Proteomics) 8

Proteins identification 9

Metabolomics analysis by liquid chromatography coupled to high-resolution mass spectrometry (LC-HRMS) 9

Metabolite extraction and sample preparation 9

LC- HRMS analysis (Metabolomics) 10

Metabolite identification 12

Bioinformatics analyses and missing values imputation 13

Enrichment Analysis 15

The ANCOVA (Analyse of Covariance) Global test 16

The GAGE (Generally Applicable Gene-set Enrichment) algorithm 16

SUPPLEMENTAL TABLES 18

eTable 1. List of annotated metabolites by matching accurately measured masses and chromatographic retention times to an in-house reference library made from pure authentic standards. 18

eTable 2. List of the metabolites of interest validated by fragmentation. 28

eTable 3. Fiber typing using proteomics on whole muscle samples. 31

eTable 4. Tissue-associated proteins potentially responsible for contamination. 32

eTable 5. First twenty proteins contributing to the second principal component. 33

eTable 6. First twenty metabolites contributing to the second principal component. 34

eTable 7. Proteins differentially abundant between groups. 35

eTable 8. Metabolites differentially abundant between groups. 41

eTable 9. Fatty acids accumulation in skeletal muscle from septic patients. 43

eTable 10. Enrichment analysis using the GAGE Algorithm, Septic shock versus Cardiogenic shock. 44

eTable 11. Enrichment analysis using the GAGE Algorithm, Septic shock versus Brain Dead. 45

eTable 12. Enrichment analysis using the covariance analysis global test, missing values imputation with the maximum likelihood estimation algorithm. 47

eTable 13. Enrichment analysis using the covariance analysis global test, missing values imputation with the structured least squares algorithm. 49

eTable 14. Enrichment analysis using the covariance analysis global test, complete case analysis (n=555 proteins). 51

eTable 15. Proteomics and metabolomics related to oxidative stress. 52

SUPPLEMENTAL FIGURES 54

Figure S1. Quality check of the proteomics dataset. 54

Figure S2. Pre-processing of the proteomics dataset. 55

Figure S3. Pre-processing of the metabolomics dataset. 56

Figure S4. Distribution of the quantity of proteins after imputation. 56

Figure S5. Scatterplot representing the imputed mean value depending the measured mean value for each protein. 57

Figure S6. Principal component analysis of the proteomics and metabolomics datasets stratified by sex, age and severity score. 57

Figures S7 to S12. Representation of the proteome and the metabolome on the significantly altered KEGG pathways. 58

Figure S13. Pairwise enrichment analysis using the GAGE algorithm. 59

Figure S14. Heat map of the oxidative phosphorylation pathway (proteomics dataset). 59

# METHODS

## Inclusion and exclusion criteria

Common inclusion criteria for all groups:

All the patients must be affiliated or beneficiary of the French social security system.

Hospitalization in an intensive care unit.

Inclusion criteria specific to the septic shock group:

- Septic shock as defined by Singer *et al.* [1] i.e. active infection and hemodynamic failure despite volume expansion (at least 30ml.kg^-1^), requiring the introduction vasopressors to maintain a mean arterial pressure ≥65 mmHg, as well as hyperlactatemia≥2mmol.L^-1^.
- Intra-abdominal infection identified as responsible for the sepsis.
- Surgical intervention required to control the infection site

Inclusion criteria specific to the cardiogenic shock group:

- Refractory cardiogenic shock defined as circulatory failure with signs of hypoperfusion, related to cardiac dysfunction, with systolic blood pressure below 90 mmHg or mean arterial pressure below 65 mmHg, in the presence of pulmonary congestion or elevated cardiac filling pressures.
- Requiring surgical placement of Extra-Corporeal Life Support (ECLS).

Inclusion criteria specific to the brain dead group:

- Established diagnosis of brain death:
  - Persistent coma with abolition of brain stem reflexes.
  - Abolition of spontaneous breathing confirmed by an apnea test.
  - The absence of confounding factors (intoxication, sedation, hypothermia).
  - A paraclinical test confirming brain death (lack of cerebral blood flow on a cerebral angiography or two electroencephalograms showing an isoelectric trace at 4 hours interval).
- Patient not registered in the French register of organ donation refusals.
- Multiorgan retrieval procedure accepted by the next-of-kin.

Common exclusion criteria for all groups:

- Patient with preexisting neuromuscular disease.
- Patient under 18 years of age.
- Pregnancy.

Exclusion criteria specific to the brain dead group:

- Patient enlisted on the French national register of organ donation refusals.

## Muscle biopsy and processing

The collection of muscle samples was carried out during a surgical intervention. We aimed to be as minimally invasive as possible to meet the objectives of the study. It was therefore planned to take muscle samples of the smallest possible volume (2 cm^3^) without making any other incision than those realized for the surgery. The *rectus abdominis* muscle was harvested during laparotomy surgeries in the septic shock group. The *vastus lateralis* muscle was harvested during the surgical approach of the iliac vessels during extracorporeal life support implantation in the cardiogenic shock group. The *psoas major* was harvested during multi-organ retrieval procedure in the brain dead group.

After the surgical biopsy, the muscle samples were stored in a storage medium (F12 Nutri Mix [Thermo Fisher Scientific, Waltham, Massachusetts, USA]; 1% HEPES buffer) at 4°C during the transfer to the Institut Pasteur by dedicated courier, and processed within 12 hours. After gross examination on ice, the non-muscular tissues (adipose tissue, tendons) were carefully removed, to ensure that subsequent analyses would be as specific as possible to skeletal muscle tissue. The muscle was subsampled for the different subsequent protocols. These subsamples were snap-frozen in liquid nitrogen, then preserved at -80°C until further use.

## Proteomics analysis by high-resolution liquid chromatography coupled to mass spectrometry (HR LC-MS)

### Proteins extraction and samples preparation

Proteins extraction was performed at the end of the inclusions, simultaneously for all the samples, to avoid any batch effect.

Muscle samples stored at -80°C were weighed and mechanically lysed by glass beads (FastPrep-24™, MP Biomedicals, California, USA, 5000 rpm for 30 sec) in lysis buffer (100 mM Tris pH 8.0 + 10 mM tris (2-carboxyethyl) phosphine (TCEP)). A weight/volume ratio of 5 mg/µL was observed for all lysed samples. After FastPrep™ lysis, the supernatant is recovered and the beads were rinsed with 100 μL of lysis buffer.

To facilitate protein extraction from muscle cells, 2% sodium dodecyl sulfate (SDS) was added. The samples were sonicated three times (VibraCell 75186, Thermo Fisher Scientific, 10 pulses of 80% amplitude). Debris is removed by centrifugation at 18.000 g at 4°C for 20 min. The proteins from the supernatant were delipidated and precipitated by phase partition (chloroform-methanol-water precipitation, 4:2:3). Briefly, 4 volumes of methanol (-20°C) and 2 volumes of chloroform (-20°C) were added, the samples were mixed quickly and 3 volumes of water were added. After centrifugation at 5000 g for 10 min, a white precipitate forms and a polar–apolar interface was clearly visible. The supernatant (chloroform) was removed and 500 μl of methanol (-20°C) was added. A protein precipitate forms and the pellet was washed three times with methanol (-20°C). The proteins were precipitated by another centrifugation (7000 g, 5 min). The pellet was air dried and suspended with 40 µl of 100 mM NaOH. The proteins were denatured with 250 μL of 8 M urea, 100 mM Tris, pH 8.0. The samples were frozen at this step in order to perform protein digestion in a limited number of batches.

The samples were thawed on ice and centrifuged for 20 min at 20.000 g. The supernatant was transferred to a new tube. The protein concentration of each sample was determined by a microBCA colorimetric assay (Pierce™, Thermo Fisher Scientific). Sixty µg of proteins were used for the proteomic analysis.

The disulfide bonds of the proteins were reduced (5 mM TCEP) for 30 min at 23°C then alkylated (20 mM iodoacetamide, 30 min, 23°C in the dark). Subsequently, 1 µg of endoproteinase LysC (Mass Spectrometry Grade, Promega) was added for the first digestion step (incubation for 4 hours at 30°C). The sample was diluted with 100 mM ammonium bicarbonate (NH_4_HCO_3_) to reduce the urea concentration to 1 M. The digestion of the proteins was completed for 12 hours at 37°C after adding 1 μg of Trypsin (Mass Spectrometry Grade, Promega).

The peptides generated by the double digestion were purified by solid-phase extraction (SPE) on a stage tip according to the Rappsilber *et al*. method [2]. The peptides were eluted from the phase with 80% acetonitrile and then dried. The dry samples were taken up in 60 µl of loading buffer (2% acetonitrile, 0.1% formic acid) and an optical density spectrum was read by NanoDrop™ (Thermo Fisher Scientific) to certify the quality of the material.

### High resolution LC-MS/MS analyses (Proteomics)

Mass spectrometric analysis was conducted on an Orbitrap Q Exactive Plus mass spectrometer (Thermo Fisher Scientific) coupled to an Easy1200 Proxeon liquid chromatograph (Thermo Fisher Scientific).

The equivalent of 1 μg of peptides was loaded (buffer A, 0.1% formic acid) then separated on a homemade C_18_ 50 cm capillary column with a picotip silica emitter (Dr. Maisch GmbH, Ammerbuch-Entringen, Germany). A 240 min elution gradient is applied (buffer B, 80% acetonitrile and 0.1% formic acid) to separate the peptides and allow their analysis in a mass spectrometer with an Orbitrap analyzer (Q-Exactive +, Thermo Fisher Scientific). Mass spectra were obtained in data-dependent acquisition mode, the 10 most intense precursors (identified during MS1) were selected, fragmented (HCD, Higher Collisional Dissociation fragmentation) and analyzed in the orbital cluster (MS2).

### Proteins identification

All the files (.raw) acquired on the MS instrument for the samples were analyzed using MaxQuant (v. 1.5.3.8) and its implemented Andromeda search algorithm [3]. MaxQuant allows the identification and quantification of proteins whose peptides have been identified by LC-MS/MS. Protein research was performed against the Uniprot human database [4] (71 522 entries, accessed on October 25, 2017). The minimum peptide length was specified to be 7 amino acids. Identification at the peptide and protein level was defined for a false discovery rate of 0.01. A minimum of two peptides were required for protein identification and quantification.

## Metabolomics analysis by liquid chromatography coupled to high-resolution mass spectrometry (LC-HRMS)

### Metabolite extraction and sample preparation

Metabolite extraction was performed at the end of the inclusions, simultaneously for all the samples, to avoid any batch effect. Muscle tissue metabolites extraction was performed from ~20 mg of tissue. Samples were resuspended in 170µL of ultrapure water, and then sonicated 5 times for 10 sec using a sonication probe (VibraCell™, Bioblock Scientific, Illkirch, France). At this step, 20µL of each sample were withdrawn for further determining the total protein concentration (colorimetric quantification / Pierce^TM^ BCA Protein Assay Kit, Thermo Fisher Scientific). A volume of 350µL of methanol containing internal standards at 3.75µg/mL (Dimetridazole, AMPA, MCPA, Dinoseb [Sigma-Aldrich, Saint-Quentin Fallavier, France]) was added to the remaining 150µL of tissue lysate. Cell debris were then removed by centrifugation for 15 min at 4°C and 20,000g. The resulting samples were then left on ice for 90 min until complete protein precipitation. After a final centrifugation step at 20,000 g for 15 min at 4°C, supernatants were recovered and split into two equal aliquots for C18 and HILIC analyses, respectively. Resulting aliquots were then dried under a stream of nitrogen using a TurboVap instrument (Thermo Fisher Scientific) and stored at −80 °C until analysis. Prior to LC-MS analysis, dried extracts were resuspended to reach a fixed protein concentration (equivalent to 20mg/mL) using variable volumes of H_2_O/Acetonitrile (95:5, v/v), containing 0.1% formic acid and the external standards (mixture of ^13^C-glucose and ^15^N-aspartate at 200 µg/ml, ethylmalonic acid at 30 µg/ml, amiloride at 100 µg/ml, prednisone, atropine sulfate and metformin at 10 µg/ml, colchicine and imipramine at 5 µg/ml) or 10 mM ammonium carbonate pH 10.5 containing the external standards (see above) / acetonitrile (40:60, v/v) for metabolite analysis using C18 and ZIC-pHILIC columns, respectively. After reconstitution, the tubes were vortexed and incubated in an ultrasonic bath for 5 min and then centrifuged at 20,000 g for 15 min at 4°C. Supernatant was transferred into 0.2 mL vials. A quality control (QC) sample was obtained by pooling 20 µL of each sample preparation. The QC sample was injected every 10 samples in order to evaluate the signal variations of any metabolite.

### LC- HRMS analysis (Metabolomics)

The ultra-high performance liquid chromatographic (UHPLC) separation was performed on a Hypersil GOLD C18 1.9 µm, 2.1 mm x 150 mm column (RP) at 30°C (Thermo Fisher Scientific), and high performance liquid chromatographic (HPLC) separation was performed on a Sequant ZIC-pHILIC 5µm, 2.1 x 150 mm (HILIC) at 15°C (Merck, Darmstadt, Germany). All chromatographic systems were equipped with an on-line prefilter (Thermo Fisher Scientific). Experimental settings for each LC-MS condition are described below. Mobile phases for RP columns were 100% water in A and 100% acetonitrile in B, both containing 0.1% formic acid. Regarding HILIC, phase A consisted of an aqueous buffer of 10 mM ammonium carbonate in water adjusted to pH 10.5 with ammonium hydroxide, whereas pure acetonitrile was used as solvent B. Chromatographic elutions were achieved under gradient conditions as follows: (i) RP-based system: the flow rate was set at 500 µL/min. The elution consisted of an isocratic step of 2 min at 5% phase B, followed by a linear gradient from 5% to 100% of phase B over the next 11 min. These proportions were kept constant for 12.5 min before returning to 5% B for 4.5 min. (ii) HILIC-based system: the flow rate was 200 µL/min. Elution started with an isocratic step of 2 min at 80% B, followed by a linear gradient from 80% to 40% of phase B from 2 to 12 min. The chromatographic system was then rinsed for 5 min at 0% of phase B, and the run ended with an equilibration step of 15 min (80% of phase B).

LC-HRMS analyses were performed using a U3000 liquid chromatography system coupled to an Exactive mass spectrometer (from Thermo Fisher Scientific) fitted with an electrospray source operated in the positive and negative ion modes. The software interface was Xcalibur (version 2.1, Thermo Fisher Scientific). The mass spectrometer was calibrated before each analysis in both ESI polarities using the manufacturer’s predefined methods and recommended calibration mixture provided by the manufacturer (external calibration). The Exactive mass spectrometer was operated with capillary voltage at -3 kV in the negative ionization mode and 5 kV in the positive ionization mode and a capillary temperature set at 280°C. The sheath gas pressure and the auxiliary gas pressure were set, respectively, at 60 and 10 arbitrary units with nitrogen gas. The mass resolution power of the analyzer was 50,000, full width at half maximum (FWHM) at m/z 200, for singly charged ions. The detection was achieved from m/z 85 to 1000 for RP conditions in the positive ionization mode and from m/z 50 to 1000 for HILIC conditions in the negative ionization mode.

### Metabolite identification

The pre-processing step aimed to ensure further accurate identification of metabolites and to avoid mis-assignments or background noise. All raw data were manually inspected using the Qualbrowser module of Xcalibur version 2.1 (Thermo Fisher Scientific). Raw files were first-of-all converted to mzXML format using the MSConvert software. Automatic peak detection and integration were performed using the XCMS software package (W4M platform [5]) which returned a data matrix containing m/z and retention time values of features together with their concentrations expressed in arbitrary units (i.e., areas of chromatographic peaks).

XCMS features were thereafter filtered according to the following criteria:

(i) the correlation between dilution factors of QC samples and areas of chromatographic peaks (filtered variables should exhibit coefficients of correlation above 0.7 in order to account for metabolites occurring at low concentrations and which are not detected anymore in the most diluted samples),

(ii) repeatability (the coefficient of variations obtained for chromatographic peak areas of QC samples should be below 30%), and

(iii) the ratio of chromatographic peak area of biological samples to blank samples above a value of 3.

Optionally, if necessary, chromatographic peak areas of each variable present in the XCMS peak lists were normalized using the locally estimated scatterplot smoothing algorithm (a local regression smoothing algorithm) in order to remove analytical drift induced by clogging of the ESI source observed in the course of analytical runs.

Features were annotated by our spectral database according to accurately measured masses and chromatographic retention times obtained from ~1000 pure authentic standards [6]. Confirmation of metabolite annotation was then accomplished by running additional LC-MS/MS experiments using a Dionex Ultimate chromatographic system combined with a Q-Exactive mass spectrometer (Thermo Fisher Scientific) operated under non-resonant collision-induced dissociation conditions using higher-energy C-trap dissociation (HCD). Resulting spectra were matched to those included in our in-house spectral database. To be identified, ions had to match at least 2 orthogonal criteria (accurately measured mass, isotopic pattern, MS/MS spectrum and retention time) to those of an authentic chemical standard analyzed under the same analytical conditions, as proposed by the Metabolomics Standards Initiative [7].

## Bioinformatics analyses and missing values imputation

Quality checks of the proteomics are presented in Figure S1. Proteomics samples with a high number of missing values and samples with too low level of correlation of observed values with others were excluded. A principal component analysis was also conducted on the sixty more abundant proteins for identifying outliers. On the 51 proteomics samples, 16 were dismissed after quality check. Four patients were excluded because of missing informed consent.

At the filtering step, only proteins with intensity values measured in at least three samples were conserved to avoid misidentified proteins and to ensure minimum replicability of the experiments. After log2 transformation of the intensities, a median centering normalization was performed to remove any source of systematic variability that may have bias the quantification of peptides (overall protein concentration, pipetting variation, batch effect).

High-throughput data usually generate missing values, even if samples with more than 70% of missing values were excluded. Several methods were used to account for missing values in the proteomic dataset.

The main analysis was conducted using the probabilistic minimum imputation method [8], adapted for the “Missing Not-At-Random” (MNAR) missing values hypothesis. In analytical chemistry, the limit of quantification (LOQ) represents the instrument detection limit, i.e. the lowest concentration of the analyte for which the analytical method is capable of giving a value quantified with a good precision. The LOQ induces a left-censoring in the dataset, as any analyte being under the LOQ fails to be measured. In the probabilistic minimum imputation method, the value imputed is a random draw from a Gaussian distribution with mean=LOQ/2 and standard deviation = median standard deviation of the dataset. Using a Gaussian distribution allows to add a random noise in the imputed data to avoid the bias induced by multiple replacements by a single value.

However, the MNAR missing values hypothesis is not strictly verified in proteomics. Many factors can lead to “Missing At-Random” (MAR) or “Missing Completely-At-Random” (MCAR) missing values, from biochemical and analytical mechanisms (poorly prepared samples, degradation of the sample, poor cleavage of the peptides) to experimental (low ionization efficiency during LC-MS) or bioinformatics mechanisms (peptide misidentification, ambiguous matching) [8, 9]. To account for MAR/MCAR missing values hypothesis, enrichment analyses were refined with two other imputation algorithms adapted for MCAR missing values hypothesis: imputation with the maximum likelihood estimation algorithm [10] and imputation with an adaptation of the LSimpute algorithm [11] (structured least squares algorithm).

Finally, a complete case analysis without any imputation was performed (removal of the proteins with at least one missing value).

The proportion of missing values was low in metabolomics (less than 3%), then only the probabilistic minimum imputation method was used. Given this low proportion of missing values, it is highly unlikely that another imputation method would lead to different results.

The supplemental Figures S2 and S3 show the results of the transformations applied to the proteomic and metabolomic datasets as well as the missing data imputation using the probabilistic minimum imputation method.

## Enrichment Analysis

Enrichment analyses are used to generate biological insight from the proteomics dataset. The goal of enrichment analysis is to assess which relevant functional or mechanistic biological processes are over-represented and to which biological processes could be significantly altered between two experimental groups. The Kyoto Encyclopedia of Genes and Genomes [12, 13] (KEGG) pathways was used for all enrichment analyses. Using the KEGG database, the proteins are grouped in functional sets (“KEGG pathways”) involved in a biological phenomenon, a metabolic cascade, a synthesis or degradation pathway, or a pathological process. Then, the comparison between two experimental groups is carried out at the pathway level and not at the protein level. The functionnal class scoring (FCS, second generation of enrichment analysis) is the main approach used for enrichment analysis in our study. The FCS aims to infer which pathway are differentially abundant between two conditions by small coordinated changes in the quantity of proteins belonging to the pathway. Two different FCS algorithms were used, to validate the robustness of the results found. The analysis of covariance global test [14, 15] was used because it allows the comparison of three groups, and preserve the highest power. Then, we compared the groups two by two using the generally applicable gene-set enrichment (GAGE [16]) algorithm which is a slightly optimized gene set enrichment analysis with more robust results [17].

To account for multiple comparisons, the pathway level p-values were adjusted in the two algorithms (covariance analysis global test and GAGE) by a Benjamini-Hochberg procedure [18], with a false discovery rate of 0.01.

### The ANCOVA (Analyse of Covariance) Global test

In order to analyze the three groups simultaneously, a global method that perform the analysis in a single step was also used (GlobalAncova function of the eponymous R package) [14, 15]. The covariance analysis global test is carried out by comparison of linear models via the extra sum of squares principle. To account for multiple comparisons, the pathway level p-values are adjusted by a Benjamini-Hochberg procedure [17].

### The GAGE (Generally Applicable Gene-set Enrichment) algorithm

The GAGE algorithm [16] is an enrichment analysis widely used in transcriptomic data. Adapted to proteomic, it aims to infer which protein-sets are differentially abundant between two conditions, without any filtering on predefined cut-off. Indeed, small coordinated protein changes in a pathway can result in a major biological effect even if these changes are not statistically significant at the protein level. Essentially, the GAGE method uses three major steps to compare two conditions: (1) Assessment of the protein differential expression between the samples from the two conditions; for each protein, a protein level p-value is computed. (2) For each protein set, the protein level p-value are summarized using a meta-test to compute a protein-set level p-value. In this work, the meta-test used is a non-parametric Kolmogorov-Smirnov statistic, the null hypothesis H0 being: “The distribution in the protein constituting the protein-set is not different to the distribution of the differential abundance in the entire dataset”. (3) To account for multiple comparisons, the protein-set level p-value is adjusted by a Benjamini-Hochberg procedure [18].

# SUPPLEMENTAL TABLES

## eTable 1. List of annotated metabolites by matching accurately measured masses and chromatographic retention times to an in-house reference library made from pure authentic standards.

| **Metabolite** | **Ion Detected** | ***m/z*** | **RT (min)** | **Chromatographic Column** |
| --- | --- | --- | --- | --- |
| **Amino acids, peptides, and analogues** | |  |  |  |
| Alanine | [M-H]- | 88.0404 | 7.91 | ZIC-pHILIC |
| beta-Alanine | [M-H]- | 88.0404 | 7.61 | ZIC-pHILIC |
| Aminoisobutyric acid | [M-H]- | 102.0560 | 6.50 | ZIC-pHILIC |
| Serine | [M-H]- | 104.0353 | 8.05 | ZIC-pHILIC |
| Proline | [M-H]- | 114.0560 | 6.39 | ZIC-pHILIC |
| Guanidineacetic acid | [M-H]- | 116.0466 | 8.53 | ZIC-pHILIC |
| Valine | [M-H]- | 116.0717 | 5.17 | ZIC-pHILIC |
| Homoserine/Threonine | [M-H]- | 118.0509 | 7.46 | ZIC-pHILIC |
| Cysteine | [M+H]+ | 122.0272 | 0.94 | Hypersil GOLD C18 |
| Pyroglutamic acid | [M-H]- | 128.0353 | 5.05 | ZIC-pHILIC |
| N-Acetyl-alanine | [M-H]- | 130.0510 | 3.70 | ZIC-pHILIC |
| Hydroxy-proline | [M-H]- | 130.0510 | 8.16 | ZIC-pHILIC |
| Creatine | [M-H]- | 130.0621 | 7.91 | ZIC-pHILIC |
| Isoleucine | [M-H]- | 130.0873 | 4.06 | ZIC-pHILIC |
| Leucine | [M-H]- | 130.0873 | 3.59 | ZIC-pHILIC |
| Asparagine | [M-H]- | 131.0461 | 7.90 | ZIC-pHILIC |
| Ornithine | [M-H]- | 131.0825 | 9.38 | ZIC-pHILIC |
| Aspartic acid | [M-H]- | 132.0302 | 8.89 | ZIC-pHILIC |
| alpha-Aminoadipic acid | [M-H]- | 142.0509 | 3.48 | ZIC-pHILIC |
| N-Isobutyrylglycine | [M-H]- | 144.0666 | 2.68 | ZIC-pHILIC |
| Acetamidobutanoic acid | [M-H]- | 144.0666 | 3.72 | ZIC-pHILIC |
| Glutamine | [M-H]- | 145.0617 | 7.86 | ZIC-pHILIC |
| Lysine | [M-H]- | 145.0981 | 9.72 | ZIC-pHILIC |
| Glutamic acid | [M-H]- | 146.0458 | 8.88 | ZIC-pHILIC |
| Acetyl-serine | [M-H]- | 146.0458 | 5.55 | ZIC-pHILIC |
| Methionine/S-ethyl-L-cysteine | [M-H]- | 148.0437 | 3.82 | ZIC-pHILIC |
| Histidine | [M-H]- | 154.0621 | 7.46 | ZIC-pHILIC |
| N-Acetyl-norvaline/N-Acetyl-valine | [M-H]- | 158.0822 | 2.22 | ZIC-pHILIC |
| N-Acetyl-Cystein | [M+H]+ | 164.0379 | 1.84 | Hypersil GOLD C18 |
| Phenylalanine | [M+H]+ | 166.0862 | 2.38 | Hypersil GOLD C18 |
| Prolylglycine/Glycylproline | [M+H]+ | 173.0923 | 1.04 | Hypersil GOLD C18 |
| N-Acetylornithine | [M-H]- | 173.0930 | 6.45 | ZIC-pHILIC |
| Arginine | [M-H]- | 173.1044 | 10.12 | ZIC-pHILIC |
| N-Acetylaspartate | [M-H]- | 174.0407 | 8.76 | ZIC-pHILIC |
| Citrulline | [M-H]- | 174.0884 | 8.26 | ZIC-pHILIC |
| Allantoic acid | [M+H]+ | 177.0612 | 0.85 | Hypersil GOLD C18 |
| Hippuric acid/Succinoylpyridine | [M-H]- | 178.0509 | 2.24 | ZIC-pHILIC |
| Cysteinylglycine | [M+H]+ | 179.0485 | 1.16 | Hypersil GOLD C18 |
| Tyrosine/Amino-hydroxyphenyl propanoic acid | [M-H]- | 180.0666 | 5.72 | ZIC-pHILIC |
| Threo-Phenylserine | [M-H]- | 180.0667 | 2.85 | ZIC-pHILIC |
| Phosphoserine | [M-H]- | 184.0016 | 8.93 | ZIC-pHILIC |
| N-Acetyl-glutamine | [M-H]- | 187.0724 | 5.52 | ZIC-pHILIC |
| N6-Acetyl-lysine | [M-H]- | 187.1088 | 6.01 | ZIC-pHILIC |
| N-Acetylglutamate | [M-H]- | 188.0564 | 8.33 | ZIC-pHILIC |
| N-Acetyl-methionine | [M-H]- | 190.0543 | 2.27 | ZIC-pHILIC |
| Methylhippuric acid | [M-H]- | 192.0666 | 1.71 | ZIC-pHILIC |
| Caprylolyglycine | [M-H]- | 200.1293 | 1.43 | ZIC-pHILIC |
| Tryptophan | [M-H]- | 203.0825 | 4.06 | ZIC-pHILIC |
| N-Acetyl-phenylalanine | [M-H]- | 206.0822 | 1.61 | ZIC-pHILIC |
| N-Acetyl-arginine | [M-H]- | 215.1149 | 9.85 | ZIC-pHILIC |
| Valinylproline | [M+H]+ | 215.1395 | 1.67 | Hypersil GOLD C18 |
| Anserine | [M-H]- | 239.1150 | 7.26 | ZIC-pHILIC |
| N-Acetyl-tryptophan | [M-H]- | 245.0933 | 2.30 | ZIC-pHILIC |
| gamma-Glutamylcysteine | [M+H]+ | 251.0697 | 1.17 | Hypersil GOLD C18 |
| Alanyl-tyrosine | [M+H]+ | 253.1187 | 1.95 | Hypersil GOLD C18 |
| Phenylacetyl-glutamine | [M+H]+ | 265.1190 | 5.33 | Hypersil GOLD C18 |
| Ergothioneine | [M-H]- | 275.0918 | 7.25 | ZIC-pHILIC |
| Saccharopine | [M-H]- | 275.1251 | 9.29 | ZIC-pHILIC |
| Aspartyl-phenylalanine | [M+H]+ | 281.1139 | 4.70 | Hypersil GOLD C18 |
| Argininosuccinic acid | [M-H]- | 289.1155 | 9.53 | ZIC-pHILIC |
| N-Acetylaspartylglutamate | [M-H]- | 303.0835 | 9.86 | ZIC-pHILIC |
| Folic acid | [M-H]- | 440.1326 | 10.15 | ZIC-pHILIC |
| **Di/Tricarboxylic acids and derivatives** | |  |  |  |
| Oxalic acid | [M-H]- | 88.9880 | 10.15 | ZIC-pHILIC |
| Fumaric acid/maleic acid | [M-H]- | 115.0036 | 9.37 | ZIC-pHILIC |
| Dimethyl oxalic acid | [M-H]- | 117.0190 | 4.57 | ZIC-pHILIC |
| Succinic acid/Methylmalonic acid | [M-H]- | 117.0190 | 9.01 | ZIC-pHILIC |
| Diglycolic acid | [M-H]- | 133.0140 | 8.90 | ZIC-pHILIC |
| Citric acid/Isocitric acid | [M-H]- | 191.0200 | 10.46 | ZIC-pHILIC |
| **Keto acids and derivatives** |  |  |  |  |
| Oxovaleric acid | [M-H]- | 115.0401 | 2.13 | ZIC-pHILIC |
| Methyl-oxovaleric acids * | [M-H]- | 129.0558 | 1.62 | ZIC-pHILIC |
| Shikimic acid | [M-H]- | 173.0454 | 8.23 | ZIC-pHILIC |
| **Other organic acids** |  |  |  |  |
| Glycolic acid | [M-H]- | 75.0087 | 5.69 | ZIC-pHILIC |
| Isobutyric acid | [M-H]- | 87.0451 | 2.68 | ZIC-pHILIC |
| Lactic acid | [M-H]- | 89.0243 | 4.38 | ZIC-pHILIC |
| Malonic acid | [M-H]- | 103.0036 | 9.36 | ZIC-pHILIC |
| Taurine | [M-H]- | 124.0073 | 7.76 | ZIC-pHILIC |
| Pipecolinic acid | [M+H]+ | 130.0865 | 0.83 | Hypersil GOLD C18 |
| Malic acid | [M-H]- | 133.0142 | 9.41 | ZIC-pHILIC |
| Phosphonoacetic acid | [M-H]- | 138.9801 | 9.20 | ZIC-pHILIC |
| O-Phosphorylethanolamine | [M-H]- | 140.0118 | 9.21 | ZIC-pHILIC |
| Phosphoenolpyruvic acid | [M-H]- | 166.9751 | 10.17 | ZIC-pHILIC |
| Indoxyl sulfate | [M-H]- | 212.0024 | 2.70 | ZIC-pHILIC |
| Carnosine | [M-H]- | 225.0993 | 8.01 | ZIC-pHILIC |
| N-Acetyl-Carnosine | [M+H]+ | 269.1246 | 1.00 | Hypersil GOLD C18 |
| S-Lactoylglutathione | [M+H]+ | 380.1124 | 1.46 | Hypersil GOLD C18 |
| Glutathione oxidized | [M-H]- | 611.1444 | 10.23 | ZIC-pHILIC |
| **Benzenoids** |  |  |  |  |
| Benzyl-alcohol | [M+H-H2O]+ | 91.0546 | 5.71 | Hypersil GOLD C18 |
| Benzoic acid/Hydroxybenzaldehyde | [M-H]- | 121.0297 | 2.20 | ZIC-pHILIC |
| Phenylacetic acid/Toluic acid | [M-H]- | 135.0451 | 2.20 | ZIC-pHILIC |
| Salicylamide | [M-H]- | 136.0404 | 1.55 | ZIC-pHILIC |
| Nitrophenol | [M-H]- | 138.0196 | 1.82 | ZIC-pHILIC |
| Mandelic acid/Hydroxyphenylacetic acid/Anisic acid/Resorcinol monoacetate | [M-H]- | 151.0400 | 2.16 | ZIC-pHILIC |
| 4-Nitrocatechol | [M-H]- | 154.0147 | 2.74 | ZIC-pHILIC |
| Phenylpyruvic acid | [M-H]- | 163.0401 | 1.61 | ZIC-pHILIC |
| Terephthalic acid | [M-H]- | 165.0192 | 8.29 | ZIC-pHILIC |
| Methylcatechol | [M-H]- | 169.0505 | 2.24 | ZIC-pHILIC |
| Homovanillic acid/Hydroxyphenyllactic acid | [M-H]- | 181.0506 | 3.60 | ZIC-pHILIC |
| Normetanephrine | [M-H]- | 182.0821 | 2.53 | ZIC-pHILIC |
| Methylhippuric acid/Phenylacetylglycine | [M-H]- | 192.0666 | 2.15 | ZIC-pHILIC |
| Methylhippuric acids | [M+H]+ | 194.0818 | 5.72 | Hypersil GOLD C18 |
| **Carbohydrates and carbohydrate conjugates** | |  |  |  |
| Glyceric acid | [M-H]- | 105.0193 | 6.43 | ZIC-pHILIC |
| Erythritol/Threitol | [M-H]- | 121.0507 | 5.27 | ZIC-pHILIC |
| Threonic acid | [M-H]- | 135.0299 | 7.45 | ZIC-pHILIC |
| Tartaric acid | [M-H]- | 149.0090 | 9.91 | ZIC-pHILIC |
| Pentoses (Ribose/Xylulose) | [M-H]- | 149.0454 | 5.01 | ZIC-pHILIC |
| Ketopentose (Xylulose/Arabinose) | [M-H]- | 149.0454 | 6.65 | ZIC-pHILIC |
| Arabitol/Adonitol | [M-H]- | 151.0611 | 6.48 | ZIC-pHILIC |
| Anhydro-sorbitol | [M-H]- | 163.0611 | 5.56 | ZIC-pHILIC |
| Glycerone phosphate | [M-H]- | 168.9907 | 9.10 | ZIC-pHILIC |
| Gluconic acid lactone | [M-H]- | 177.0404 | 8.02 | ZIC-pHILIC |
| Myo-inositol | [M-H]- | 179.0561 | 9.55 | ZIC-pHILIC |
| Gluconic acid/Galactonic acid | [M-H]- | 195.0509 | 8.24 | ZIC-pHILIC |
| Sugar alcohol (Mannitol/Dulcitol) | [M+Na]+ | 205.0686 | 0.91 | Hypersil GOLD C18 |
| Saccharic acid | [M-H]- | 209.0303 | 10.11 | ZIC-pHILIC |
| Pentoses phosphate † | [M-H]- | 229.0118 | 9.23 | ZIC-pHILIC |
| Muramic acid | [M-H]- | 250.0933 | 7.90 | ZIC-pHILIC |
| Hexose phosphate | [M-H]- | 259.0225 | 9.76 | ZIC-pHILIC |
| Mannitol phosphate | [M-H]- | 261.0381 | 9.03 | ZIC-pHILIC |
| Diphospho-glyceric acid | [M-H]- | 264.9521 | 10.22 | ZIC-pHILIC |
| Sedoheptulose phosphate | [M-H]- | 289.0332 | 9.51 | ZIC-pHILIC |
| N-Acetyl-glucosamine phosphate | [M-H]- | 300.0491 | 8.92 | ZIC-pHILIC |
| Glucosamine phosphate | [M-H]- | 304.0440 | 8.90 | ZIC-pHILIC |
| N-Acetylneuraminic acid | [M-H]- | 308.0988 | 7.99 | ZIC-pHILIC |
| Aspartylglycosamine | [M-H]- | 334.1256 | 7.70 | ZIC-pHILIC |
| Hexose bisphosphate | [M-H]- | 338.9888 | 10.29 | ZIC-pHILIC |
| Disaccharides ‡ | [M-H]- | 341.1089 | 8.94 | ZIC-pHILIC |
| Cellobiose | [M-H]- | 387.1144 | 8.92 | ZIC-pHILIC |
| Triholosides | [M-H]- | 503.1620 | 9.55 | ZIC-pHILIC |
| Uridine diphosphoglucuronic acid | [M-H]- | 579.0272 | 11.50 | ZIC-pHILIC |
| Maltotetraose | [M-H]- | 665.2148 | 9.98 | ZIC-pHILIC |
| **Alcohols and polyols** |  |  |  |  |
| Quinic acid | [M-H]- | 191.0561 | 7.50 | ZIC-pHILIC |
| Panthenol | [M-H]- | 204.1241 | 2.06 | ZIC-pHILIC |
| Pantothenic acid | [M-H]- | 218.1034 | 3.54 | ZIC-pHILIC |
| Myo-inositol bisphosphate | [M-H]- | 338.9888 | 10.78 | ZIC-pHILIC |
| **Other organic oxygen compounds** |  |  |  |  |
| Hydroxymethylfurfural | [M-H]- | 125.0244 | 1.94 | ZIC-pHILIC |
| Aminoacetophenone | [M-H]- | 134.0611 | 1.60 | ZIC-pHILIC |
| Kynurenine | [M-H]- | 207.0775 | 3.28 | ZIC-pHILIC |
| **Organoheterocyclic compounds** |  |  |  |  |
| Nicotinamide | [M-H]- | 121.0407 | 2.22 | ZIC-pHILIC |
| Hypoxanthine | [M-H]- | 135.0312 | 4.65 | ZIC-pHILIC |
| Methylnicotinamide | [M-H]- | 135.0564 | 1.71 | ZIC-pHILIC |
| Aminopyridine-carboxylic acid/Imidazoleacrylic acid | [M-H]- | 137.0356 | 5.39 | ZIC-pHILIC |
| Aminopyridine-carboxylic acid | [M-H]- | 137.0356 | 2.59 | ZIC-pHILIC |
| Methylnicotinamide | [M+H]+ | 137.0711 | 0.92 | Hypersil GOLD C18 |
| Methylimidazoleacetic acid | [M-H]- | 139.0511 | 3.74 | ZIC-pHILIC |
| Xanthine | [M-H]- | 151.0261 | 6.20 | ZIC-pHILIC |
| Orotic acid | [M-H]- | 155.0098 | 5.59 | ZIC-pHILIC |
| Imidazolelactic acid | [M-H]- | 155.0462 | 5.70 | ZIC-pHILIC |
| Allantoin | [M-H]- | 157.0365 | 7.81 | ZIC-pHILIC |
| Serotonin-hydrochloride | [M+H]+ | 160.0760 | 1.97 | Hypersil GOLD C18 |
| Quinolinic acid | [M-H]- | 166.0144 | 8.96 | ZIC-pHILIC |
| Pyridoxal | [M+H]+ | 168.0656 | 1.07 | Hypersil GOLD C18 |
| Pyridoxine | [M-H]- | 168.0665 | 3.79 | ZIC-pHILIC |
| Quinaldic acid | [M-H]- | 172.0404 | 2.23 | ZIC-pHILIC |
| Dehydroascorbic acid/Aconitic acid | [M-H]- | 173.0091 | 10.49 | ZIC-pHILIC |
| Pyridoxic acid | [M-H]- | 182.0459 | 1.62 | ZIC-pHILIC |
| Indoleacrylic acid | [M-H]- | 186.0560 | 2.74 | ZIC-pHILIC |
| Kynurenic acid | [M-H]- | 188.0357 | 1.64 | ZIC-pHILIC |
| dihydroxybenzenesulfonic acid | [M-H]- | 188.9863 | 11.56 | ZIC-pHILIC |
| Dimethyluric acids § | [M-H]- | 195.0525 | 2.87 | ZIC-pHILIC |
| Methoxyindoleacetic acid/Indolelactic acid | [M-H]- | 204.0667 | 2.81 | ZIC-pHILIC |
| Hydroxytryptophan | [M+H]+ | 221.0925 | 1.74 | Hypersil GOLD C18 |
| Biotin | [M+H]+ | 245.096 | 5.55 | Hypersil GOLD C18 |
| Ascorbic acid sulfate | [M-H]- | 254.9818 | 12.37 | ZIC-pHILIC |
| Glutathione reduced | [M+H]+ | 308.0913 | 1.17 | Hypersil GOLD C18 |
| beta-Nicotinamide ribonucleotide | [M-H]- | 316.0439 | 9.88 | ZIC-pHILIC |
| Riboflavin | [M+H]+ | 377.1459 | 5.40 | Hypersil GOLD C18 |
| Riboflavin-monophosphate | [M+H]+ | 457.1132 | 5.13 | Hypersil GOLD C18 |
| **Nucleosides, nucleotides, and analogues** | |  |  |  |
| Uracil | [M-H]- | 111.0200 | 2.97 | ZIC-pHILIC |
| Dihydrouracil | [M-H]- | 113.0356 | 3.22 | ZIC-pHILIC |
| Purine | [M-H]- | 119.036 | 2.49 | ZIC-pHILIC |
| Thymine | [M-H]- | 125.0356 | 2.35 | ZIC-pHILIC |
| Dihydrothymine | [M+H]+ | 129.0661 | 0.93 | Hypersil GOLD C18 |
| Adenine | [M-H]- | 134.0472 | 2.89 | ZIC-pHILIC |
| Guanidinobutyric acid | [M-H]- | 144.0777 | 8.17 | ZIC-pHILIC |
| Deoxyuridine | [M-H]- | 227.0675 | 2.76 | ZIC-pHILIC |
| Thymidine | [M-H]- | 241.0832 | 2.25 | ZIC-pHILIC |
| Uridine | [M-H]- | 243.0621 | 4.10 | ZIC-pHILIC |
| Deoxyinosine | [M+H]+ | 253.0932 | 1.50 | Hypersil GOLD C18 |
| Aminoimidazole-carboxamide ribofuranoside (AICAR) | [M-H]- | 257.0891 | 4.18 | ZIC-pHILIC |
| Adenosine | [M+H]+ | 268.1042 | 1.26 | Hypersil GOLD C18 |
| Inosine | [M+H]+ | 269.0883 | 1.36 | Hypersil GOLD C18 |
| Guanosine | [M-H]- | 282.0845 | 6.44 | ZIC-pHILIC |
| Methyladenosine | [M+H]+ | 282.1199 | 1.06 | Hypersil GOLD C18 |
| Xanthosine | [M-H]- | 283.0685 | 7.22 | ZIC-pHILIC |
| O-Methylinosine | [M+H]+ | 283.1042 | 1.88 | Hypersil GOLD C18 |
| N4-Acetylcytidine | [M+H]+ | 286.1040 | 2.21 | Hypersil GOLD C18 |
| Orotidine | [M-H]- | 287.0524 | 8.04 | ZIC-pHILIC |
| Uridine monophosphate (UMP) | [M-H]- | 323.0286 | 9.43 | ZIC-pHILIC |
| Aminoimidazole-carboxamide-ß-ribofuranosyl monophosphate | [M-H]- | 337.0556 | 9.01 | ZIC-pHILIC |
| Adenosine monophosphate (AMP) | [M-H]- | 346.0558 | 8.26 | ZIC-pHILIC |
| Inosine-monophosphate (IMP) | [M-H]- | 347.0398 | 9.73 | ZIC-pHILIC |
| Deoxyguanosine monophosphate (dGMP) | [M+H]+ | 348.0707 | 0.96 | Hypersil GOLD C18 |
| Guanosine monophosphate (GMP) | [M-H]- | 362.0509 | 9.98 | ZIC-pHILIC |
| Uridine diphosphate (UDP) | [M-H]- | 402.9950 | 10.02 | ZIC-pHILIC |
| Adenosine diphosphate (ADP) | [M-H]- | 426.0223 | 8.93 | ZIC-pHILIC |
| Guanosine diphosphate (GDP) | [M-H]- | 442.0172 | 10.58 | ZIC-pHILIC |
| Adenosine triphosphate (ATP) | [M-H]- | 505.9880 | 9.43 | ZIC-pHILIC |
| Guanosine triphosphate (GTP) | [M-H]- | 521.9835 | 11.12 | ZIC-pHILIC |
| Cyclic ADP-ribose (cADPR) | [M-H]- | 540.0538 | 8.64 | ZIC-pHILIC |
| Adenosine diphosphate ribose (ADPR) | [M-H]- | 558.0637 | 8.84 | ZIC-pHILIC |
| Uridine diphosphogalactose | [M-H]- | 565.0475 | 10.10 | ZIC-pHILIC |
| Guanosine diphospho-ß-fucose | [M-H]- | 588.0748 | 10.54 | ZIC-pHILIC |
| Guanosine diphosphohexose | [M-H]- | 604.0700 | 10.80 | ZIC-pHILIC |
| UDP-N-acetylglucosamine | [M-H]- | 606.0741 | 9.52 | ZIC-pHILIC |
| **Fatty acids and conjugates** |  |  |  |  |
| Isovaleric acid | [M-H]- | 101.0608 | 2.22 | ZIC-pHILIC |
| Hydroxybutyric acid | [M-H]- | 103.0400 | 3.10 | ZIC-pHILIC |
| Hexanoic acid | [M-H]- | 115.0764 | 1.59 | ZIC-pHILIC |
| Aminovaleric acid | [M-H]- | 116.0717 | 7.67 | ZIC-pHILIC |
| Hydroxy methylbutyric acids/Hydroxypentanoic acid | [M-H]- | 117.0557 | 2.39 | ZIC-pHILIC |
| Aminocaproic acid | [M-H]- | 130.0872 | 7.70 | ZIC-pHILIC |
| Hydroxyhexanoic acid | [M-H]- | 131.0713 | 1.86 | ZIC-pHILIC |
| Octanoic acid | [M-H]- | 143.1077 | 1.45 | ZIC-pHILIC |
| Hexanedioic acid/Dimethylsuccinic acid/Methylglutaric acid | [M-H]- | 145.0505 | 8.41 | ZIC-pHILIC |
| Mevalonic acid | [M-H]- | 147.0662 | 2.68 | ZIC-pHILIC |
| Nonanoic acid | [M-H]- | 157.1233 | 1.40 | ZIC-pHILIC |
| Pimelic acid/Dimethylglutaric acid/Methyladipic acid | [M-H]- | 159.0662 | 7.88 | ZIC-pHILIC |
| Mono-methyl adipate | [M-H]- | 159.0662 | 2.22 | ZIC-pHILIC |
| Hydroxy-methylglutaric acid | [M-H]- | 161.0455 | 9.52 | ZIC-pHILIC |
| Decanoic acid | [M-H]- | 171.1390 | 1.37 | ZIC-pHILIC |
| Octanedioic acid | [M-H]- | 173.0818 | 7.55 | ZIC-pHILIC |
| Nonanedioic acid | [M-H]- | 187.0975 | 7.08 | ZIC-pHILIC |
| Hydroxydecanoic acid | [M-H]- | 187.1340 | 1.40 | ZIC-pHILIC |
| Octanedioic acid dimethyl | [M-H]- | 201.1133 | 1.43 | ZIC-pHILIC |
| Tridecanoic acid | [M-H]- | 213.1860 | 1.32 | ZIC-pHILIC |
| Hydroxydodecanoic acid | [M-H]- | 215.1653 | 1.35 | ZIC-pHILIC |
| dioxo-Dodecanoic acid | [M-H]- | 227.1291 | 1.43 | ZIC-pHILIC |
| Tetradecanoic acid | [M-H]- | 227.2018 | 1.31 | ZIC-pHILIC |
| Tetradecanedioic acid | [M-H]- | 257.1764 | 1.38 | ZIC-pHILIC |
| Hydroxyhexadecanoic acid | [M-H]- | 271.2280 | 1.29 | ZIC-pHILIC |
| Elaidic acid/Vaccenic acid | [M-H]- | 281.2486 | 1.27 | ZIC-pHILIC |
| Hexadecanedioic acid | [M-H]- | 285.2075 | 1.35 | ZIC-pHILIC |
| 13(S)-Hydroxyoctadeca-9Z,11E-dienoic acid | [M-H]- | 295.2278 | 1.30 | ZIC-pHILIC |
| Eicosapentaenoic acid | [M-H]- | 301.2173 | 1.27 | ZIC-pHILIC |
| Eicosatrienoic acid | [M-H]- | 305.2485 | 1.26 | ZIC-pHILIC |
| **Steroids and steroid derivatives** |  |  |  |  |
| Porphobilinogen | [M+H]+ | 210.0759 | 2.38 | Hypersil GOLD C18 |
| Medroxyprogesterone | [M-H]- | 343.2277 | 1.27 | ZIC-pHILIC |
| Androsterone sulphate/  Androstan-3ß-ol-16-one sulphate | [M-H]- | 369.1742 | 1.29 | ZIC-pHILIC |
| Deoxycholic acid/Ursodeoxycholic acid | [M-H]- | 391.2859 | 1.46 | ZIC-pHILIC |
| Glycodeoxycholate/Glycochenodeoxycholic acid/Glycoursodeoxycholic acid | [M-H]- | 448.3072 | 1.45 | ZIC-pHILIC |
| Glycocholic acid | [M+H]+ | 466.3151 | 8.29 | Hypersil GOLD C18 |
| Taurodeoxycholic acid/  Taurochenodeoxycholic acid | [M-H]- | 498.2898 | 1.39 | ZIC-pHILIC |
| Taurocholic acid | [M-H]- | 514.2846 | 1.46 | ZIC-pHILIC |
| **Phenylpropanoids** |  |  |  |  |
| Phenylpropanoate | [M-H]- | 149.0607 | 1.59 | ZIC-pHILIC |
| Phenyllactic acid/Hydroxyphenyl propionic acid | [M-H]- | 165.0556 | 2.53 | ZIC-pHILIC |
| Apigenin/Genistein | [M+H]+ | 271.0623 | 8.34 | Hypersil GOLD C18 |
| **Other lipids and lipid-like molecules** |  |  |  |  |
| Aminocyclohexanecarboxylic acid | [M+H]+ | 144.1020 | 0.97 | Hypersil GOLD C18 |
| Pinene-oxide | [M-H]- | 197.1183 | 1.42 | ZICpHILIC |
| Jasmonic acid | [M-H]- | 209.1183 | 1.4 | ZICpHILIC |
| gamma-Linolenic acid | [M-H]- | 323.2232 | 1.32 | ZICpHILIC |
| Prostaglandins A1/B1/E1 | [M-H]- | 335.2230 | 1.31 | ZICpHILIC |
| Prostaglandin F1 alpha | [M-H]- | 355.2489 | 1.35 | ZICpHILIC |
| Psychosine | [M-H]- | 460.3281 | 1.36 | ZICpHILIC |
| **Organic nitrogen compounds** |  |  |  |  |
| Putrescine | [M+H]+ | 89.1076 | 0.81 | Hypersil GOLD C18 |
| N-Acetylputrescine | [M+H]+ | 131.1181 | 0.81 | Hypersil GOLD C18 |
| Spermidine | [M+H]+ | 146.1653 | 0.79 | Hypersil GOLD C18 |
| N8-Acetylspermidine | [M+H]+ | 188.1760 | 0.89 | Hypersil GOLD C18 |
| erythro-Dihydrosphingosine | [M-H]- | 283.2642 | 1.27 | ZIC-pHILIC |

RT, Retention time (minutes).

* Methyl-oxovaleric acids refers to several isomers: 4-Methyl-2-oxovaleric acid, 3-Methyl-2-oxovaleric acid, 2-Ketohexanoic acid.

† Pentoses phosphate refer to several isomers that cannot be distinguished using LC-MS: Ribose 5-phosphate, Ribose 1-phosphate, Ribulose 5-phosphate, Xylulose 5-phosphate.

‡ Disaccharides refer to several carbohydrates that cannot be distinguished using LC-MS: Sucrose, Maltose, Isomaltose, Lactulose, Melibiose, Trehalose, Lactose.

§ Dimethyluric acids refers to several isomers that cannot be distinguished using only LC-MS: 4-Methyl-2-oxovaleric acid, 3-Methyl-2-oxovaleric acid, 2-Ketohexanoic acid.

## eTable 2. List of the metabolites of interest validated by fragmentation.

| **Metabolite** | **Ion Detected** | **m/z** | **RT (min)** | **Identification Status** |
| --- | --- | --- | --- | --- |
| Putrescine | [M+H]+ | 89.1076 | 0.81 | a, c, e |
| Spermidine | [M+H]+ | 146.1653 | 0.79 | a, c, e |
| N-Acetyl-Cystein | [M+H]+ | 164.0379 | 1.84 | a, c, e |
| N-Acetylspermidine | [M+H]+ | 188.1760 | 0.89 | a, c, e |
| Hydroxytryptophan | [M+H]+ | 221.0925 | 1.74 | a, c, e |
| N-Acetylputrescine | [M+H]+ | 131.1181 | 0.81 | a, c, e |
| Pyridoxal | [M+H]+ | 168.0656 | 1.07 | a, c, e |
| Glutathione | [M+H]+ | 308.0913 | 1.17 | a, c, e |
| Lactic acid | [M-H]- | 89.0243 | 4.38 | a, b, d |
| Malonic acid | [M-H]- | 103.0036 | 9.36 | a, b, d |
| Uracil | [M-H]- | 111.0200 | 2.97 | a, b, d |
| Fumaric acid/maleic acid | [M-H]- | 115.0036 | 9.37 | a, b, d |
| Oxovaleric acid | [M-H]- | 115.0401 | 2.13 | a, b, d |
| Valine | [M-H]- | 116.0717 | 5.17 | a, b |
| Nicotinamide | [M-H]- | 121.0407 | 2.22 | a, b |
| Thymine | [M-H]- | 125.0356 | 2.35 | a, b, d |
| Isoleucine | [M-H]- | 130.0873 | 4.06 | a, b, d |
| Leucine | [M-H]- | 130.0873 | 3.59 | a, b, d |
| Malic acid | [M-H]- | 133.0142 | 9.41 | a, b, d |
| Hypoxanthine | [M-H]- | 135.0312 | 4.65 | a, b, d |
| Octanoic acid | [M-H]- | 143.1077 | 1.45 | a, b |
| Glutamic acid | [M-H]- | 146.0458 | 8.88 | a, b, d |
| Xanthine | [M-H]- | 151.0261 | 6.20 | a, b, d |
| Pyridoxine | [M-H]- | 168.0665 | 3.79 | a, b, d |
| Decanoic acid | [M-H]- | 171.1390 | 1.37 | a, b, d |
| Myo-inositol | [M-H]- | 179.0561 | 9.55 | a, b, d |
| Normetanephrine | [M-H]- | 182.0821 | 2.53 | a, b |
| Hydroxydecanoic acid | [M-H]- | 187.1340 | 1.40 | a, b |
| Kynurenine | [M-H]- | 207.0775 | 3.28 | a, b, d |
| Hydroxydodecanoic acid | [M-H]- | 215.1653 | 1.35 | a, b, d |
| Tetradecanedioic acid | [M-H]- | 257.1764 | 1.38 | a, b, d |
| Hexose phosphate * | [M-H]- | 259.0225 | 9.76 | a, b, d |
| Hexadecanedioic acid | [M-H]- | 285.2075 | 1.35 | a, b, d |
| Adenosine 5'-diphosphate | [M-H]- | 426.0223 | 8.93 | a, b, d |
| Isobutyric acid | [M-H]- | 87.0451 | 2.68 | a, b, d |
| Alanine | [M-H]- | 88.0404 | 7.61 | a, b, d |
| Creatine | [M-H]- | 130.0621 | 7.91 | a, b, d |
| Aminopyridine-carboxylic acid | [M-H]- | 137.0356 | 2.59 | a, b, d |
| Glutamine | [M-H]- | 145.0617 | 7.86 | a, b, d |
| Histidine | [M-H]- | 154.0621 | 7.46 | a, b, d |
| Glycerol phosphate | [M-H]- | 171.0064 | 8.17 | a, b, d |
| Pyridoxic acid | [M-H]- | 182.0459 | 1.62 | a, b, d |
| Inosine-monophosphate | [M-H]- | 347.0398 | 9.73 | a, b, d |
| Glutathione oxidized | [M-H]- | 611.1444 | 10.23 | a, b, d |
| Methyl-oxovaleric acids † | [M-H]- | 129.0558 | 1.62 | a, b, d |
| Nonanoic acid | [M-H]- | 157.1233 | 1.40 | a, b |
| Phenylpyruvic acid | [M-H]- | 163.0401 | 1.61 | a, b, d |
| Citric acid/Isocitric acid | [M-H]- | 191.0197 | 10.46 | a, b, d |
| Octanedioic acid dimethyl | [M-H]- | 201.1133 | 1.43 | a, b, d |
| Tridecanoic acid | [M-H]- | 213.1860 | 1.32 | a, b, d |
| Adenosine monophosphate | [M-H]- | 346.0558 | 8.26 | a, b, d |
| Purine | [M-H]- | 119.0360 | 2.49 | a, b |
| Nitrocatechol | [M-H]- | 154.0147 | 2.74 | a, b |
| Phosphoenolpyruvic acid | [M-H]- | 166.9751 | 10.17 | a, b, d |
| Adenosine triphosphate | [M-H]- | 505.9880 | 9.43 | a, b, f |
| Guanosine triphosphate | [M-H]- | 521.9835 | 11.12 | a, b, f |

Identification Status:

a, Based on accurate mass;

b, Based on ZIC-pHILIC column retention time similarity with a standard;

c, Based on C18 column retention time similarity with a standard;

d, Based on MS² spectrum (negative electrospray ionization) similarity with a standard;

e, Based on MS² spectrum (positive electrospray ionization) similarity with a standard;

f, Based on MS² spectrum (negative electrospray ionization) similarity with Metlin experimental MS/MS

RT, Retention time (minutes).

MS² spectrum refers to the fragmentation spectrum of the metabolites after ionization.

* Hexose phosphate refers to several isomers: Glucose 1-phosphate, Mannose 1-phosphate, Fructose 1-phosphate, Mannose 6-phosphate, Fructose 6-phosphate, Galactose 1-phosphate.

† Methyl-oxovaleric acids refers to several isomers that cannot be distinguish using only LC-MS: 4-Methyl-2-oxovaleric acid, 3-Methyl-2-oxovaleric acid, 2-Ketohexanoic acid.

## eTable 3. Fiber typing using proteomics on whole muscle samples.

|  |  | **Quantification (arbitrary unit) Mean (sd)** | | |  | |  |
| --- | --- | --- | --- | --- | --- | --- | --- |
| **Protein** | **Fiber type** | **Septic**  **Shock**  **(N=12)** | **Cardiogenic shock**  **(N=9)** | **Brain dead**  **(N=10)** | | **Raw**  **p-value** | |
| Myosin-1 (MYH1) | IIX (Fast glycolytic fibers) | 34.5 (1.4) | 35.7 (1.4) | 35.7 (1.7) | | 0.07 | |
| Myosin-2A  (MYH2A) | IIA (Fast oxidative fibers) | 38.7 (1.0) | 38.3 (0.9) | 38.4 (1.0) | | 0.25 | |
| Myosin-7B  (MYH7B) | I (Slow oxidative fibers) | 24.9 (1.5) | 25.3 (1.2) | 25.1 (1.2) | | 0.74 | |
| Troponin C,  fast SM | Fast fibers | 32.9 (1.2) | 32.8 (1.0) | 33.1 (1.2) | | 0.88 | |
| Troponin C, slow SM | Slow fibers | 32.9 (1.3) | 33.2 (1.2) | 33.8 (0.9) | | 0.15 | |
| Troponin I,  fast SM | Fast fibers | 33.4 (1.3) | 33.6 (0.9) | 33.4 (1.0) | | 0.73 | |
| Troponin I,  slow SM | Slow fibers | 33.5 (1.2) | 34.2 (1.0) | 34.6 (0.9) | | 0.02 | |
| Troponin T,  fast SM | Fast fibers | 34.8 (1.1) | 34.9 (0.7) | 34.8 (0.9) | | 0.86 | |
| Troponin T, slow SM | Slow fibers | 34.0 (1.0) | 34.4 (0.8) | 34.5 (0.8) | | 0.19 | |
| Tropomyosin alpha-1 chain | Fast fibers | 36.5 (1.0) | 36.6 (0.9) | 36.7 (0.6) | | 0.93 | |
| Tropomyosin alpha-3 chain | Slow fibers | 34.9 (1.0) | 35.2 (0.9) | 35.5 (0.7) | | 0.29 | |

The p-values were computed using an analysis of variance test on the log-transformed data. The raw p-values only are presented as the assumption on the independence of tests is not satisfied; adjusted p-values could only be higher.

SM, skeletal muscle; S, septic shock group; C, cardiogenic shock group; BD, brain dead group; vs, versus.

## eTable 4. Tissue-associated proteins potentially responsible for contamination.

| **Protein** | **Tissue** | **Number of samples** | **Quantity*** | **Actin-α**  **Ratio** |
| --- | --- | --- | --- | --- |
| CP | Serum | 24/31 | 25.4 (2.2) | 2^13^ |
| RBP4 | Serum | 11/31 | 23.6 (1.9) | 2^15^ |
| CRP | Serum | 12/31 | 26.2 (1.3) | 2^12^ |
| MBP | Schwann cells | 6/31 | 26,0 (2,1) | 2^12^ |
| MPZ | Schwann cells | 10/31 | 26,5 (2,7) | 2^12^ |
| PMP2 | Schwann cells | 3/31 | 24,6 (2,8) | 2^14^ |
| FABP4 | Adipocytes | 31/31 | 28.1 (1.6) | 2^10^ |
| LIPE | Adipocytes | 4/31 | 25.4 (0.8) | 2^13^ |

* Arbitrary log-transformed unit, mean (SD)

CP, Ceruloplasmin ; RBP4, Retinol binding protein 4 ; CRP, C-reactive protein ; MBP, Myelin basic protein ; MPZ, Myelin protein P0 ; PMP2, Myelin P2 protein ; LIPE, Lipase E (hormone sensitive lipase) ; FABP4, Fatty acid binding protein 4 (adipocytes).

## eTable 5. First twenty proteins contributing to the second principal component.

| **Protein** | **UniProtKB** | **Percent of variability explained** |
| --- | --- | --- |
| 2.4-dienoyl-CoA reductase, mitochondrial | Q16698 | 0.29 |
| Apoptosis-inducing factor 1, mitochondrial | E9PMA0 | 0.28 |
| NADH dehydrogenase 1 alpha subcomplex subunit 9, mitochondrial | Q16795 | 0.28 |
| ATP synthase subunit beta, mitochondrial | P06576 | 0.28 |
| Dihydrolipoyl dehydrogenase, mitochondrial | P09622 | 0.28 |
| NADH dehydrogenase iron-sulfur protein 3, mitochondrial | O75489 | 0.27 |
| Mitochondrial pyruvate carrier 2 | Q5R3B4 | 0.27 |
| Cytochrome c | G4XXL9 | 0.27 |
| ATP synthase subunit O, mitochondrial | P48047 | 0.27 |
| 2-oxoglutarate dehydrogenase, mitochondrial | Q02218 | 0.27 |
| Citrate synthase, mitochondrial | B4DJV2 | 0.27 |
| Mitochondrial 2-oxoglutarate/malate carrier protein | Q02978 | 0.27 |
| Isocitrate dehydrogenase subunit beta, mitochondrial | O43837 | 0.27 |
| Succinate dehydrogenase flavoprotein subunit, mitochondrial | P31040 | 0.26 |
| NADH dehydrogenase iron-sulfur protein 2, mitochondrial | O75306 | 0.26 |
| Very long-chain specific acyl-CoA dehydrogenase, mitochondrial | P49748 | 0.26 |
| Protein NipSnap homolog 2 | O75323 | 0.26 |
| Ubiquinone biosynthesis mono-oxygenase COQ6, mitochondrial | Q9Y2Z9 | 0.26 |
| Cytochrome b-c1 complex subunit 2, mitochondrial | P22695 | 0.26 |
| Succinyl-CoA ligase [ADP/GDP-forming] subunit alpha, mitochondrial | P53597 | 0.26 |

The proteins from the second dimension of the principal component analysis, that makes it possible to distinguish the three groups, are presented.

## eTable 6. First twenty metabolites contributing to the second principal component.

| **Metabolite** | **Percent of variability explained** |
| --- | --- |
| N-Acetylspermidine | 2.30 |
| Adenosine monophosphate (AMP) | 1.78 |
| Pentoses phosphate | 1.76 |
| Phosphonoacetic acid | 1.75 |
| Threo-Phenylserine | 1.59 |
| N-Isobutyrylglycine | 1.47 |
| Riboflavin monophosphate | 1.46 |
| Spermidine | 1.44 |
| alpha-Aminoadipic acid | 1.37 |
| UDP-galactose | 1.32 |
| Saccharic acid | 1.24 |
| Bisphosphoglycerate | 1.22 |
| Malate | 1.21 |
| Orotidine | 1.18 |
| Aminoimidazole-carboxamide ribotide | 1.17 |
| Adenosine diphosphate ADP | 1.16 |
| Aminopyridine-carboxylic acid | 1.09 |
| Inosine | 1.06 |
| N-Acetylputrescine | 1.04 |
| Uracil | 1.03 |

The metabolites from the second dimension of the principal component analysis, that makes it possible to distinguish the three groups, are presented.

## eTable 7. Proteins differentially abundant between groups.

| **Protein** | **Symbol** | **Log2FC**  **S vs C** | **Log2FC**  **S vs BD** | **Raw**  **p-value** | **Adjusted**  **p-value** |
| --- | --- | --- | --- | --- | --- |
| NADH dehydrogenase [ubiquinone] 1 alpha subcomplex subunit 10, mitochondrial | NDUFA10 | -1.78 | -1.8 | <0.001 | <0.001 |
| ES1 protein homolog, mitochondrial | GATD3A | -1.34 | -1.5 | <0.001 | <0.001 |
| Cytochrome c | CYCS | -1.45 | -1.74 | <0.001 | <0.001 |
| NAD-dependent protein deacetylase sirtuin-3, mitochondrial | SIRT3 | -4.21 | -1.67 | <0.001 | <0.001 |
| NADH dehydrogenase [ubiquinone] 1 alpha subcomplex subunit 2 | NDUFA2 | -1.35 | -1.59 | <0.001 | <0.001 |
| NADH dehydrogenase [ubiquinone] iron-sulfur protein 5 | NDUFS5 | -1.89 | -1.88 | <0.001 | <0.001 |
| Ubiquinone biosynthesis protein COQ9, mitochondrial | COQ9 | -1.17 | -1.27 | <0.001 | <0.001 |
| NADH dehydrogenase [ubiquinone] iron-sulfur protein 2, mitochondrial | NDUFS2 | -1.92 | -1.97 | <0.001 | <0.001 |
| NADH dehydrogenase [ubiquinone] iron-sulfur protein 3, mitochondrial | NDUFS3 | -1.86 | -1.93 | <0.001 | <0.001 |
| Apoptosis-inducing factor 1, mitochondrial | AIFM1 | -1.64 | -1.65 | <0.001 | <0.001 |
| Retinal dehydrogenase 1 | ALDH1A1 | -1.78 | -1.25 | <0.001 | <0.001 |
| NADH-ubiquinone oxidoreductase chain 5 | MT-ND5 | -4.58 | -3.95 | <0.001 | <0.001 |
| Fumarate hydratase, mitochondrial | FH | -1.56 | -1.38 | <0.001 | <0.001 |
| Dihydrolipoyl dehydrogenase, mitochondrial | DLD | -1.31 | -1.44 | <0.001 | <0.001 |
| Acetyltransferase component of pyruvate dehydrogenase complex | DLAT | -1.36 | -1.5 | <0.001 | <0.001 |
| Creatine kinase B-type | CKB | -5.27 | -4.04 | <0.001 | <0.001 |
| Electron transfer flavoprotein subunit alpha, mitochondrial | ETFA | -1.62 | -1.84 | <0.001 | <0.001 |
| Creatine kinase S-type, mitochondrial | CKMT2 | -1.77 | -1.75 | <0.001 | <0.001 |
| NADH dehydrogenase [ubiquinone] 1 beta subcomplex subunit 7 | NDUFB7 | -3.7 | -4.26 | <0.001 | <0.001 |
| Voltage-dependent anion-selective channel protein 1 | VDAC1 | -0.93 | -0.82 | <0.001 | <0.001 |
| ATP synthase subunit alpha, mitochondrial | ATP5F1A | -1.18 | -1.05 | <0.001 | <0.001 |
| NADH-ubiquinone oxidoreductase 75 kDa subunit, mitochondrial | NDUFS1 | -1.66 | -1.72 | <0.001 | <0.001 |
| Delta-1-pyrroline-5-carboxylate dehydrogenase, mitochondrial | ALDH4A1 | -1.69 | -1.71 | <0.001 | <0.001 |
| Thioredoxin-dependent peroxide reductase, mitochondrial | PRDX3 | -1.28 | -0.99 | <0.001 | <0.001 |
| ATP synthase subunit delta, mitochondrial | ATP5F1D | -1.76 | -1.79 | <0.001 | <0.001 |
| Peptidyl-prolyl cis-trans isomerase F, mitochondrial | PPIF | -3.42 | -3.34 | <0.001 | <0.001 |
| Succinate dehydrogenase [ubiquinone] flavoprotein subunit, mitochondrial | SDHA | -1.53 | -1.39 | <0.001 | <0.001 |
| Cytochrome b-c1 complex subunit 1, mitochondrial | UQCRC1 | -1.28 | -1.24 | <0.001 | <0.001 |
| 3-hydroxyisobutyrate dehydrogenase, mitochondrial | HIBADH | -1.41 | -1.9 | <0.001 | <0.001 |
| Dihydrolipoyllysine-residue succinyltransferase component of 2-oxoglutarate dehydrogenase complex, mitochondrial | DLST | -1.2 | -1.71 | <0.001 | <0.001 |
| Malate dehydrogenase, mitochondrial | MDH2 | -1.1 | -1.3 | <0.001 | <0.001 |
| Enoyl-CoA delta isomerase 1, mitochondrial | ECI1 | -1.65 | -1.86 | <0.001 | <0.001 |
| 3-ketoacyl-CoA thiolase, mitochondrial | ACAA2 | -1.45 | -1.82 | <0.001 | <0.001 |
| ATP synthase subunit O, mitochondrial | ATP5PO | -1.35 | -1.34 | <0.001 | <0.001 |
| Isocitrate dehydrogenase [NADP], mitochondrial | IDH2 | -1.56 | -1.75 | <0.001 | <0.001 |
| Succinate-semialdehyde dehydrogenase, mitochondrial | ALDH5A1 | -2.19 | -2.58 | <0.001 | <0.001 |
| Succinyl-CoA ligase [ADP/GDP-forming] subunit alpha, mitochondrial | SUCLG1 | -1.81 | -1.7 | <0.001 | <0.001 |
| 10 kDa heat shock protein, mitochondrial | HSPE1 | -0.71 | -1.39 | <0.001 | <0.001 |
| 2-oxoglutarate dehydrogenase, mitochondrial | OGDH | -1.81 | -2.09 | <0.001 | <0.001 |
| Methylmalonate-semialdehyde dehydrogenase [acylating], mitochondrial | ALDH6A1 | -3.59 | -3.52 | <0.001 | <0.001 |
| D-beta-hydroxybutyrate dehydrogenase, mitochondrial | BDH1 | -5.74 | -4.69 | <0.001 | <0.001 |
| Mitochondrial 2-oxoglutarate/malate carrier protein | SLC25A11 | -1.8 | -1.44 | <0.001 | <0.001 |
| NAD(P) transhydrogenase, mitochondrial | NNT | -2.07 | -2.17 | <0.001 | <0.001 |
| Mitochondrial import receptor subunit TOM20 homolog | TOMM20 | -2.95 | 0.26 | <0.001 | <0.001 |
| 2,4-dienoyl-CoA reductase, mitochondrial | DECR1 | -1.92 | -1.96 | <0.001 | <0.001 |
| NADH dehydrogenase [ubiquinone] 1 alpha subcomplex subunit 5 | NDUFA5 | -1.6 | -1.44 | <0.001 | <0.001 |
| Succinyl-CoA ligase [GDP-forming] subunit beta, mitochondrial | SUCLG2 | -3.53 | -3.26 | <0.001 | <0.001 |
| Aconitate hydratase, mitochondrial | ACO2 | -1.78 | -1.57 | <0.001 | <0.001 |
| Protein NipSnap homolog 3B | NIPSNAP3B | -4.74 | -5.02 | <0.001 | <0.001 |
| Acyl-coenzyme A thioesterase 13;Acyl-coenzyme A thioesterase 13, N-terminally processed | ACOT13 | -3.08 | -2.5 | <0.001 | <0.001 |
| NADH dehydrogenase [ubiquinone] 1 alpha subcomplex subunit 13 | NDUFA13 | -1.77 | -2.19 | <0.001 | <0.001 |
| Succinyl-CoA ligase [ADP-forming] subunit beta, mitochondrial | SUCLA2 | -2.99 | -3.18 | <0.001 | <0.001 |
| Acetyl-CoA acetyltransferase, mitochondrial | ACAT1 | -1.64 | -1.77 | <0.001 | <0.001 |
| ATP synthase F1 subunit beta, mitochondrial | ATP5F1B | -1.03 | -0.91 | <0.001 | <0.001 |
| Isocitrate dehydrogenase [NAD] subunit beta, mitochondrial | IDH3B | -2.85 | -2.4 | 0.001 | 0.028 |
| Citrate synthase;Citrate synthase, mitochondrial | CS | -1.84 | -2.17 | 0.001 | 0.028 |
| Flotillin-2 | FLOT2 | 1.33 | 4.54 | 0.001 | 0.028 |
| NADH dehydrogenase [ubiquinone] flavoprotein 2, mitochondrial | NDUFV2 | -1.5 | -1.72 | 0.001 | 0.028 |
| Adenylate kinase 2, mitochondrial | AK2 | -1.35 | -0.8 | 0.001 | 0.028 |
| NADH dehydrogenase [ubiquinone] flavoprotein 1, mitochondrial | NDUFV1 | -2.16 | -2.23 | 0.001 | 0.028 |
| 5-demethoxyubiquinone hydroxylase, mitochondrial | COQ7 | -2.86 | -3.66 | 0.001 | 0.028 |
| NADH dehydrogenase [ubiquinone] 1 beta subcomplex subunit 10 | NDUFB10 | -1.27 | -1.25 | 0.001 | 0.028 |
| Pyruvate dehydrogenase protein X component, mitochondrial | PDHX | -1.33 | -1.36 | 0.001 | 0.028 |
| Serum deprivation-response protein | CAVIN2 | -3.55 | -3.46 | 0.001 | 0.028 |
| Aspartate aminotransferase, mitochondrial | GOT2 | -1.28 | -1.43 | 0.001 | 0.028 |
| 60 kDa heat shock protein, mitochondrial | HSPD1 | -0.61 | -1.05 | 0.001 | 0.028 |
| Medium-chain specific acyl-CoA dehydrogenase, mitochondrial | ACADM | -3.03 | -2.69 | 0.001 | 0.028 |
| Cytochrome b-c1 complex subunit 2, mitochondrial | UQCRC2 | -1.24 | -1.3 | 0.001 | 0.028 |
| Enoyl-CoA hydratase, mitochondrial | ECHS1 | -1.29 | -1.12 | 0.001 | 0.028 |
| Hydroxymethylglutaryl-CoA lyase, mitochondrial | HMGCL | -3.75 | -3.67 | 0.001 | 0.028 |
| Electron transfer flavoprotein subunit beta | ETFB | -2.58 | -2.73 | 0.001 | 0.028 |
| Stress-70 protein, mitochondrial | HSPA9 | -0.83 | -1.12 | 0.001 | 0.028 |
| 28 kDa heat- and acid-stable phosphoprotein | PDAP1 | -4.14 | -3.19 | 0.001 | 0.028 |
| Electron transfer flavoprotein-ubiquinone oxidoreductase, mitochondrial | ETFDH | -3.66 | -3.82 | 0.001 | 0.028 |
| NADH dehydrogenase [ubiquinone] 1 alpha subcomplex subunit 9, mitochondrial | NDUFA9 | -1.9 | -2.05 | 0.001 | 0.028 |
| 2-methoxy-6-polyprenyl-1,4-benzoquinol methylase, mitochondrial | COQ5 | -2.58 | -3.01 | 0.001 | 0.028 |
| Acyl-coenzyme A thioesterase 1 | ACOT1 | -2.96 | -2.8 | 0.001 | 0.028 |
| Protein phosphatase PTC7 homolog | PPTC7 | 0.27 | -1.88 | 0.001 | 0.028 |
| Transmembrane protein 143 | TMEM143 | -3.25 | -3.55 | 0.001 | 0.028 |
| 3-hydroxyacyl-CoA dehydrogenase type-2 | HSD17B10 | -1.66 | -0.6 | 0.001 | 0.028 |
| Translational activator of cytochrome c oxidase 1 | TACO1 | -4.69 | -2.75 | 0.001 | 0.028 |
| Cat eye syndrome critical region protein 5 | HDHD5-AS1 | -4.52 | -3.16 | 0.001 | 0.028 |
| Transmembrane protein 126A | TMEM126A | -2.82 | -3.72 | 0.001 | 0.028 |
| L-2-hydroxyglutarate dehydrogenase, mitochondrial | L2HGDH | -3.58 | -3.25 | 0.001 | 0.028 |
| Ubiquinone biosynthesis monooxygenase COQ6, mitochondrial | COQ6 | -3.16 | -3.24 | 0.001 | 0.028 |
| NADH dehydrogenase [ubiquinone] 1 beta subcomplex subunit 9 | NDUFB9 | -1.57 | -1.65 | 0.001 | 0.028 |
| Neural cell adhesion molecule 1 | NCAM1 | 2.07 | 1.74 | 0.002 | 0.044 |
| Enoyl-CoA delta isomerase 2, mitochondrial | ECI2 | -2.7 | -3.24 | 0.002 | 0.044 |
| NADH dehydrogenase [ubiquinone] 1 alpha subcomplex subunit 6 | NDUFA6 | -3.9 | -3.29 | 0.002 | 0.044 |
| Probable ATP-dependent RNA helicase DDX17 | DDX17 | 0.92 | 3.2 | 0.002 | 0.044 |
| Calcium/calmodulin-dependent protein kinase type II subunit delta | CAMK2D | 2.16 | 1.7 | 0.002 | 0.044 |
| Protein phosphatase 1 regulatory subunit 27 | PPP1R27 | 3.97 | 2.21 | 0.002 | 0.044 |
| NADH dehydrogenase [ubiquinone] iron-sulfur protein 8, mitochondrial | NDUFS8 | -1.39 | -1.02 | 0.002 | 0.044 |
| Cytochrome b-c1 complex subunit 8 | UQCRQ | -2.24 | -2.38 | 0.002 | 0.044 |
| Perilipin-1 | PLIN1 | 4.15 | 5.77 | 0.002 | 0.044 |
| NADH dehydrogenase [ubiquinone] iron-sulfur protein 6, mitochondrial | NDUFS6 | -2.31 | -2.75 | 0.002 | 0.044 |
| NADH dehydrogenase [ubiquinone] 1 alpha subcomplex subunit 7 | NDUFA7 | -2.56 | -2.94 | 0.002 | 0.044 |
| L-lactate dehydrogenase B chain;L-lactate dehydrogenase | LDHB | -1.49 | -1.46 | 0.002 | 0.044 |
| Pyruvate dehydrogenase E1 component subunit beta, mitochondrial | PDHB | -1.72 | -1.43 | 0.002 | 0.044 |
| Succinate dehydrogenase [ubiquinone] iron-sulfur subunit, mitochondrial | SDHB | -1.57 | -1.05 | 0.002 | 0.044 |
| Dual specificity mitogen-activated protein kinase kinase 4 | MAP2K4 | 2.51 | 2.48 | 0.002 | 0.044 |
| Cytochrome b-c1 complex subunit 11 | UQCRFS1 | -0.94 | -1.15 | 0.002 | 0.044 |
| NADH dehydrogenase [ubiquinone] 1 alpha subcomplex subunit 8 | NDUFA8 | -1.02 | -1.37 | 0.002 | 0.044 |
| Thiosulfate sulfurtransferase | TST | -3.86 | -3.06 | 0.002 | 0.044 |
| Atypical kinase ADCK3, mitochondrial | COQ8A | -2.45 | -2.65 | 0.002 | 0.044 |
| Methylmalonyl-CoA epimerase, mitochondrial | MCEE | -4.25 | -2.51 | 0.002 | 0.044 |
| GDP-fucose protein O-fucosyltransferase 1 | POFUT1 | -2.57 | 0.22 | 0.002 | 0.044 |
| Mycophenolic acid acyl-glucuronide esterase, mitochondrial | ABHD10 | -3.58 | -3.32 | 0.002 | 0.044 |
| Cytochrome b-c1 complex subunit 9 | UQCR10 | -2.09 | -2.81 | 0.002 | 0.044 |
| Coiled-coil-helix-coiled-coil-helix domain-containing protein 2 | CHCHD2 | -3.28 | -2.92 | 0.002 | 0.044 |
| MICOS complex subunit MIC60 | IMMT | -1.07 | -0.93 | 0.002 | 0.044 |
| Annexin A1 | ANXA1 | 0.99 | 2.46 | 0.003 | 0.061 |
| Plastin-2 | LCP1 | 2.88 | 4.33 | 0.003 | 0.061 |
| Cytochrome c oxidase subunit 6B1 | COX6B1 | -1.33 | -1.35 | 0.003 | 0.061 |
| Isocitrate dehydrogenase [NAD] subunit alpha, mitochondrial | IDH3A | -1.59 | -1.34 | 0.003 | 0.061 |
| Tubulin beta-2A chain | TUBB2A | 4.11 | 3.53 | 0.003 | 0.061 |
| Histone H3.2 | H3C15 | 0.82 | 4.04 | 0.003 | 0.061 |
| Isoleucine--tRNA ligase, mitochondrial | IARS2 | -3.31 | -2.82 | 0.003 | 0.061 |
| NADH dehydrogenase [ubiquinone] 1 alpha subcomplex subunit 12 | NDUFA12 | -2.39 | -2.4 | 0.003 | 0.061 |
| Glycophorin-A | GYPA | -2.87 | 1.9 | 0.004 | 0.072 |
| Acyl-CoA synthetase family member 2, mitochondrial | ACSF2 | -3.28 | -2.89 | 0.004 | 0.072 |
| NADH dehydrogenase [ubiquinone] iron-sulfur protein 4, mitochondrial | NDUFS4 | -2.25 | -2.6 | 0.004 | 0.072 |
| Protein NipSnap homolog 2 | NIPSNAP2 | -1.63 | -1.78 | 0.004 | 0.072 |
| Decorin | DCN | -0.71 | 0.65 | 0.004 | 0.072 |
| Cytochrome c1, heme protein, mitochondrial | CYC1 | -1.23 | -1.08 | 0.004 | 0.072 |
| Ubiquitin carboxyl-terminal hydrolase isozyme L1 | UCHL1 | 3.09 | 3.47 | 0.004 | 0.072 |
| Mimecan | OGN | -0.21 | 1.52 | 0.004 | 0.072 |
| ATP synthase F(0) complex subunit B1, mitochondrial | ATP5PB | -1.24 | -1.23 | 0.004 | 0.072 |
| Very long-chain specific acyl-CoA dehydrogenase, mitochondrial | ACADVL | -1.33 | -1.16 | 0.004 | 0.072 |
| Trifunctional enzyme subunit beta, mitochondrial;3-ketoacyl-CoA thiolase | HADHB | -1.31 | -1.4 | 0.004 | 0.072 |
| Fibulin-2 | FBLN2 | -1.23 | 2.33 | 0.004 | 0.072 |
| Coiled-coil domain-containing protein 58 | CCDC58 | -2.95 | -3.74 | 0.004 | 0.072 |
| NLR family member X1 | NLRX1 | -2.65 | -3.53 | 0.004 | 0.072 |
| Hydroxyacid-oxoacid transhydrogenase, mitochondrial | ADHFE1 | -4.7 | -3.24 | 0.004 | 0.072 |
| Eukaryotic peptide chain release factor GTP-binding subunit ERF3B | GSPT2 | 2.2 | 2.16 | 0.004 | 0.072 |
| CD44 antigen | CD44 | 1.54 | 3.33 | 0.005 | 0.084 |
| Lon protease homolog, mitochondrial | LONP1 | -3.11 | -3.17 | 0.005 | 0.084 |
| ADP/ATP translocase 1 | SLC25A4 | -1.06 | -1.03 | 0.005 | 0.084 |
| Alanine aminotransferase 1 | GPT | -3 | -3.29 | 0.005 | 0.084 |
| TLong-chain enoyl-CoA hydratase | HADHA | -1.14 | -1.3 | 0.005 | 0.084 |
| ATP synthase subunit e, mitochondrial | ATP5ME | -1.93 | -1.66 | 0.005 | 0.084 |
| NAD(P)(+)-arginine ADP-ribosyltransferase | ART3 | -3.98 | -2.14 | 0.005 | 0.084 |
| Thioredoxin, mitochondrial | TXN2 | -3.41 | -4.27 | 0.005 | 0.084 |
| Histidine triad nucleotide-binding protein 2, mitochondrial | HINT2 | -1.36 | -1.32 | 0.005 | 0.084 |
| Cytochrome c oxidase assembly protein COX11, mitochondrial | COX11 | -2.85 | -0.34 | 0.005 | 0.084 |
| Ig kappa chain V-II region Cum | IGKV2-40 | 4.01 | 2.57 | 0.006 | 0.094 |
| NADH dehydrogenase [ubiquinone] 1 beta subcomplex subunit 3 | NDUFB3 | -1.95 | -1.98 | 0.006 | 0.094 |
| Calcium-binding mitochondrial carrier protein Aralar1 | SLC25A12 | -1.36 | -1.47 | 0.006 | 0.094 |
| Protein disulfide-isomerase A4 | PDIA4 | 2.36 | 3.33 | 0.006 | 0.094 |
| Short-chain specific acyl-CoA dehydrogenase, mitochondrial | ACADS | -2.12 | -2.45 | 0.006 | 0.094 |
| Carnitine O-palmitoyltransferase 2, mitochondrial | CPT2 | -3.95 | -2.26 | 0.006 | 0.094 |
| Sulfite oxidase, mitochondrial | SUOX | -3.97 | -1.9 | 0.006 | 0.094 |
| Leucine-rich repeat-containing protein 59 | LRRC59 | 2.55 | 3.82 | 0.006 | 0.094 |
| Up-regulated during skeletal muscle growth protein 5 | ATP5MD | -1.39 | -1.5 | 0.006 | 0.094 |
| EMILIN-1 | EMILIN1 | 1.68 | 3.88 | 0.006 | 0.094 |

The p-values were computed using an analysis of variance test on the log-transformed data. To account for multiple comparisons, the Benjamini-Hochberg procedure was performed to adjust the p-values. The false discovery rate was set at 0.1. Only metabolites with adjusted p-values <0.1 are presented.

LogFC, Log Fold Change.

## eTable 8. Metabolites differentially abundant between groups.

| **Metabolite** | **Log2FC**  **S vs C** | **Log2FC**  **S vs BD** | **Raw**  **p-value** | **Adjusted**  **p-value** |
| --- | --- | --- | --- | --- |
| Histidine | -0.05 | -2.58 | <0.001 | <0.001 |
| Fumaric acid/maleic acid (Fumarate/maleate) | -0.46 | -1.27 | <0.001 | <0.001 |
| Aspartylglycosamine | -0.51 | -2.08 | <0.001 | <0.001 |
| Octanedioic acid dimethyl | 1.08 | 1.84 | <0.001 | <0.001 |
| Malic acid (malate) | -0.29 | -1 | <0.001 | <0.001 |
| Quinaldic acid | -1.86 | 0.53 | <0.001 | <0.001 |
| Phenylpyruvic acid | -0.62 | 1.4 | <0.001 | <0.001 |
| Thymine | -0.73 | 1.07 | <0.001 | <0.001 |
| Mannitol/Dulcitol | -2.46 | -2.53 | <0.001 | 0.002 |
| beta-Alanine | -0.05 | -1.47 | <0.001 | 0.003 |
| Aminoimidazole carboxamide ribotide | -2.82 | -4.93 | <0.001 | 0.003 |
| Malonic acid (Malonate) | -0.04 | 1.6 | <0.001 | 0.003 |
| Quinolinic acid | 2.05 | 2.69 | <0.001 | 0.003 |
| Anhydro-sorbitol | -0.14 | -1.84 | <0.001 | 0.003 |
| Nonanoic acid | 0.45 | 1.36 | <0.001 | 0.003 |
| Isobutyric acid | -0.07 | -0.99 | <0.001 | 0.003 |
| Imidazolelactic acid | 0.42 | -2.68 | <0.001 | 0.003 |
| ATP | -3.02 | 5.36 | <0.001 | 0.003 |
| Decanoic acid | 0.76 | 1.18 | <0.001 | 0.004 |
| Inosine | -0.05 | -1.26 | <0.001 | 0.004 |
| Hexadecanedioic acid | 0.61 | 1.38 | <0.001 | 0.004 |
| N-Acetylspermidine | 3.35 | 2.81 | <0.001 | 0.004 |
| Ribose/Xylulose | 2.77 | 0.82 | <0.001 | 0.004 |
| Argininosuccinic acid | 0.73 | -0.96 | <0.001 | 0.004 |
| Saccharopine | 0.34 | -3.59 | <0.001 | 0.004 |
| Tridecanoic acid | 0.21 | 1.42 | <0.001 | 0.004 |
| Glutathione reduced | 0.51 | -2.47 | <0.001 | 0.004 |
| Nitrophenol | -0.59 | 0.98 | <0.001 | 0.004 |
| Cysteinylglycine | -1.25 | -4.54 | <0.001 | 0.004 |
| 4-Nitrocatechol | -1.17 | 0.39 | <0.001 | 0.004 |
| Methyl oxalate | -0.19 | 3.85 | <0.001 | 0.005 |
| Dimethyl oxalic acid | 1.03 | 0.81 | <0.001 | 0.005 |
| IMP | -2.9 | -6.18 | <0.001 | 0.006 |
| Phenylpropanoate | 0.29 | 1.02 | <0.001 | 0.006 |
| Glycerol phosphate | 0.62 | -1.5 | <0.001 | 0.007 |
| Uracil | 2.68 | 0.79 | 0.001 | 0.007 |
| Mandelate | -0.5 | 0.89 | 0.001 | 0.008 |
| Nicotinamide | 0.15 | -1.03 | 0.001 | 0.008 |
| Pyridoxal | 1.63 | 2.65 | 0.001 | 0.009 |

The p-values were computed using an analysis of variance test on the log-transformed data. To account for multiple comparisons, the Benjamini-Hochberg procedure was performed to adjust the p-values. The false discovery rate was set at 0.01. Only metabolites with adjusted p-values <0.01 are presented.

LogFC, Log Fold Change.

## eTable 9. Fatty acids accumulation in skeletal muscle from septic patients.

|  |  | **Log2FC**  **Mean Difference [95% CI]** | | |  | |
| --- | --- | --- | --- | --- | --- | --- |
| **Lipid** | **C*** | **Septic shock**  **vs Cardiogenic shock** | **Septic shock**  **vs brain dead** | **Raw p-value** | |  |
| Octanoic acid | 8 | +0.35 [-0.33; 1.03] | +0.77 [0.28; 1.26] | 0.002 | |  |
| Octanedioic acid | 8 | +1.18 [-0.07; 2.44] | +1.21 [0.31; 2.11] | 0.005 | |  |
| Octanedioic acid dimethyl | 8 | +1.08 [0.05; 2.10] | +1.84 [1.10; 2.58] | <0.001 | |  |
| Nonanoic acid | 9 | +0.45 [-0.55; 1.44] | +1.36 [0.65; 2.08] | <0.001 | |  |
| Hydroxydecanoic acid | 10 | +1.03 [0.28; 1.78] | +0.81 [0.27; 1.36] | <0.001 | |  |
| Decanoic acid | 10 | +0.76 [-0.13; 1.65] | +1.18 [0.54; 1.82] | <0.001 | |  |
| Hydroxydodecanoic acid | 12 | +1.01 [0.10; 1.93] | +0.07 [-0.59; 0.73] | 0.027 | |  |
| Tridecanoic acid | 13 | +0.21 [-0.91; 1.34] | +1.42 [0.60; 2.23] | <0.001 | |  |
| Tetradecanoic acid | 14 | 0.00 [-1.35; 1.36] | +0.95 [-0.02; 1.94] | 0.048 | |  |
| Tetradecanedioic acid | 14 | +0.24 [-0.98; 1.46] | +1.14 [0.25; 2.02] | 0.010 | |  |
| Hexadecanedioic acid | 16 | +0.61 [-0.43; 1.66] | +1.38 [0.63; 2.14] | <0.001 | |  |

The p-values were computed using an analysis of variance test on the log-transformed data. The raw p-values only are presented as the assumption on the independence of tests is not satisfied.

* Number of carbon atoms

Log2FC, Log2 fold change; 95% CI, 95% confidence interval.

## eTable 10. Enrichment analysis using the GAGE Algorithm, Septic shock versus Cardiogenic shock.

| **Kegg Pathway** | **Magnitude of change** | **Raw p-value** | **Adj. p-value** | **Number of proteins** |  |
| --- | --- | --- | --- | --- | --- |
| Oxidative phosphorylation | 0.51 | <0.001 | <0.001 | 93 | *** |
| Valine, leucine and isoleucine degradation | 0.55 | <0.001 | <0.001 | 34 | *** |
| Citrate cycle (TCA cycle) | 0.47 | <0.001 | <0.001 | 26 | *** |
| Propanoate metabolism | 0.50 | <0.001 | <0.001 | 22 | *** |
| Butanoate metabolism | 0.61 | <0.001 | 0.001 | 12 | ** |
| Fatty acid degradation | 0.40 | <0.001 | 0.002 | 27 | ** |
| Tryptophan metabolism | 0.45 | <0.001 | 0.006 | 18 | ** |
| Lysine degradation | 0.43 | 0.002 | 0.013 | 17 |  |
| Ketone body metabolism | 0.66 | 0.006 | 0.031 | 6 |  |
| Glyoxylate and dicarboxylate metabolism | 0.35 | 0.008 | 0.039 | 20 |  |
| Glycerolipid metabolism | 0.39 | 0.019 | 0.08 | 13 |  |
| Fatty acid elongation | 0.42 | 0.021 | 0.08 | 11 |  |
| Arginine and proline metabolism | 0.30 | 0.021 | 0.08 | 21 |  |
| Pyruvate metabolism | 0.25 | 0.029 | 0.10 | 30 |  |
| Sulfur metabolism | 0.50 | 0.032 | 0.11 | 7 |  |
| Ascorbate and aldarate metabolism | 0.53 | 0.035 | 0.11 | 6 |  |
| beta-Alanine metabolism | 0.34 | 0.043 | 0.13 | 14 |  |
| Arginine biosynthesis | 0.40 | 0.053 | 0.15 | 9 |  |
| Glycine, serine and threonine metabolism | 0.31 | 0.06 | 0.17 | 14 |  |
| Nicotinate and nicotinamide metabolism | 0.30 | 0.08 | 0.21 | 14 |  |

The missing values were imputed with the probabilistic minimum imputation method. Enrichment analysis was performed using functional class scoring (“gage” R Package), with the Kyoto encyclopedia of genes and genomes (KEGG) metabolic pathway database. To account for multiple comparisons, the p-values were adjusted using the Benjamini-Hochberg procedure, with a false discovery rate of 0.01. The pathway that are not considered differentially abundant (adjusted p-value > 0.01) are not shown. The table shows the first 20 KEGG pathways, ordered on adjusted p-values. The magnitude of change (without units) synthetize in one value the difference in the abundance of proteins that constitute a particular pathway: the greater the magnitude of change, the more differentially expressed the pathways are.

## eTable 11. Enrichment analysis using the GAGE Algorithm, Septic shock versus Brain Dead.

| **Kegg Pathway** | **Magnitude of change** | **Raw p-value** | **Adj. p-value** | **Number of proteins** |  |
| --- | --- | --- | --- | --- | --- |
| Oxidative phosphorylation | 0.46 | <0.001 | <0.001 | 93 | *** |
| Valine, leucine and isoleucine degradation | 0.42 | <0.001 | <0.001 | 34 | *** |
| Citrate cycle (TCA cycle) | 0.47 | <0.001 | <0.001 | 26 | *** |
| Propanoate metabolism | 0.43 | <0.001 | 0.004 | 22 | ** |
| Butanoate metabolism | 0.5 | 0.003 | 0.027 | 12 |  |
| Ketone body metabolism | 0.66 | 0.005 | 0.043 | 6 |  |
| Cysteine and methionine metabolism | 0.3 | 0.025 | 0.18 | 21 |  |
| Arginine biosynthesis | 0.44 | 0.030 | 0.19 | 9 |  |
| Alanine, aspartate & glutamate metabolism | 0.38 | 0.034 | 0.19 | 12 |  |
| Nicotinate and nicotinamide metabolism | 0.34 | 0.039 | 0.20 | 14 |  |
| Fatty acid degradation | 0.24 | 0.043 | 0.20 | 27 |  |
| Tryptophan metabolism | 0.26 | 0.08 | 0.36 | 18 |  |
| beta-Alanine metabolism | 0.29 | 0.20 | 0.38 | 14 |  |
| Lysine degradation | 0.24 | 0.13 | 0.49 | 17 |  |
| Steroid hormone biosynthesis | 0.34 | 0.20 | 0.67 | 7 |  |
| Fatty acid elongation | 0.27 | 0.21 | 0.68 | 11 |  |
| Glycine, serine and threonine metabolism | 0.23 | 0.23 | 0.69 | 14 |  |
| Pyruvate metabolism | 0.15 | 0.27 | 0.77 | 30 |  |
| Sulfur metabolism | 0.29 | 0.31 | 0.78 | 7 |  |
| Arachidonic acid metabolism | 0.22 | 0.31 | 0.78 | 12 |  |

The missing values were imputed with the probabilistic minimum imputation method. Enrichment analysis was performed using functional class scoring (“gage” R Package), with the Kyoto encyclopedia of genes and genomes (KEGG) pathway database. To account for multiple comparisons, the p-values were adjusted using the Benjamini-Hochberg procedure, with a false discovery rate of 0.01. The pathway that are not considered differentially abundant (adjusted p-value > 0.01) are not shown. The table shows the first 20 KEGG metabolic pathways, ordered on adjusted p-values. The magnitude of change (without units) synthetize in one value the difference in the abundance of proteins that constitute a particular pathway: the greater the magnitude of change, the more differentially expressed the pathways are.

## eTable 12. Enrichment analysis using the covariance analysis global test, missing values imputation with the maximum likelihood estimation algorithm.

|  | **Number of proteins** | |  |  |
| --- | --- | --- | --- | --- |
| **Pathways** | **In the pathway**  **(n)** | **Identified**  **(n, %)** | **Raw**  **P-value** | **Adj.**  **P-value** |
| **KEGG metabolic pathways** |  |  |  |  |
| beta-Alanine metabolism | 30 | 14 (46.7) | <0.001 | <0.001 |
| Citrate cycle (TCA cycle) | 30 | 26 (86.7) | <0.001 | 0.001 |
| Ketone body metabolism | 10 | 6 (60.0) | <0.001 | 0.001 |
| Arginine and proline metabolism | 50 | 21 (42.0) | <0.001 | 0.001 |
| Butanoate metabolism | 28 | 12 (42.9) | <0.001 | 0.001 |
| Fatty acid elongation | 27 | 11 (40.7) | <0.001 | 0.001 |
| Fatty acid degradation | 44 | 27 (61.4) | <0.001 | 0.001 |
| Val, Leu and Ile degradation | 48 | 34 (70.8) | <0.001 | 0.001 |
| Tryptophan metabolism | 42 | 18 (42.9) | <0.001 | 0.001 |
| Oxidative phosphorylation | 133 | 93 (69.9) | <0.001 | 0.001 |
| Pyruvate metabolism | 39 | 30 (76.9) | <0.001 | 0.001 |
| Propanoate metabolism | 34 | 22 (64.7) | <0.001 | 0.001 |
| Lysine degradation | 61 | 17 (27.9) | <0.001 | 0.002 |
| Phenylalanine metabolism | 16 | 6 (37.5) | <0.001 | 0.002 |
| Ala, Asp and Glu metabolism | 36 | 12 (33.3) | <0.001 | 0.002 |
| Fatty acid biosynthesis | 18 | 6 (33.3) | <0.001 | 0.002 |
| Ascorbate and aldarate metabolism | 27 | 6 (22.2) | <0.001 | 0.002 |
| Inositol phosphate metabolism | 73 | 10 (13.7) | <0.001 | 0.002 |
| Sulfur metabolism | 10 | 7 (70.0) | <0.001 | 0.003 |
| Cysteine and methionine metabolism | 49 | 21 (42.9) | 0.002 | 0.004 |
| Biosynthesis of unsaturated fatty acids | 27 | 8 (29.6) | 0.002 | 0.004 |
| Arginine biosynthesis | 21 | 9 (42.9) | 0.002 | 0.005 |
| Glyoxylate and dicarboxylate metabolism | 30 | 20 (66.7) | 0.004 | 0.010 |
| **KEGG signaling pathways** |  |  |  |  |
| Retrograde endocannabinoid signaling | 148 | 53 (35.8) | <0.001 | <0.001 |
| Peroxisome | 83 | 20 (24.1) | <0.001 | 0.007 |
| PPAR signalling pathway | 77 | 27 (35.1) | <0.001 | 0.007 |

The missing values were imputed with the maximum likelihood estimation algorithm. Enrichment analysis was performed using the covariance analysis global test on the the Kyoto encyclopedia of genes and genomes (KEGG) pathways database. To account for multiple comparisons, the p-values were adjusted using the Benjamini-Hochberg procedure, with a false discovery rate of 0.01. The pathway that are not considered differentially abundant (adjusted p-value > 0.01) are not shown. Ile, Isoleucine; Leu, Leucine; Val, Valine; Ala, Alanine; Asp, Aspartate; Glu, Glutamate; TCA, Tricyclic Acid; PPAR, Peroxisome proliferator-activated receptor.

## eTable 13. Enrichment analysis using the covariance analysis global test, missing values imputation with the structured least squares algorithm.

|  | **Number of proteins** | |  |  |
| --- | --- | --- | --- | --- |
| **Pathways** | **In the pathway**  **(n)** | **Identified**  **(n, %)** | **Raw**  **P-value** | **Adj.**  **P-value** |
| **KEGG metabolic pathways** |  |  |  |  |
| Citrate cycle (TCA cycle) | 30 | 26 (86.7) | <0.001 | <0.001 |
| Phenylalanine metabolism | 16 | 6 (37.5) | <0.001 | <0.001 |
| beta-Alanine metabolism | 30 | 14 (46.7) | <0.001 | <0.001 |
| Inositol phosphate metabolism | 73 | 10 (13.7) | <0.001 | <0.001 |
| Propanoate metabolism | 34 | 22 (64.7) | <0.001 | <0.001 |
| Arginine and proline metabolism | 50 | 21 (42.0) | <0.001 | <0.001 |
| Tryptophan metabolism | 42 | 18 (42.9) | <0.001 | <0.001 |
| Fatty acid elongation | 27 | 11 (40.7) | <0.001 | 0.001 |
| Valine, leucine and isoleucine degradation | 48 | 34 (70.8) | <0.001 | 0.001 |
| Oxidative phosphorylation | 133 | 93 (69.9) | <0.001 | 0.001 |
| Ala, Asp and Glu metabolism | 36 | 12 (33.3) | <0.001 | 0.001 |
| Glyoxylate and dicarboxylate metabolism | 30 | 20 (66.7) | <0.001 | 0.001 |
| Pyruvate metabolism | 39 | 30 (76.9) | <0.001 | 0.002 |
| Butanoate metabolism | 28 | 12 (42.9) | <0.001 | 0.002 |
| Fatty acid degradation | 44 | 27 (61.4) | <0.001 | 0.002 |
| Lysine degradation | 61 | 17 (27.9) | <0.001 | 0.002 |
| Histidine metabolism | 22 | 10 (45.5) | <0.001 | 0.002 |
| Biosynthesis of unsaturated fatty acids | 27 | 8 (29.6) | 0.001 | 0.004 |
| Glycine, serine and threonine metabolism | 40 | 14 (35.0) | 0.002 | 0.005 |
| Tyrosine metabolism | 35 | 13 (37.1) | 0.002 | 0.006 |
| Cysteine and methionine metabolism | 49 | 21 (42.9) | 0.002 | 0.006 |
| Ascorbate and aldarate metabolism | 27 | 6 (22.2) | 0.003 | 0.007 |
| **KEGG signaling pathways** |  |  |  |  |
| Retrograde endocannabinoid signaling | 148 | 53 (35.8) | <0.001 | 0.009 |
| cAMP signaling pathway | 216 | 38 (17.6) | <0.001 | 0.009 |
| PPAR signaling pathway | 77 | 27(35.1) | <0.001 | 0.009 |
| Peroxisome | 83 | 20 (24.1) | <0.001 | 0.009 |
| cGMP-PKG signaling pathway | 167 | 38 (22.8) | <0.001 | 0.010 |

The missing values were imputed with the Structured Least Squares Algorithm. Enrichment analysis was performed using the covariance analysis global test on the KEGG pathways database. To account for multiple comparisons, the p-values were adjusted using the Benjamini-Hochberg procedure, with a false discovery rate of 0.01. The pathway that are not considered differentially abundant (adjusted p-value > 0.01) are not shown. Ala, Alanine; Asp, Aspartate; Glu, Glutamate; TCA, Tricyclic Acid; cAMP, cyclic Adenosine monophosphate; cGMP-PKG, cyclic Guanosine monophosphate – Proteinase Kinase G; PPAR, Peroxisome proliferator-activated receptor.

## eTable 14. Enrichment analysis using the covariance analysis global test, complete case analysis (n=555 proteins).

|  | **Number of proteins** | |  |  |
| --- | --- | --- | --- | --- |
| **Pathways** | **In the pathway**  **(n)** | **Identified**  **(n, %)** | **Raw**  **P-value** | **Adj.**  **P-value** |
| **KEGG metabolic pathways** |  |  |  |  |
| Glyoxylate and dicarboxylate metabolism | 30 | 8 (26.7) | <0.001 | <0.001 |
| Citrate cycle (TCA cycle) | 30 | 16 (53.3) | <0.001 | 0.001 |
| Ala, Asp and Glu metabolism | 36 | 5 (13.9) | <0.001 | 0.002 |
| Oxidative phosphorylation | 133 | 41 (30.8) | <0.001 | 0.002 |
| Propanoate metabolism | 34 | 8 (23.5) | <0.001 | 0.002 |
| Arginine and proline metabolism | 50 | 9 (18) | <0.001 | 0.002 |
| Butanoate metabolism | 28 | 6 (21.4) | <0.001 | 0.002 |
| Tryptophan metabolism | 42 | 10 (23.8) | <0.001 | 0.002 |
| Val, Leu and Ile degradation | 48 | 12 (25) | <0.001 | 0.002 |
| Lysine degradation | 61 | 9 (14.8) | <0.001 | 0.002 |
| Fatty acid degradation | 44 | 15 (34.1) | <0.001 | 0.002 |
| Fatty acid elongation | 27 | 6 (22.2) | <0.001 | 0.002 |
| Pyruvate metabolism | 39 | 15 (38.5) | 0.001 | 0.003 |
| Cysteine and methionine metabolism | 49 | 6 (12.2) | 0.002 | 0.004 |
| beta-Alanine metabolism | 30 | 5 (16.7) | 0.005 | 0.009 |
| **KEGG signaling pathways** |  |  |  |  |
| Retrograde endocannabinoid signaling | 148 | 20 (13.5) | <0.001 | 0.004 |
| Apoptosis | 136 | 10 (7.4) | <0.001 | 0.004 |
| Necroptosis | 159 | 17 (10.7) | <0.001 | 0.007 |

Complete case analysis (n=555 proteins, proteins with at least one missing data were discarded). Enrichment analysis was performed using the covariance analysis global test on the KEGG pathways database. To account for multiple comparisons, the p-values were adjusted using the Benjamini-Hochberg procedure, with a false discovery rate of 0.01. The pathway that are not considered differentially abundant (adjusted p-value > 0.01) are not shown. Ile, Isoleucine; Leu, Leucine; Val, Valine; Ala, Alanine; Asp, Aspartate; Glu, Glutamate; TCA, Tricyclic Acid.TCA, Tricyclic Acid.

## eTable 15. Proteomics and metabolomics related to oxidative stress.

|  | **Log2FC**  **Mean Difference [95% CI]** | |  |
| --- | --- | --- | --- |
| **Analyte** | **Septic shock vs**  **cardiogenic shock** | **Septic shock vs**  **brain dead** | **Raw p-value** |
| **Proteomics** |  |  |  |
| Thioredoxin | 0.3 [-0.7; 1.3] | 0.3 [-0.7; 1.3] | 0.73 |
| Thiroredoxin reductase 1 | -2.9 [-6.6; 0.9] | -0.8 [-4.4; 2.8] | 0.18 |
| Thioredoxin 2, mitochondria | -3.4 [-6.7; -0.2] | -4.3 [-7.4; -1.1] | **0.005** |
| Thiroredoxin reductase 2, mitochondria | -2.2 [-4.6; 0.2] | -2.2 [-4.5; 0.1] | **0.037** |
| Peroxiredoxin I | 0.1 [-0.6; 0.9] | -0.2 [-0.9; 0.6] | 0.68 |
| Peroxiredoxin II | -0.1 [-1.2; 1.0] | 0.1 [-0.9; 1.2] | 0.88 |
| Peroxiredoxin III, mitochondria | -1.3 [-2.0; -0.6] | -1.0 [-1.7; -0.3] | **<0.001** |
| Peroxiredoxin IV | 2.6 [-0.3; 5.5] | 3.3 [0.5; 6.1] | **0.016** |
| Peroxiredoxin V, mitochondria & peroxisome | -0.2 [-0.8; 0.5] | -0.1 [-0.8; 0.5] | 0.79 |
| Glutathion disulfide synthetase | -1.0 [-4.0; 2.0] | -2.0 [-4.9; 0.9] | 0.26 |
| Glutathione reductase, mitochondria | -0.4 [-2.3; 1.6] | 0.3 [-1.6; 2.2] | 0.73 |
| Glutaredoxin | 1.3 [-1.3; 3.9] | 2.5 [-0.1; 4.3] | 0.07 |
| Glutaredoxin 3 | 0.2 [-3.0; 3.4] | 0.1 [-3.0; 3.1] | 0.99 |
| Glutaredoxin 5, mitochondria | -2.9 [-5.5; -0.3] | -2.9 [-5.4; -0.4] | **0.010** |
| Glutathione peroxidase 1 | -2.2 [-5.0; 0.7] | -1.5 [-4.2; 1.3] | 0.17 |
| Glutathione peroxidase 3 | 0.1 [-2.2; 2.4] | -0.8 [-3.0; 1.5] | 0.71 |
| Super oxide dismutase 1 | 2.1 [-0.9; 5.2] | 2.0 [-1.0; 5.0] | 0.15 |
| Super oxide dismutase 2, mitochondria | -0.7 [-1.9; 0.5] | -0.1 [-1.3; 1.1] | 0.38 |
| Catalase | -1.1 [-3.2; 1.1] | 0.6 [-1.5; 2.7] | 0.19 |
| **Metabolomics** |  |  |  |
| Glutathione (reduced) | 0.5 [-1.7; 2.7] | -2.5 [-4.0; -0.9] | **<0.001** |
| Glutathione (oxidized) | 0.6 [-1.3; 2.6] | -1.4 [-2.9; 0.0] | **0.017** |
| Spermidine | 1.2 [-0.04; 2.4] | 0.9 [0.01; 1.7] | **0.021** |
| N-Acetylspermidine | 3.4 [0.9; 5.8] | 2.8 [1.1; 4.7] | **<0.001** |
| Nicotinamide (vitamin PP) | 0.1 [-0.8; 1.1] | -1.0 [-0.3; -1.7] | **0.001** |
| ß-Nicotinamide mononucleotide | -0.8 [-3.4; 0.8] | -2.5 [-4.3; -0.6] | **0.009** |
| 4-Pyridoxic acid | 3.3 [0.4; 6.3] | 2.8 [0.7; 5.0] | **0.003** |

The p-values were computed using an analysis of variance test on the log-transformed data. The raw p-values are presented as the assumption on the independence of tests is not satisfied. One can deduce the significance of the comparison between two groups with the 95% confidence interval (95%CI) of the log fold change (LogFC).

LogFC, Log fold change; 95%CI, 95% confidence interval; S, septic shock group; C, cardiogenic shock group; BD, brain dead group.

# SUPPLEMENTAL FIGURES

## Figure S1. Quality check of the proteomics dataset.

A. The barplots represent the number of missing values in each samples (over a total of n=3346 proteins identified among the n=51 patients with proteomic samples).The number displayed in the bars represent missing values in each sample. The higher the bar, the more values are missing. The samples with more than 70% of missing value were excluded (n=8 exclusions). Four more patients were excluded due to missing informed consent.

B. Correlation matrix between each pair of samples (n=51), computed using all complete pairs of observations in these samples. Five samples are poorly correlated to the other ones, of which three with already more than 70% of missing value. Four more patients were excluded due to missing informed consent.

C. Correlation matrix between each pair of samples after the exclusion of samples with missing values and a low level of correlation with other replicates (n=10) and patients excluded due to missing informed consent (n=4). Thirty-seven samples are further processed.

D, E and F. Principal component analysis of the sixty more abundant proteins in the 37 seven samples from the figure S1C. Every sample is represented in the principal components (PC) 1 to 4, samples that are close together tend to have similar characteristics. Six outliers can be spotted in PC2, PC3 and PC4 and were excluded.

## Figure S2. Pre-processing of the proteomics dataset.

The barplots represent the distribution of the quantity of proteins among the groups of patients after log2 transformation (A), normalization by median centering (B) and missing data imputation (C). The boxplots of the same distribution are shown before (D) and after (E) median centering. D and E: the box plots represent the distribution of the quantity of proteins, the median being the bold horizontal line, the interquartile range being the box. The upper (lower) moustache extends from the upper (lower) edge to the highest (lowest) value at 1.5 times the interquartile space. The dots represent the outlier values above or below 1.5 times the interquartile space.

## Figure S3. Pre-processing of the metabolomics dataset.

The histograms represent the distribution of the quantity of metabolites among the groups of patients after log2 transformation (A), normalization by median centering (B) and missing data imputation (C). The boxplots of the same distribution are shown before (D) and after (E) median centering. D and E: the box plots represent the distribution of the quantity of proteins, the median being the bold horizontal line, the interquartile range being the box. The upper (lower) moustache extends from the upper (lower) edge to the highest (lowest) value at 1.5 times the interquartile space. The dots represent the outlier values above or below 1.5 times the interquartile space.

## Figure S4. Distribution of the quantity of proteins after imputation.

The barplots represent the distribution of the quantity of proteins among the groups of patients after log2 transformation, normalization by median centering and missing data imputation. (A) Imputation using the probabilistic minimum imputation which imputes with small values and leads to a bimodal distribution. Imputation with close neighborhood of measured values within a condition that do not distort the initial data distributions using (B) the maximum likelihood estimation (MLE) algorithm or (C) the structured least squares algorithm (SLSA).

## Figure S5. Scatterplot representing the imputed mean value depending the measured mean value for each protein.

Each point represents a protein depending on the imputed mean value as a function of the measured mean value. The value represented is the intensity after median centering and log2 transformation. Proteins for which no values were missing in the samples (and which were not imputed) are not shown. Correlation between imputed and measured values was investigated using linear regression, represented by the blue line and its 95% confidence interval (in gray).

(A) Imputation using the probabilistic minimum imputation which imputes with small values and leads to an absence of correlation between measured and imputed values. Imputation with close neighborhood of measured values within a condition using (B) the maximum likelihood estimation (MLE) algorithm or (C) the structured least squares algorithm (SLSA). Using the latter two, there is a strong correlation between measured and imputed values.

## Figure S6. Principal component analysis of the proteomics and metabolomics datasets stratified by sex, age and severity score.

The first two dimensions are shown, defining the subspace maximizing the variance of the dataset. Every point represents an individual sample (muscular proteome or metabolome). The individuals are color-coded according to the sex (A and B), the age (C and D), or the SAPS2 (Simplified Acute Physiology Score 2) severity score (E and F). Points that are close together tend to have similar proteome/metabolome. Large points and ellipses represent respectively the barycenter of each group and its 95% confidence interval. This two-dimensional subspace did not allow distinguishing a clear pattern regarding the sex, the age, or the SAPS2 severity score. Dim indicates Dimension.

## Figures S7 to S12. Representation of the proteome and the metabolome on the significantly altered KEGG pathways.

The following graphs correspond to the pathways as defined in the KEGG database. They were plotted using the "pathview" R package. The enzymes are represented by rectangles; the metabolites are represented by circles. The rectangles and the circles are separated in their center, and colored according to a scale corresponding to the Log2 Fold Change of the average difference between the two comparisons, group S versus C (left color), and group S versus BD (right color). Enzymes/metabolites in white are those not identified by mass spectrometry, either absent from the samples, or below the detection limit, or absent from the database (especially concerning metabolomics).

S, Septic shock group; C, Cardiogenic shock group; BD, Brain Dead group; Log2FC, Log2 fold change.

**Figure S7. Oxidative phosphorylation metabolic pathway.**

**Figure S8. Catabolism of branched-chain amino acids metabolic pathway.**

**Figure S9. Tricarboxylic acid cycle (Krebs cycle) metabolic pathway.**

**Figure S10. Ketone body metabolism metabolic pathway.**

**Figure S11. Fatty acids degradation metabolic pathway.**

**Figure S12. Peroxisome proliferator-activated receptors signaling pathway.**

## Figure S13. Pairwise enrichment analysis using the GAGE algorithm.

Generated using the proteomics dataset. Each data point represents a biological pathway in the Septic shock group compared respectively to the Cardiogenic shock group (A) or the Brain Dead group (B), depending on its significance (adjusted p-value, y-axis, logarithmic scale) and its magnitude of change (x-axis). The size of the point represents the number of proteins involved in a given pathway. Adjusted p-values were obtained after correction of the p-values by the Benjamini-Hochberg procedure. The magnitude of change (without units) synthetize in one value the difference in the abundance of proteins that constitute a particular pathway: the greater the magnitude of change, the more differentially expressed the pathways are, and the dot will move rightward on the x-axis. The dotted line represents the threshold for adjusted p-value significance (<0.01). Pathways with raw p-values <0.05 are annotated.

## Figure S14. Heat map of the oxidative phosphorylation pathway (proteomics dataset).

Each column represents a patient; each row a protein. The overlying dendrogram is a graphical representation of patient similarity assessed by the Euclidean distance: patients in the same cluster are more similar than patients in two separate clusters. Proteins in grey are the proteins that were not identified in the dataset. The relative intensities were scaled by rows. The heat map shows a global decrease of the proteins involved in the oxidative phosphorylation in the septic shock group.

Some samples were discarded due to quality checks. N=31 patients analyzed for the proteomics (Septic shock n=12, cardiogenic shock n=9, brain dead n=10).

REFERENCES

1. Singer M, Deutschman CS, Seymour C, et al (2016) The third international consensus definitions for sepsis and septic shock (sepsis-3). JAMA 315:801–810. https://doi.org/10.1001/jama.2016.0287

2. Rappsilber J, Mann M, Ishihama Y (2007) Protocol for micro-purification, enrichment, pre-fractionation and storage of peptides for proteomics using StageTips. Nat Protoc 2:1896–1906. https://doi.org/10.1038/nprot.2007.261

3. Cox J, Mann M (2008) MaxQuant enables high peptide identification rates, individualized p.p.b.-range mass accuracies and proteome-wide protein quantification. Nat Biotechnol 26:1367–1372. https://doi.org/10.1038/nbt.1511

4. (2019) UniProt: a worldwide hub of protein knowledge. Nucleic Acids Res 47:D506–D515. https://doi.org/10.1093/nar/gky1049

5. Giacomoni F, Le Corguillé G, Monsoor M, et al (2015) Workflow4Metabolomics: a collaborative research infrastructure for computational metabolomics. Bioinformatics 31:1493–1495. https://doi.org/10.1093/bioinformatics/btu813

6. Boudah S, Olivier M-F, Aros-Calt S, et al (2014) Annotation of the human serum metabolome by coupling three liquid chromatography methods to high-resolution mass spectrometry. J Chromatogr B Analyt Technol Biomed Life Sci 966:34–47. https://doi.org/10.1016/j.jchromb.2014.04.025

7. Sumner LW, Amberg A, Barrett D, et al (2007) Proposed minimum reporting standards for chemical analysis Chemical Analysis Working Group (CAWG) Metabolomics Standards Initiative (MSI). Metabolomics Off J Metabolomic Soc 3:211–221. https://doi.org/10.1007/s11306-007-0082-2

8. Lazar C, Gatto L, Ferro M, et al (2016) Accounting for the Multiple Natures of Missing Values in Label-Free Quantitative Proteomics Data Sets to Compare Imputation Strategies. J Proteome Res 15:1116–1125. https://doi.org/10.1021/acs.jproteome.5b00981

9. Karpievitch YV, Dabney AR, Smith RD (2012) Normalization and missing value imputation for label-free LC-MS analysis. BMC Bioinformatics 13 Suppl 16:S5. https://doi.org/10.1186/1471-2105-13-S16-S5

10. Schafer JL (1997) Analysis of Incomplete Multivariate Data, 1st ed. Chapman and Hall/CRC, London, UK

11. Bø TH, Dysvik B, Jonassen I (2004) LSimpute: accurate estimation of missing values in microarray data with least squares methods. Nucleic Acids Res 32:e34. https://doi.org/10.1093/nar/gnh026

12. Kanehisa M, Goto S (2000) KEGG: Kyoto Encyclopedia of Genes and Genomes. Nucleic Acids Res 28:27–30. https://doi.org/10.1093/nar/28.1.27

13. Kanehisa M, Sato Y, Furumichi M, et al (2019) New approach for understanding genome variations in KEGG. Nucleic Acids Res 47:D590–D595. https://doi.org/10.1093/nar/gky962

14. Mansmann U, Meister R (2005) Testing differential gene expression in functional groups. Goeman’s global test versus an ANCOVA approach. Methods Inf Med 44:449–453

15. Hummel M, Meister R, Mansmann U (2008) GlobalANCOVA: exploration and assessment of gene group effects. Bioinformatics 24:78–85. https://doi.org/10.1093/bioinformatics/btm531

16. Luo W, Friedman MS, Shedden K, et al (2009) GAGE: generally applicable gene set enrichment for pathway analysis. BMC Bioinformatics 10:161. https://doi.org/10.1186/1471-2105-10-161

17. Maleki F, Ovens KL, Hogan DJ, et al (2019) Measuring consistency among gene set analysis methods: A systematic study. J Bioinform Comput Biol 17:1940010. https://doi.org/10.1142/S0219720019400109

18. Benjamini Y, Hochberg Y (1995) Controlling the False Discovery Rate: A Practical and Powerful Approach to Multiple Testing. J R Stat Soc Ser B Methodol 57:289–300. https://doi.org/10.1111/j.2517-6161.1995.tb02031.x
