# Supplementary figures and images for: Using a multiomics approach to unravel a septic shock specific signature in skeletal muscle
Source: Sci Rep. 2022 Nov 5;12:18776. doi: 10.1038/s41598-022-23544-8 (PMC9637214; doi:10.1038/s41598-022-23544-8)

A

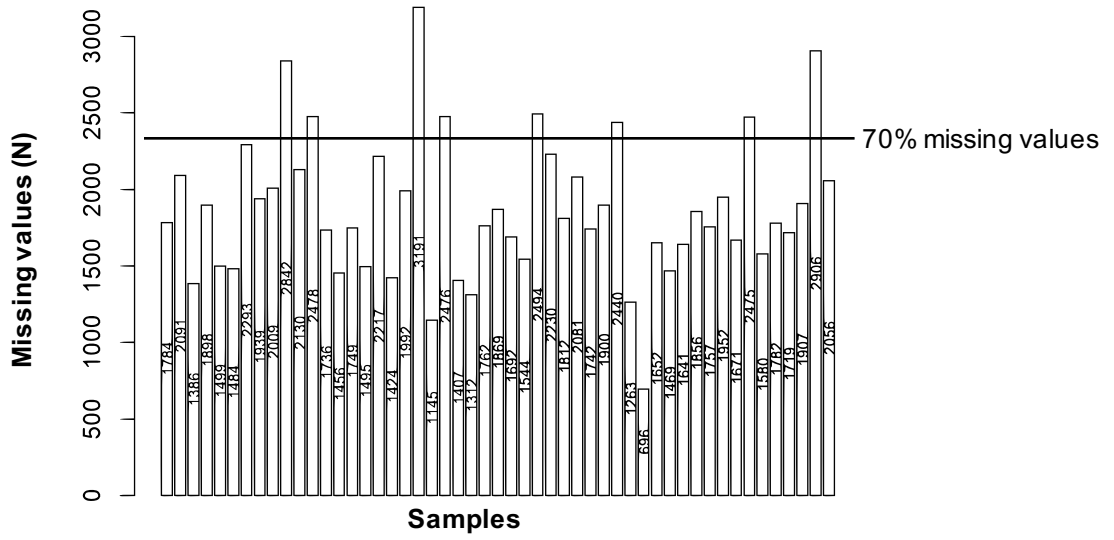

B

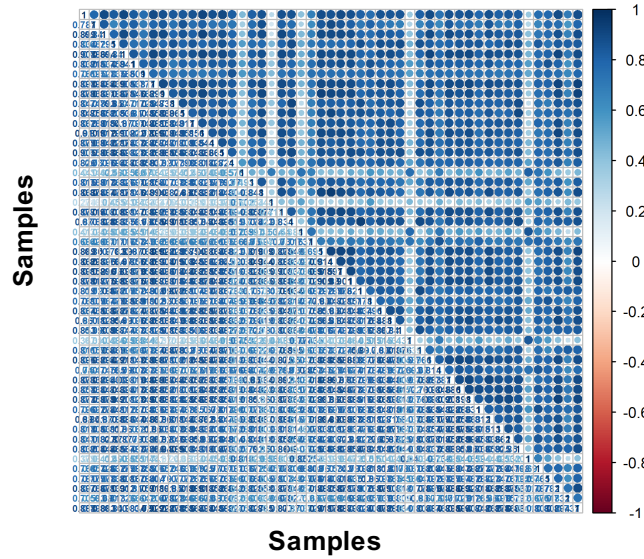

C

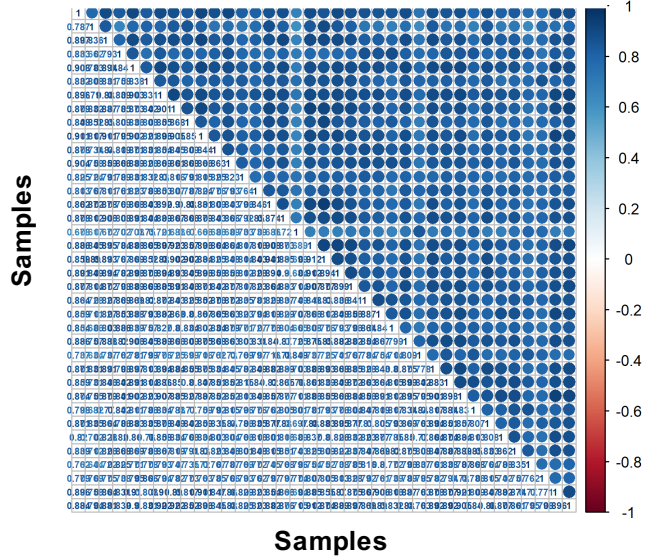

D

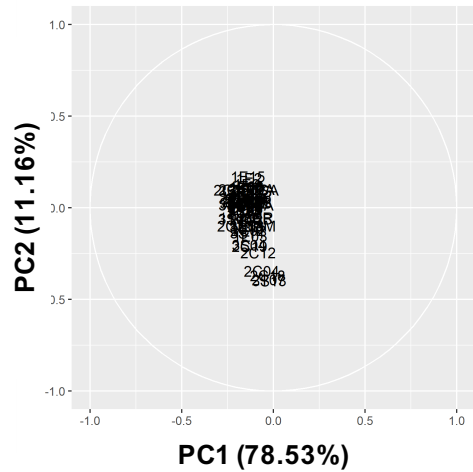

E

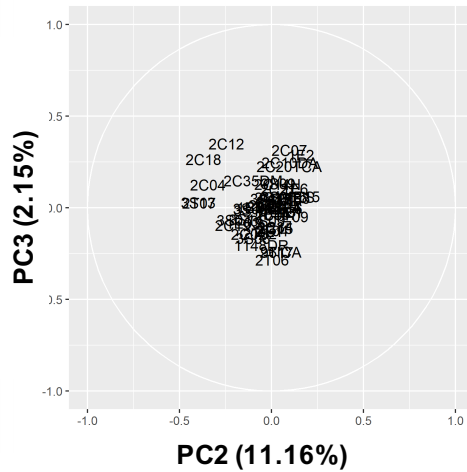

F

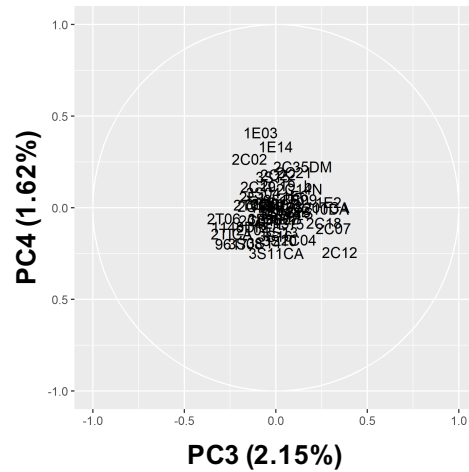

Supplement: Supplementary file 2 — Supplementary Information 2. [file 41598_2022_23544_MOESM2_ESM.pdf]

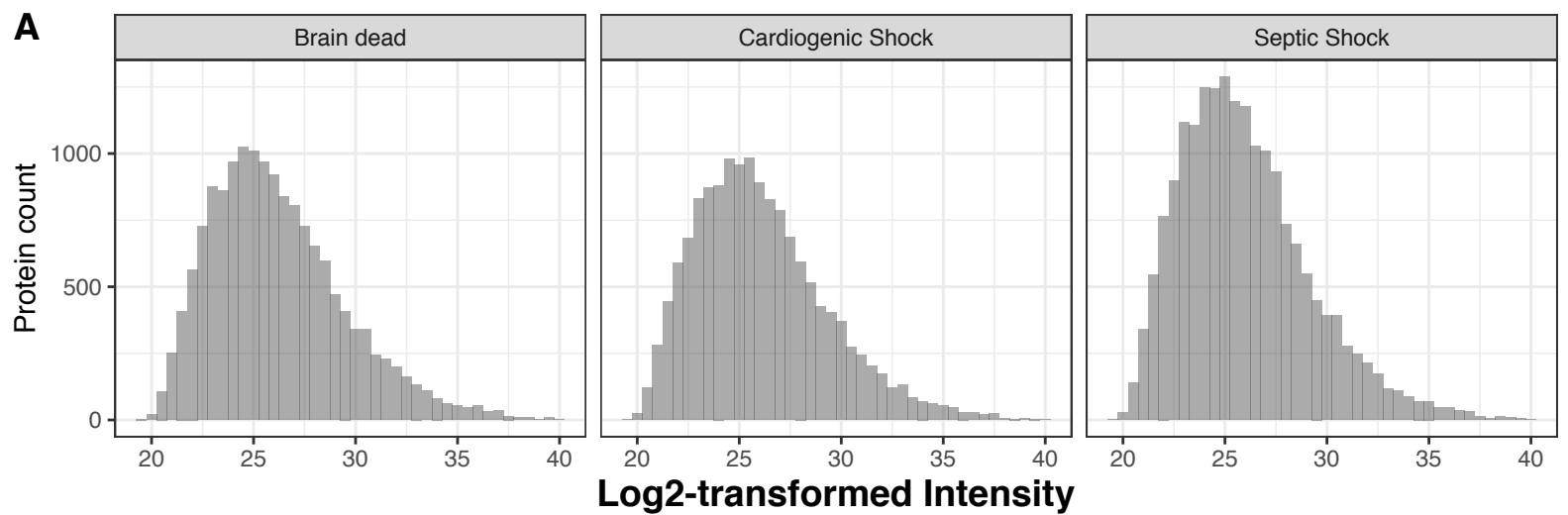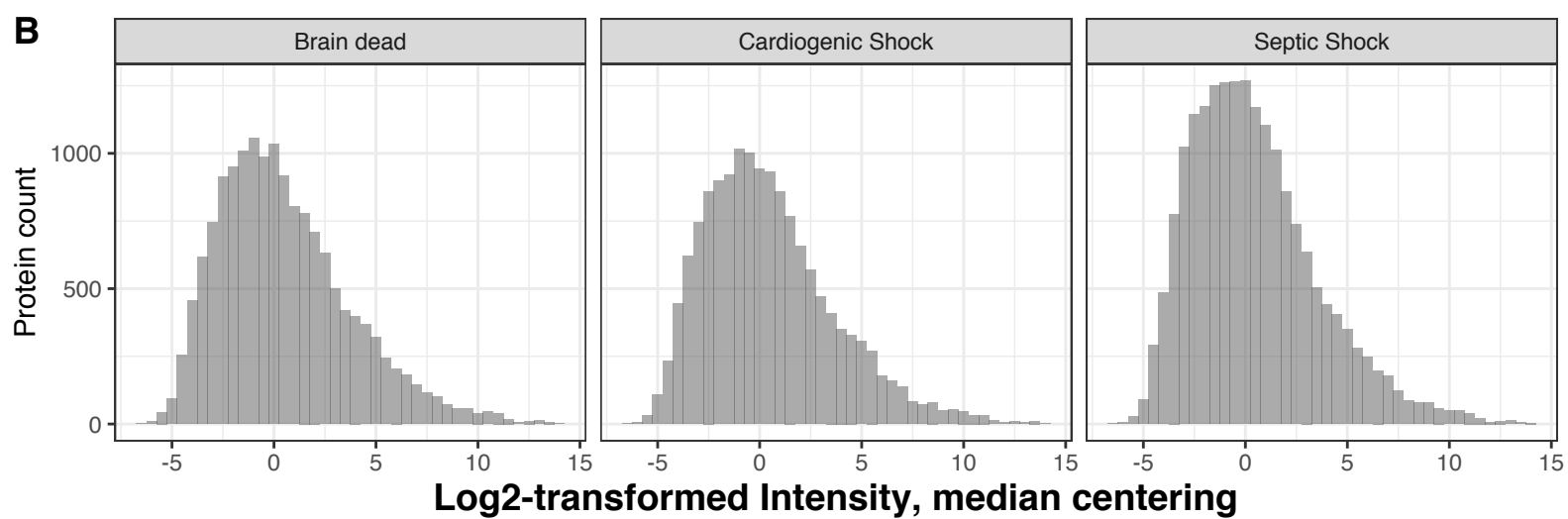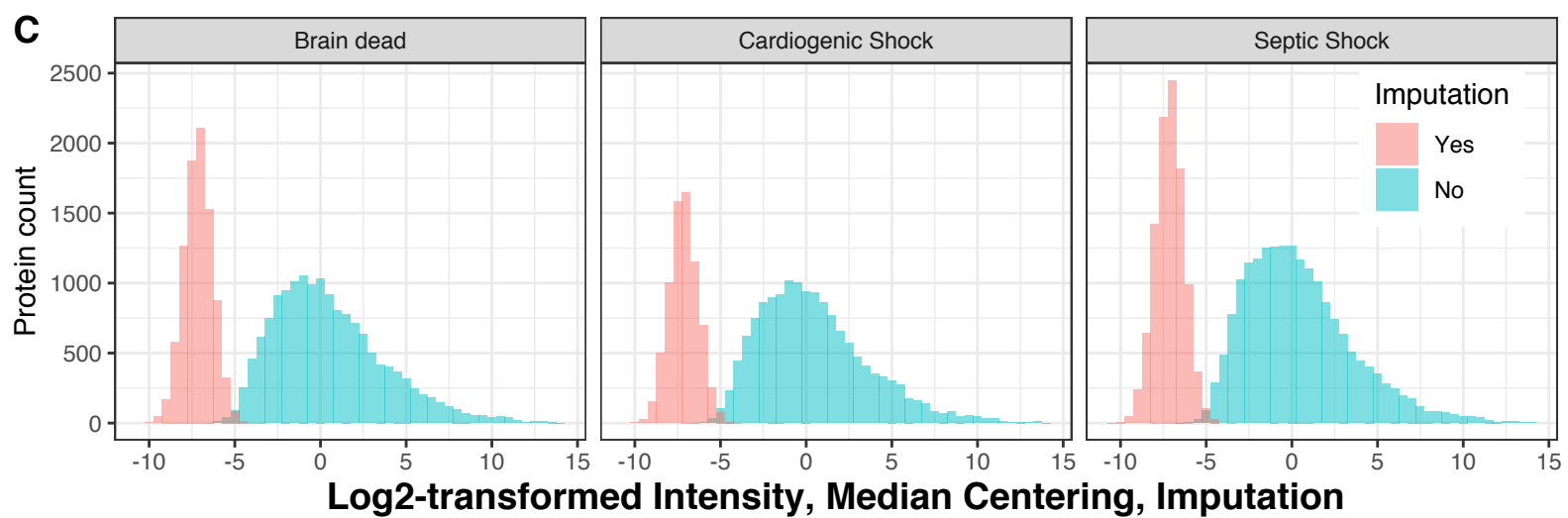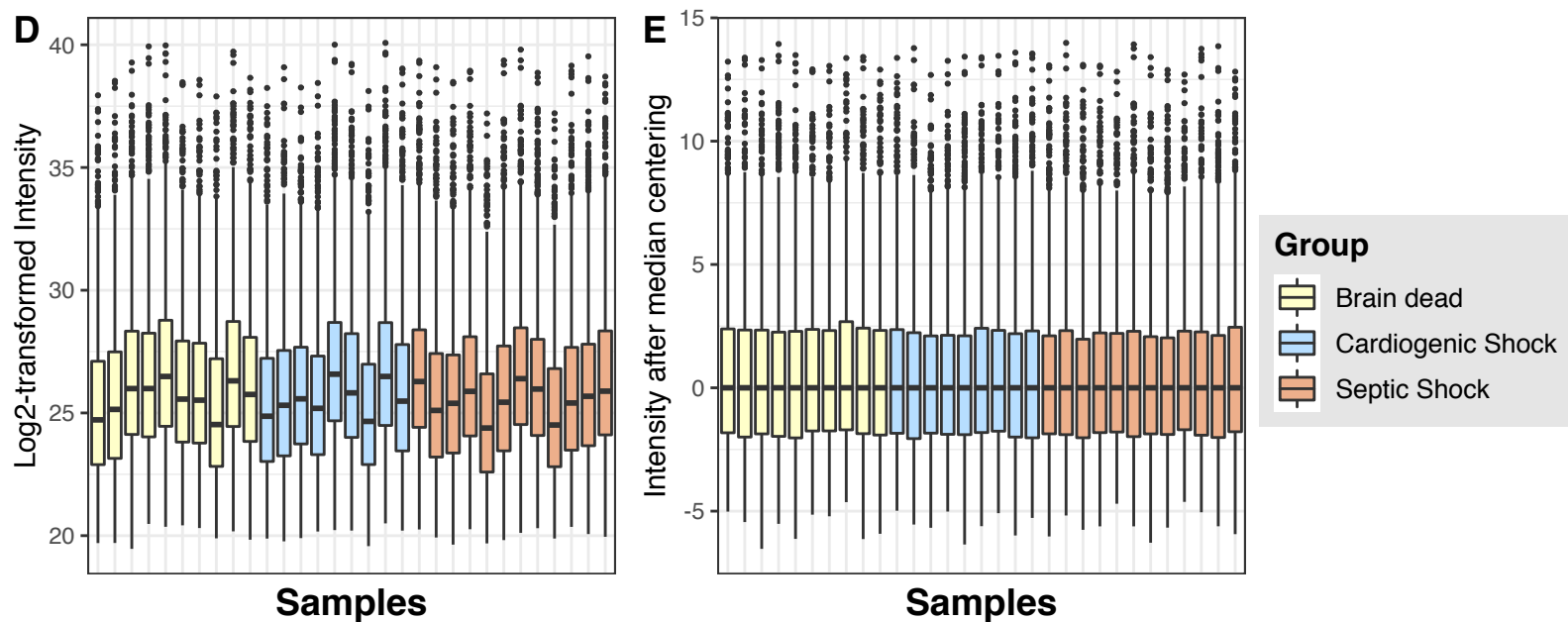

Supplement: Supplementary file 3 — Supplementary Information 3. [file 41598_2022_23544_MOESM3_ESM.pdf]

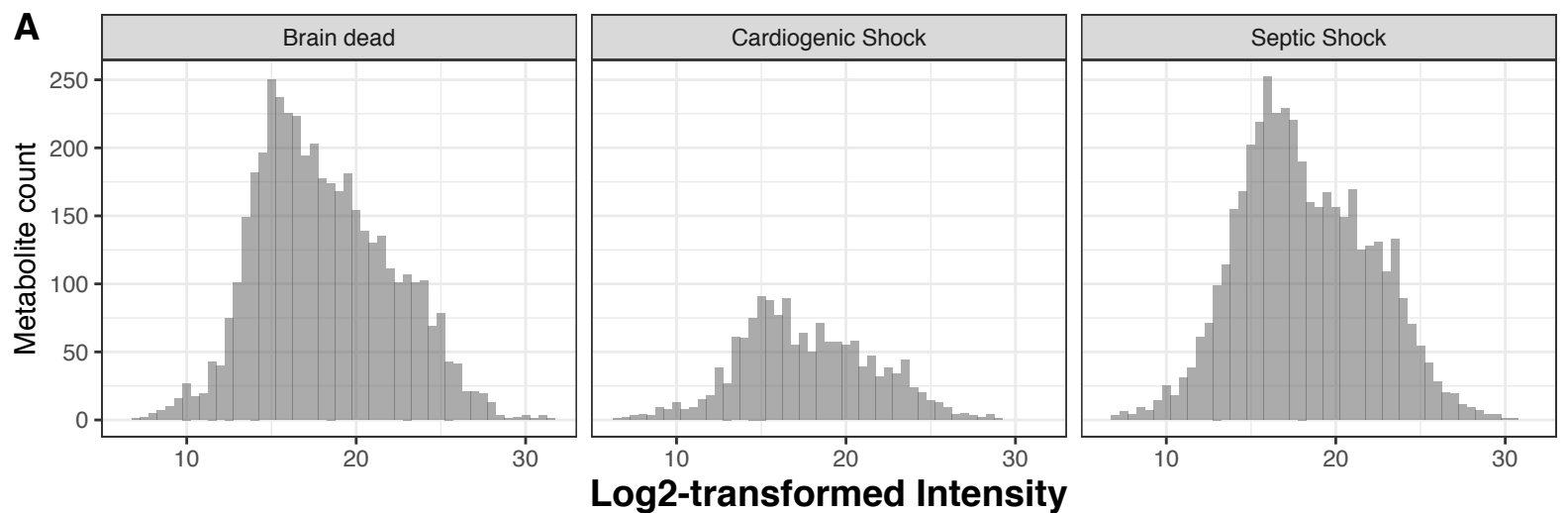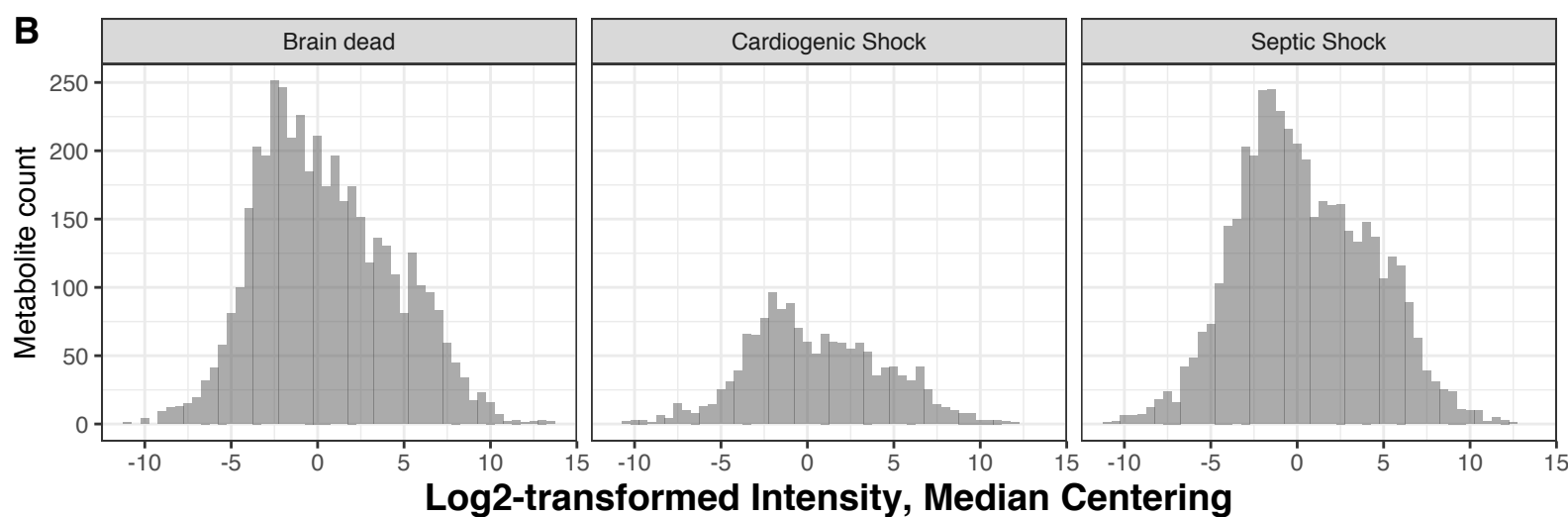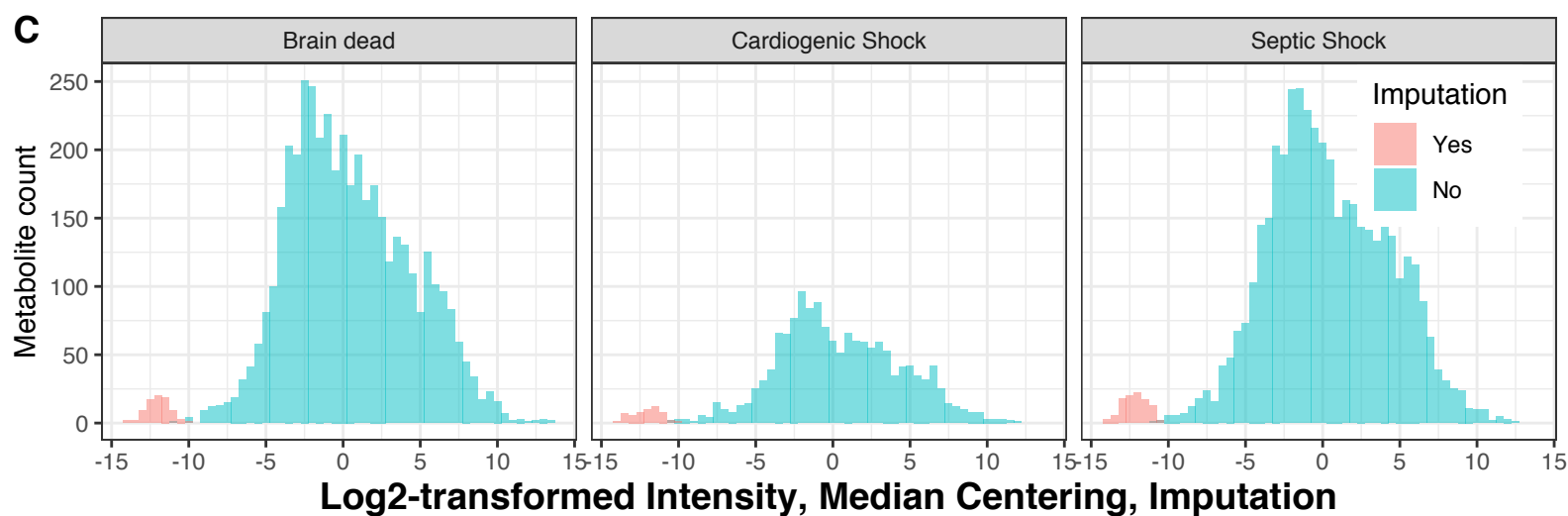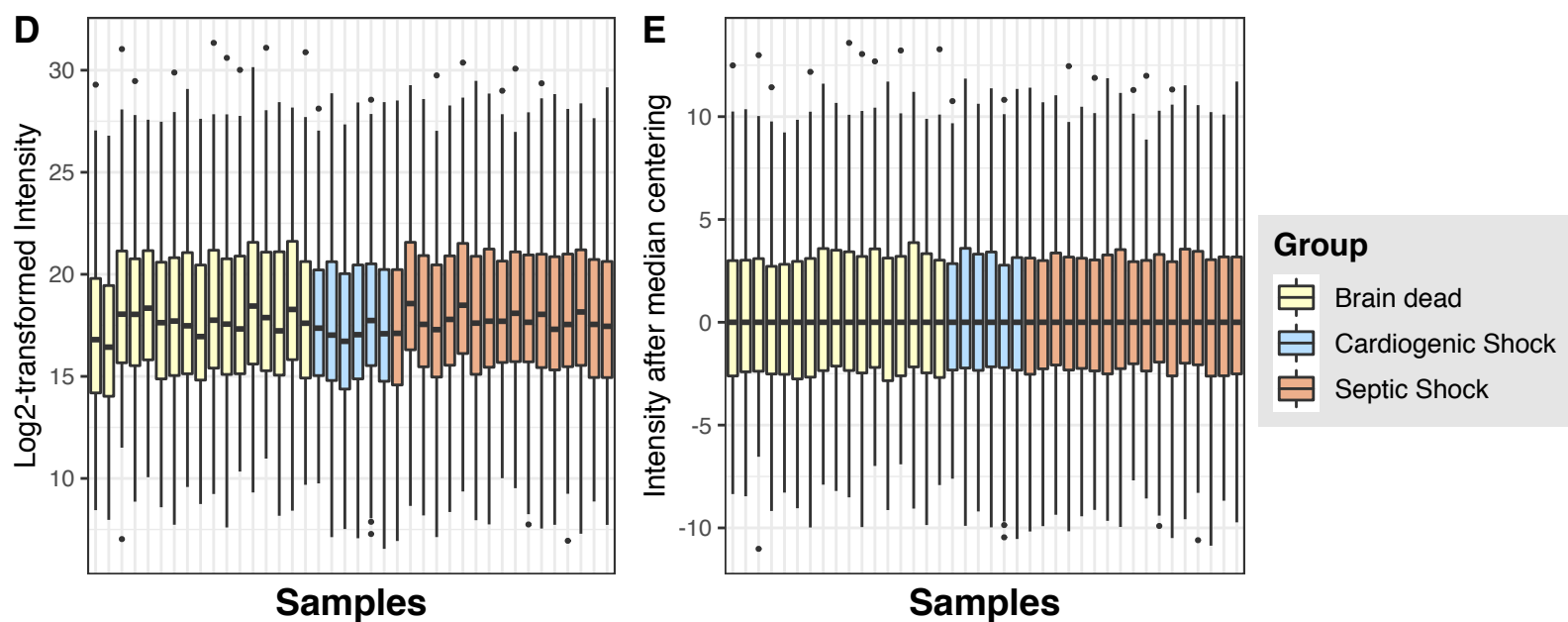

Supplement: Supplementary file 4 — Supplementary Information 4. [file 41598_2022_23544_MOESM4_ESM.pdf]

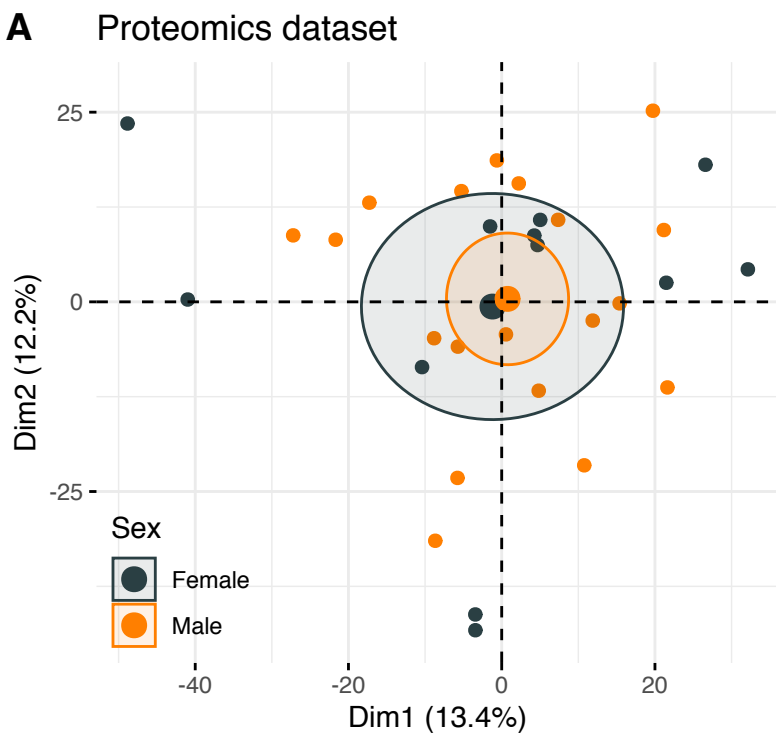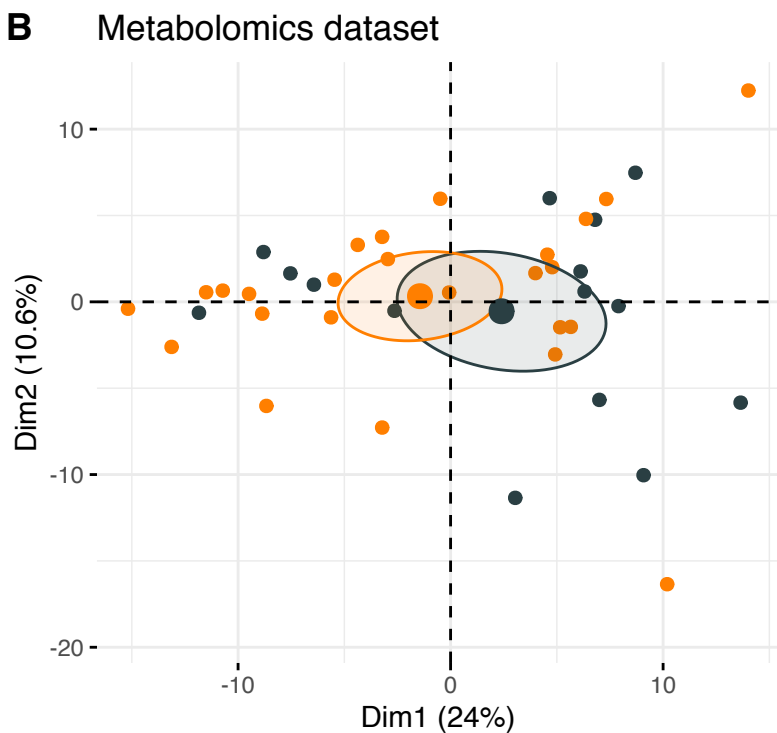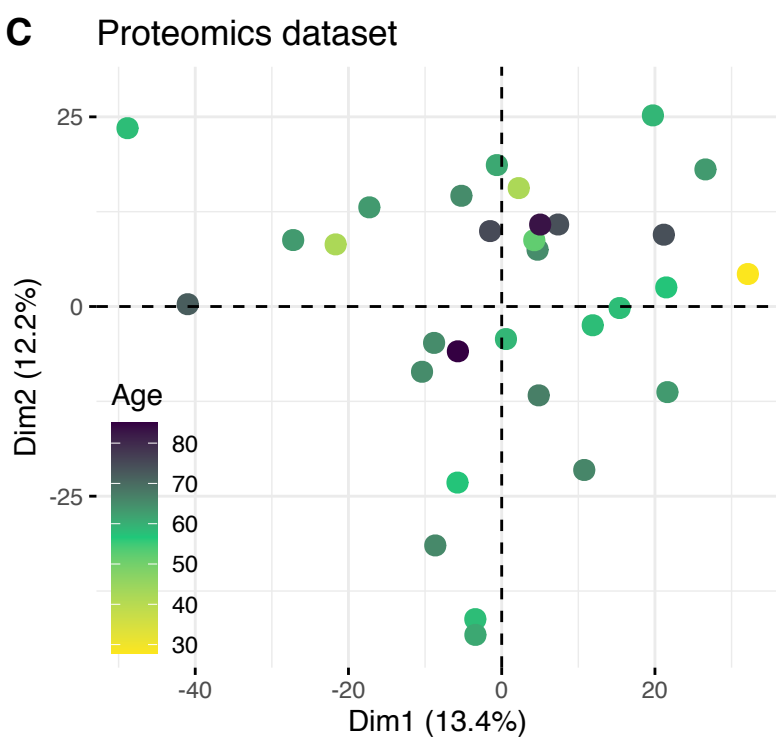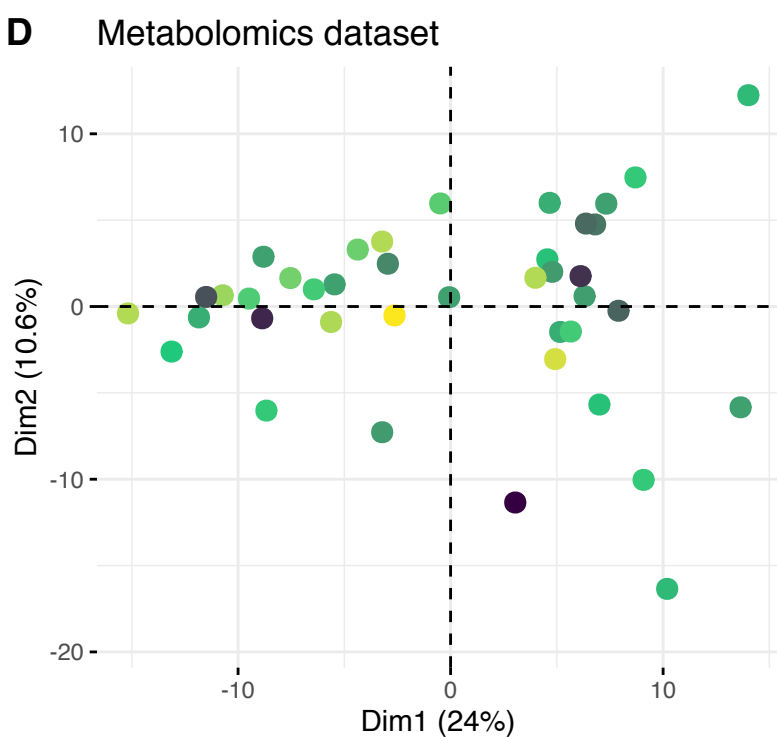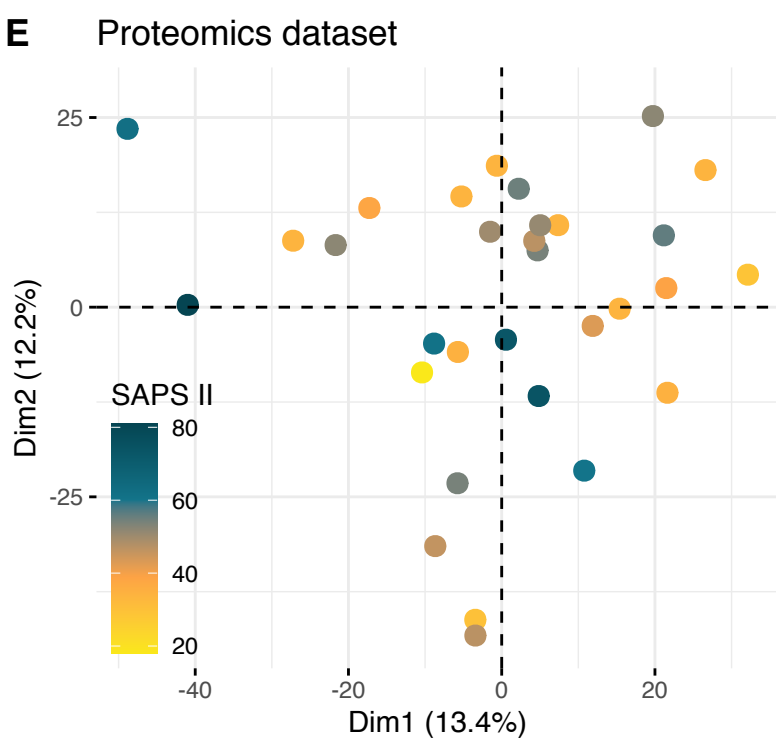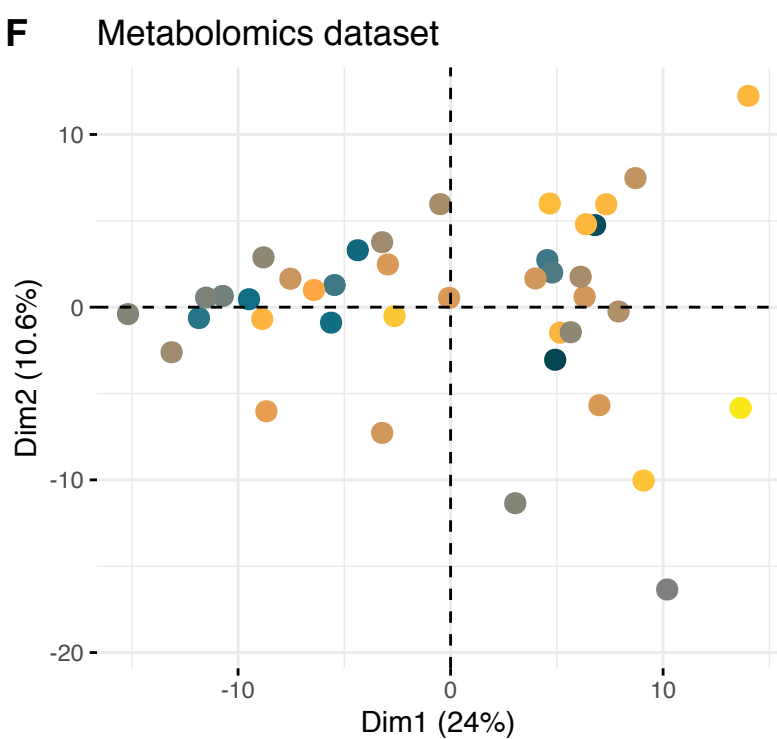

Supplement: Supplementary file 8 — Supplementary Information 8. [file 41598_2022_23544_MOESM8_ESM.pdf]

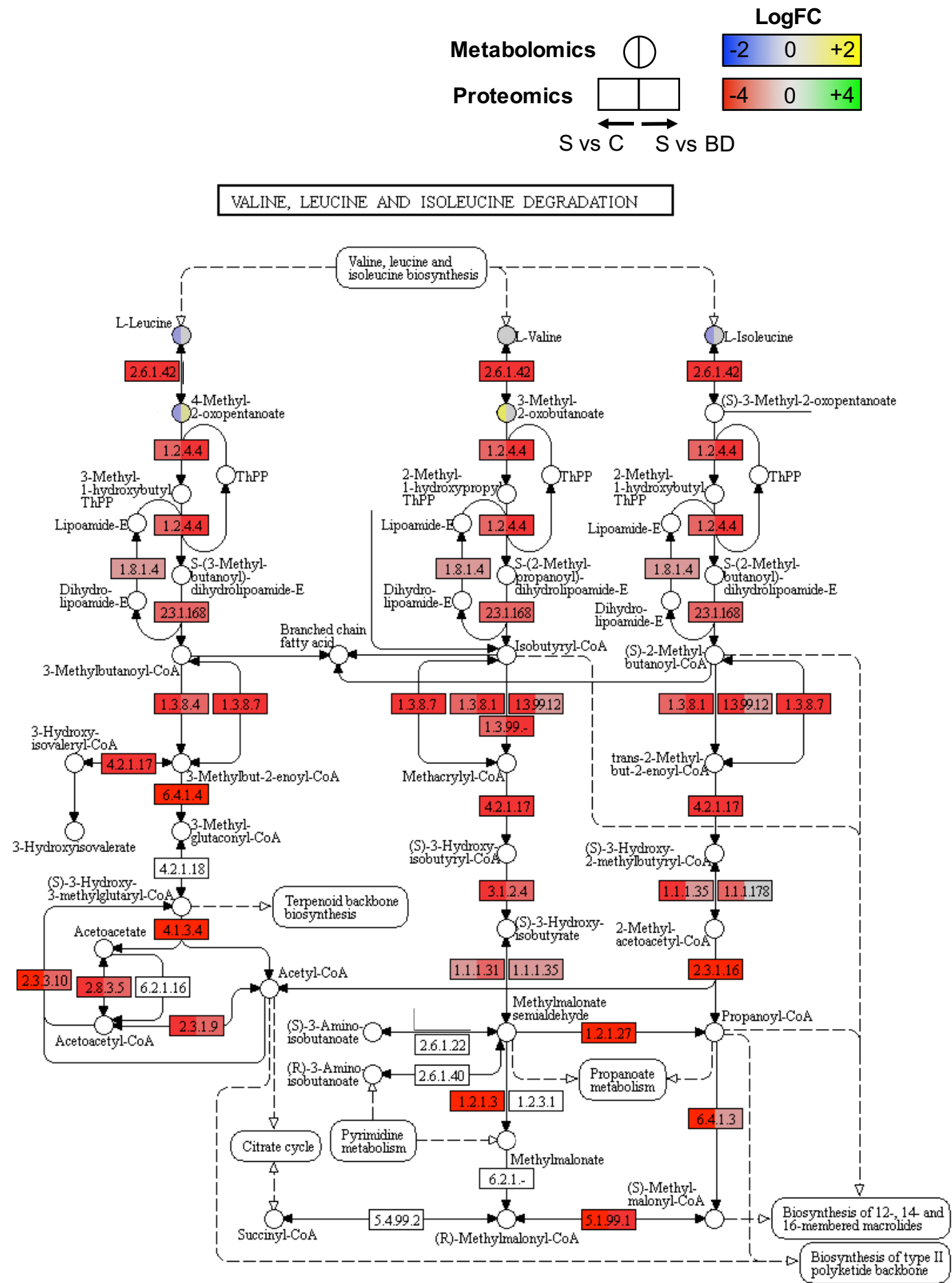

Supplement: Supplementary file 9 — Supplementary Information 9. [file 41598_2022_23544_MOESM9_ESM.pdf]

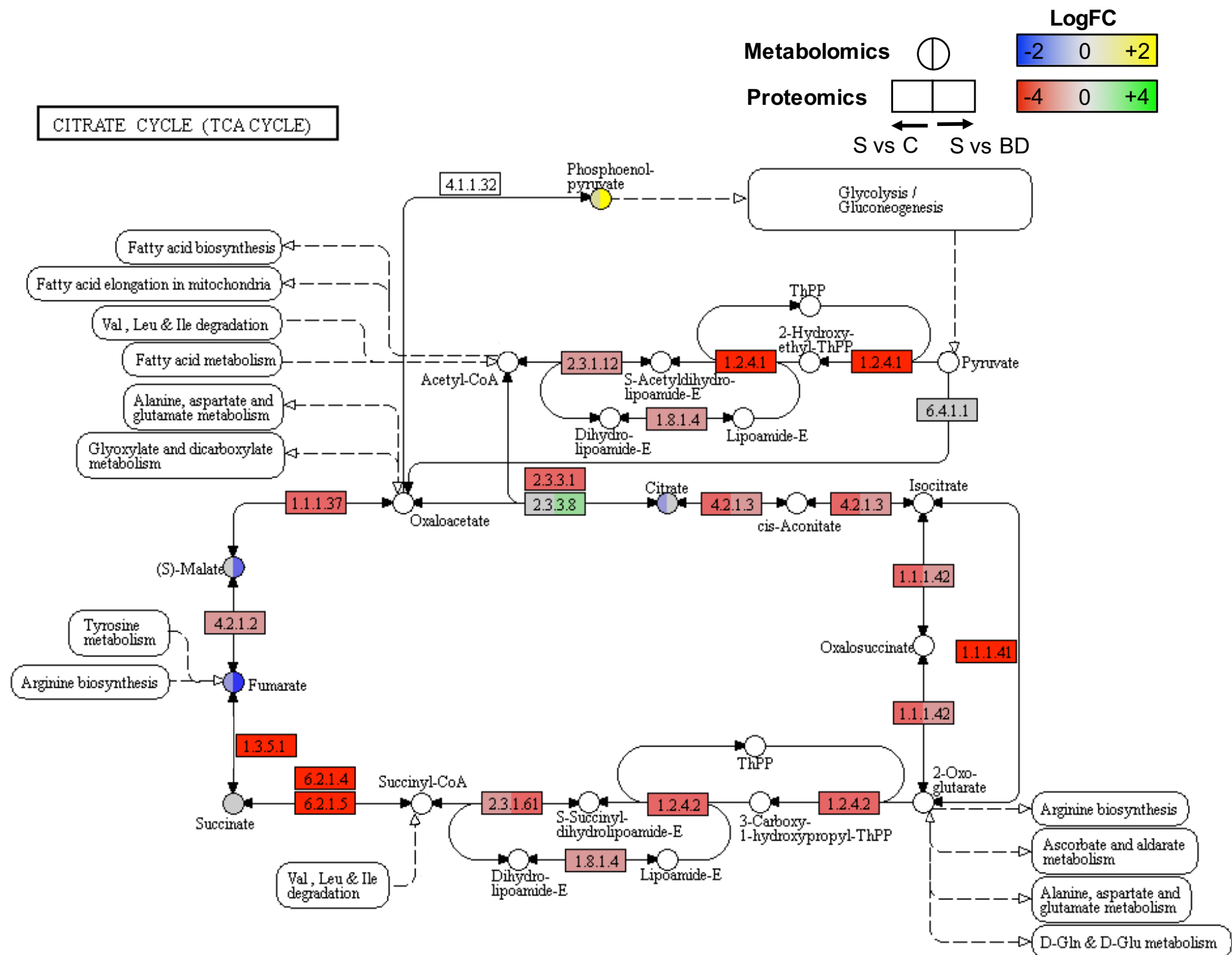

Supplement: Supplementary file 10 — Supplementary Information 10. [file 41598_2022_23544_MOESM10_ESM.pdf]

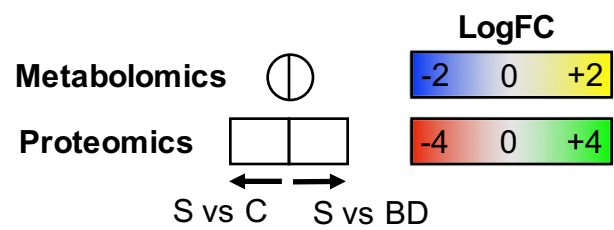

# SYNTHESIS AND DEGRADATION OF KETONE BODIES

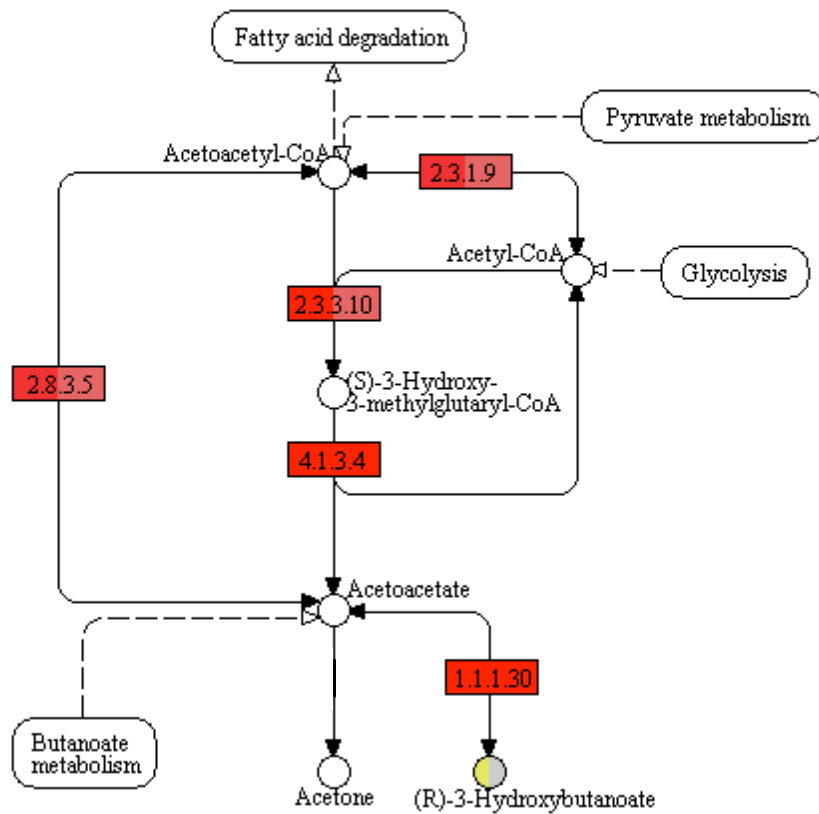

Data on KEGG graph  
Rendered by Pathview

Supplement: Supplementary file 11 — Supplementary Information 11. [file 41598_2022_23544_MOESM11_ESM.pdf]

## FATTY ACID DEGRADATION

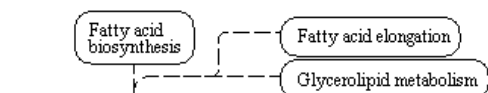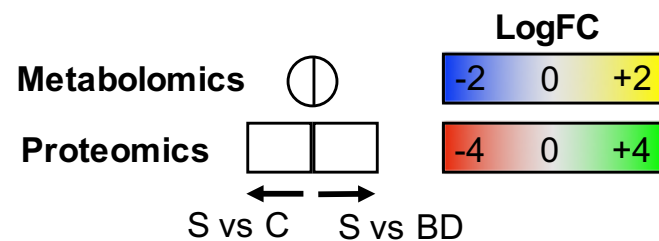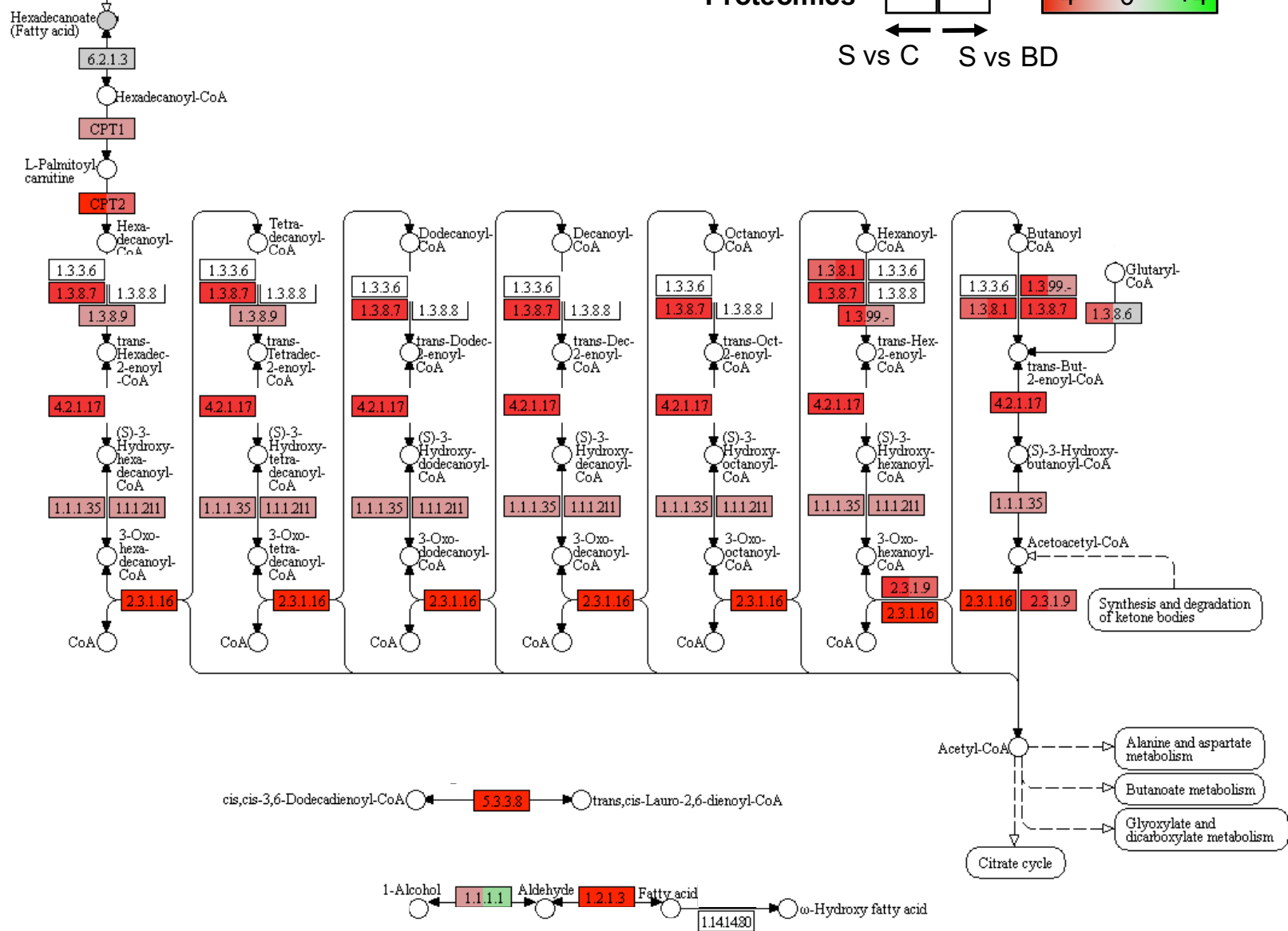

Supplement: Supplementary file 12 — Supplementary Information 12. [file 41598_2022_23544_MOESM12_ESM.pdf]

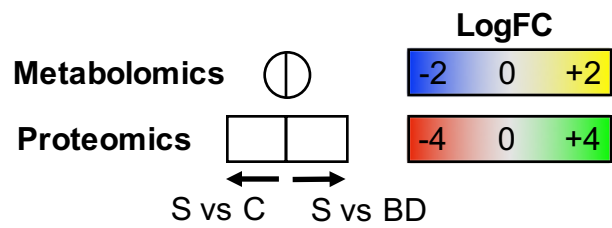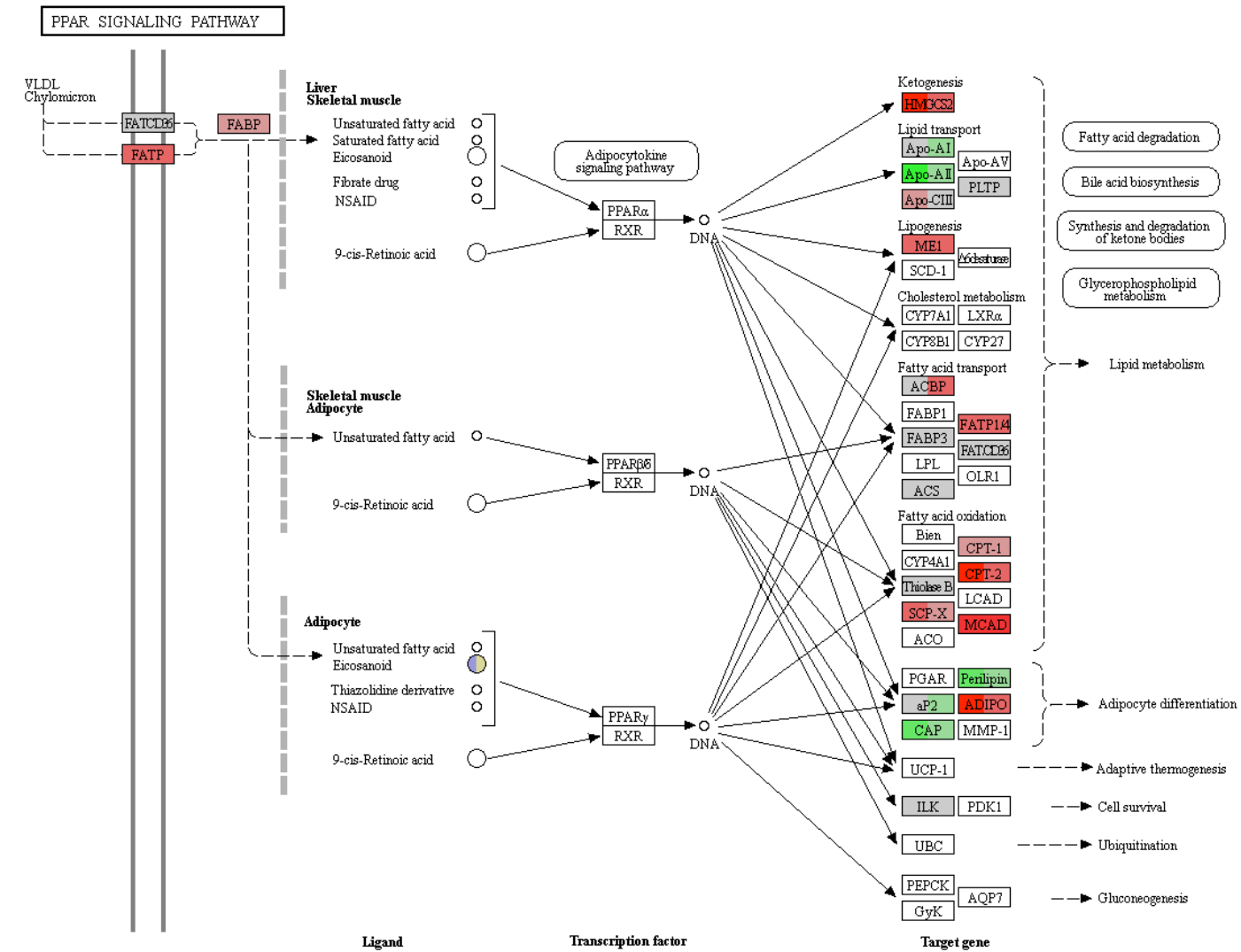

Data on KEGG graph  
Rendered by Pathview

Supplement: Supplementary file 13 — Supplementary Information 13. [file 41598_2022_23544_MOESM13_ESM.pdf]

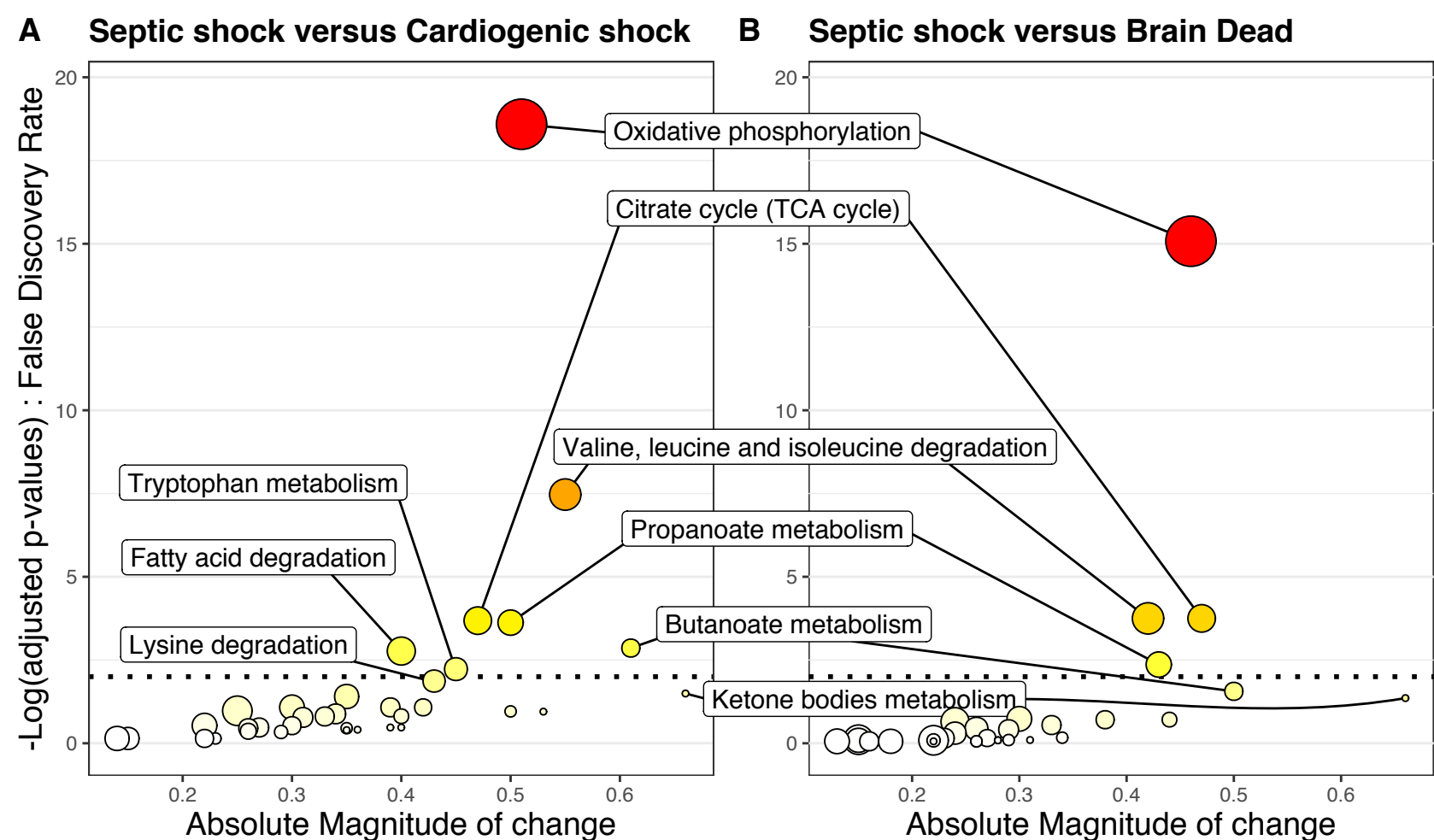

Supplement: Supplementary file 14 — Supplementary Information 14. [file 41598_2022_23544_MOESM14_ESM.pdf]

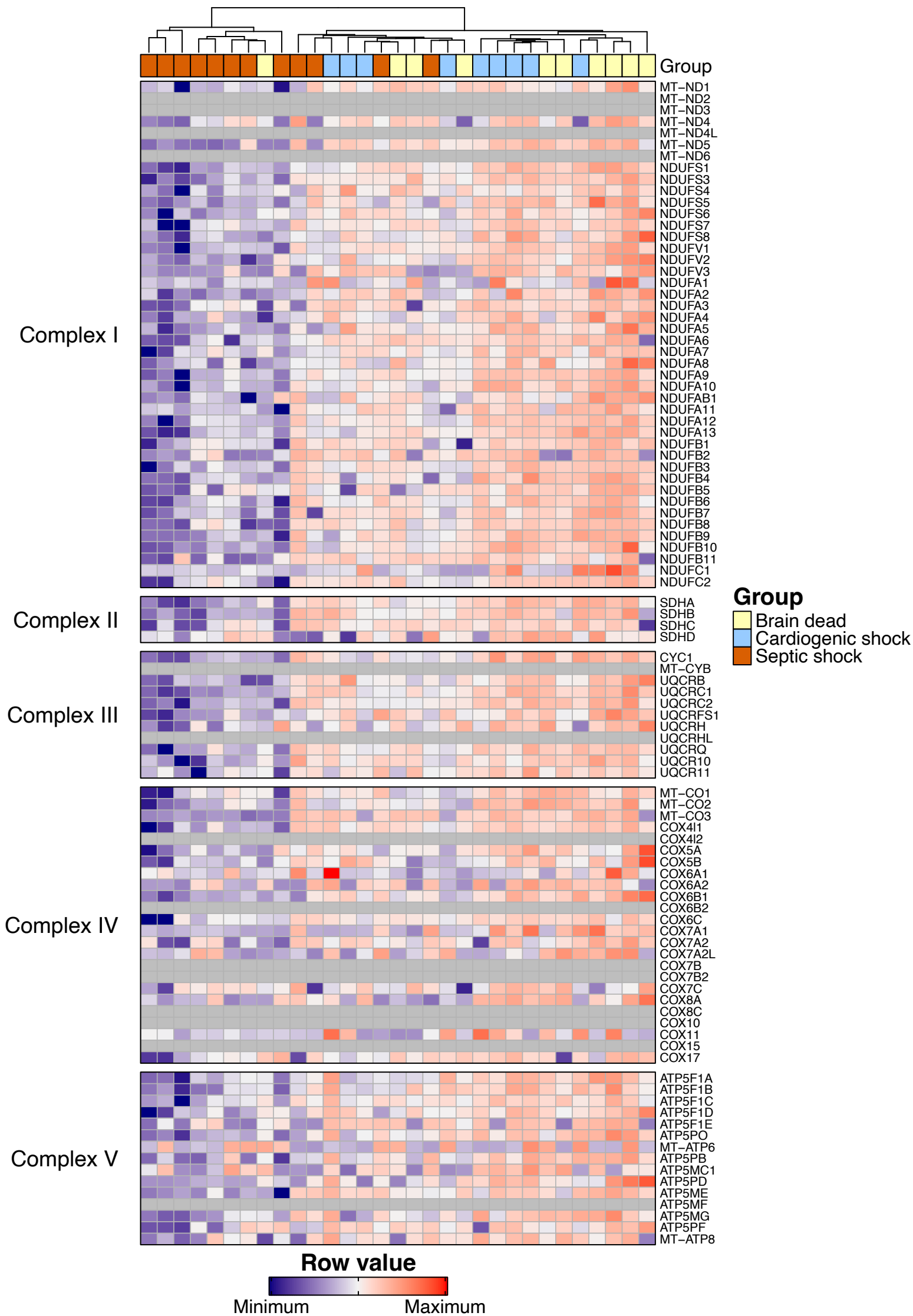

Supplement: Supplementary file 15 — Supplementary Information 15. [file 41598_2022_23544_MOESM15_ESM.pdf]
