# Supplementary material for: Using a multiomics approach to unravel a septic shock specific signature in skeletal muscle
Source: Sci Rep. 2022 Nov 5;12:18776. doi: 10.1038/s41598-022-23544-8 (PMC9637214; doi:10.1038/s41598-022-23544-8)
Supplement: Supplementary file 5 — Supplementary Information 5. [file 41598_2022_23544_MOESM5_ESM.pdf]

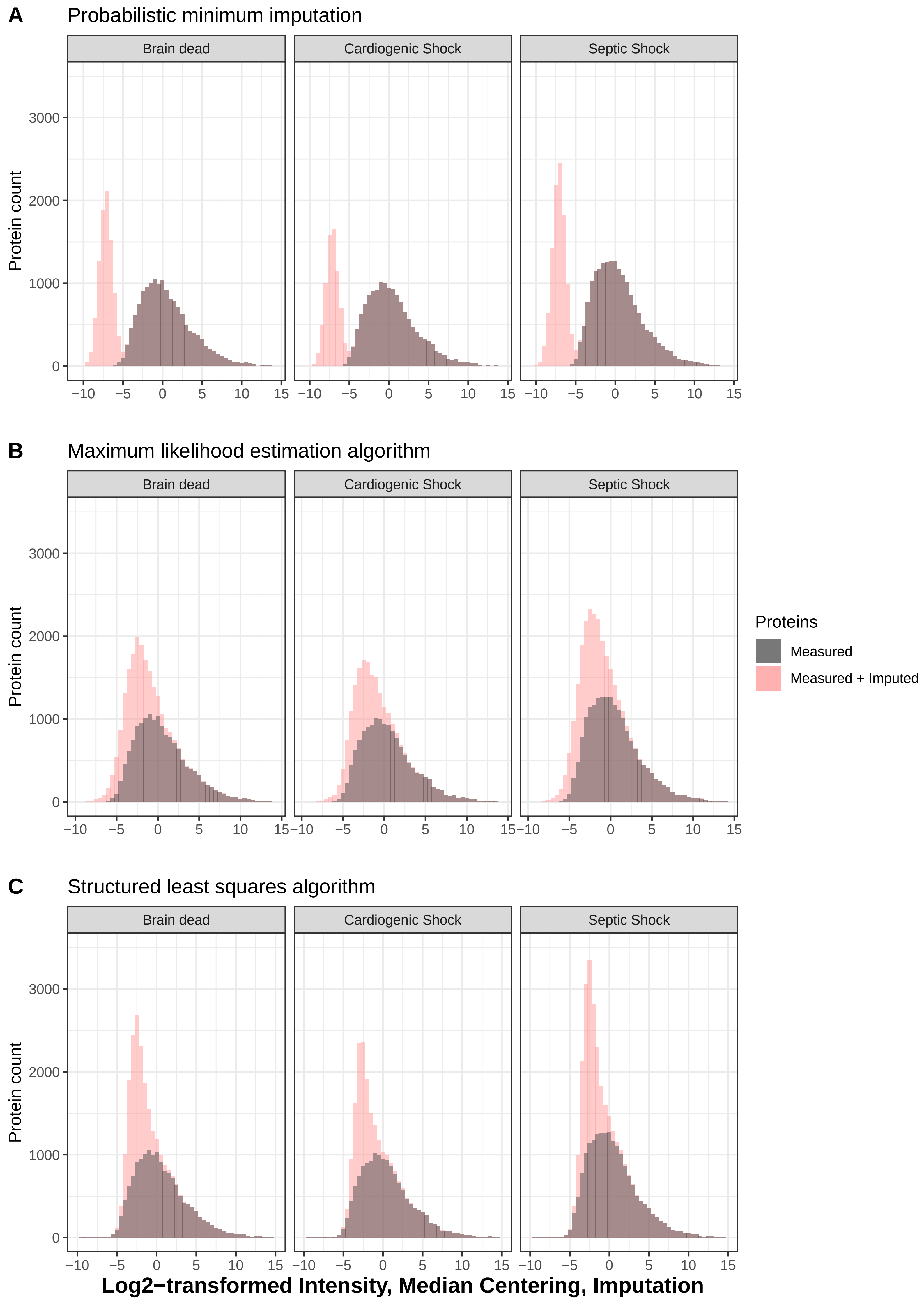
A
Probabilistic minimum imputation
Brain dead
Cardiogenic Shock
Septic Shock
Protein count
0
1000
2000
3000
−10
−5
0
5
10
15
B
Maximum likelihood estimation algorithm
Brain dead
Cardiogenic Shock
Septic Shock
Protein count
Proteins
Measured
Measured + Imputed
C
Structured least squares algorithm
Brain dead
Cardiogenic Shock
Septic Shock
Protein count
Log2−transformed Intensity, Median Centering, Imputation
