# Supplementary material for: Using a multiomics approach to unravel a septic shock specific signature in skeletal muscle
Source: Sci Rep. 2022 Nov 5;12:18776. doi: 10.1038/s41598-022-23544-8 (PMC9637214; doi:10.1038/s41598-022-23544-8)
Supplement: Supplementary file 6 — Supplementary Information 6. [file 41598_2022_23544_MOESM6_ESM.pdf]

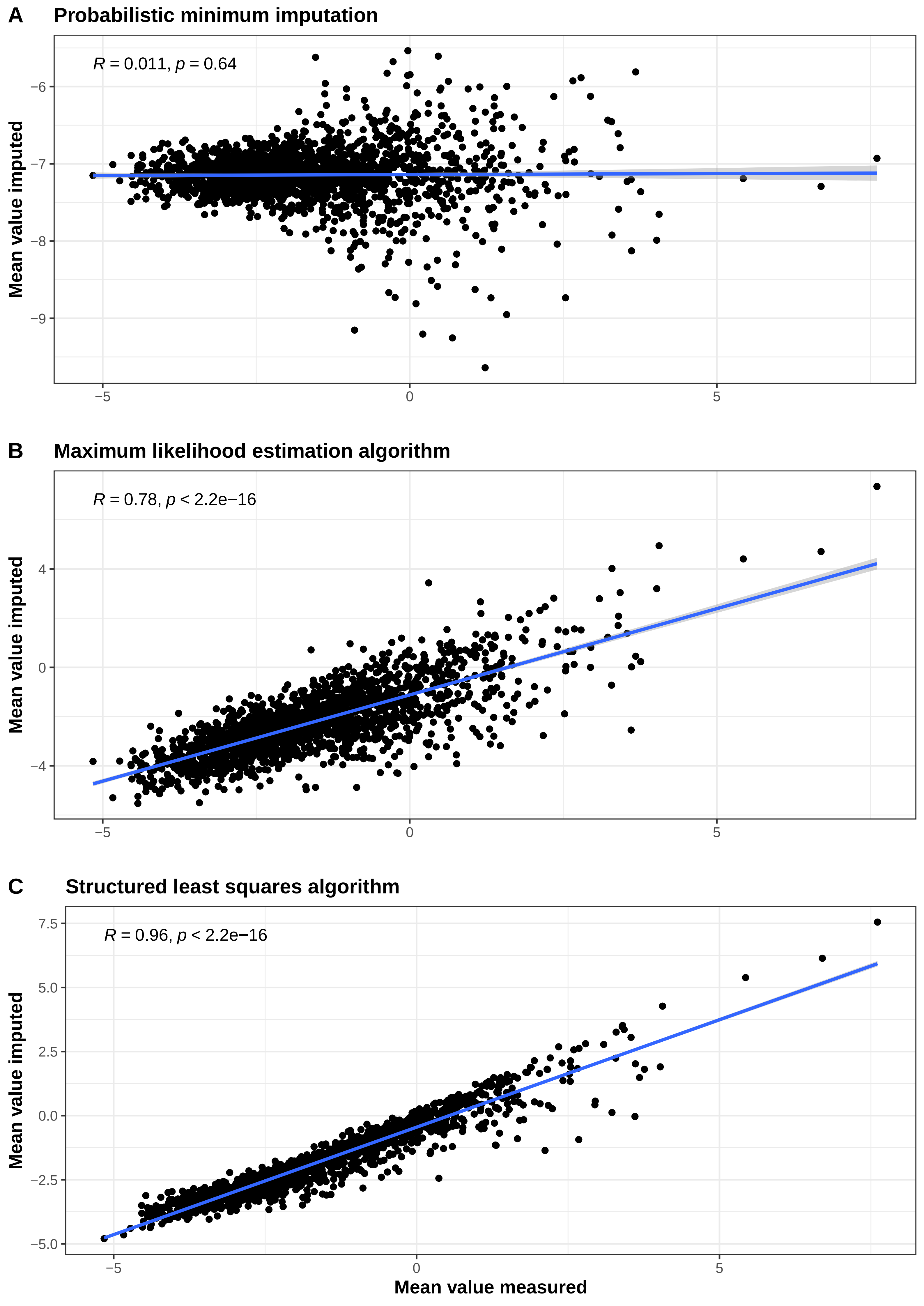

A
Probabilistic minimum imputation
R = 0.011, p = 0.64
Mean value imputed
−6
−7
−8
−9
−5
0
5
B
Maximum likelihood estimation algorithm
R = 0.78, p < 2.2e−16
Mean value imputed
4
0
−4
−5
0
5
C
Structured least squares algorithm
R = 0.96, p < 2.2e−16
Mean value imputed
7.5
5.0
2.5
0.0
−2.5
−5.0
−5
0
5
Mean value measured
