# Supplementary material for: Using a multiomics approach to unravel a septic shock specific signature in skeletal muscle
Source: Sci Rep. 2022 Nov 5;12:18776. doi: 10.1038/s41598-022-23544-8 (PMC9637214; doi:10.1038/s41598-022-23544-8)
Supplement: Supplementary file 7 — Supplementary Information 7. [file 41598_2022_23544_MOESM7_ESM.pdf]

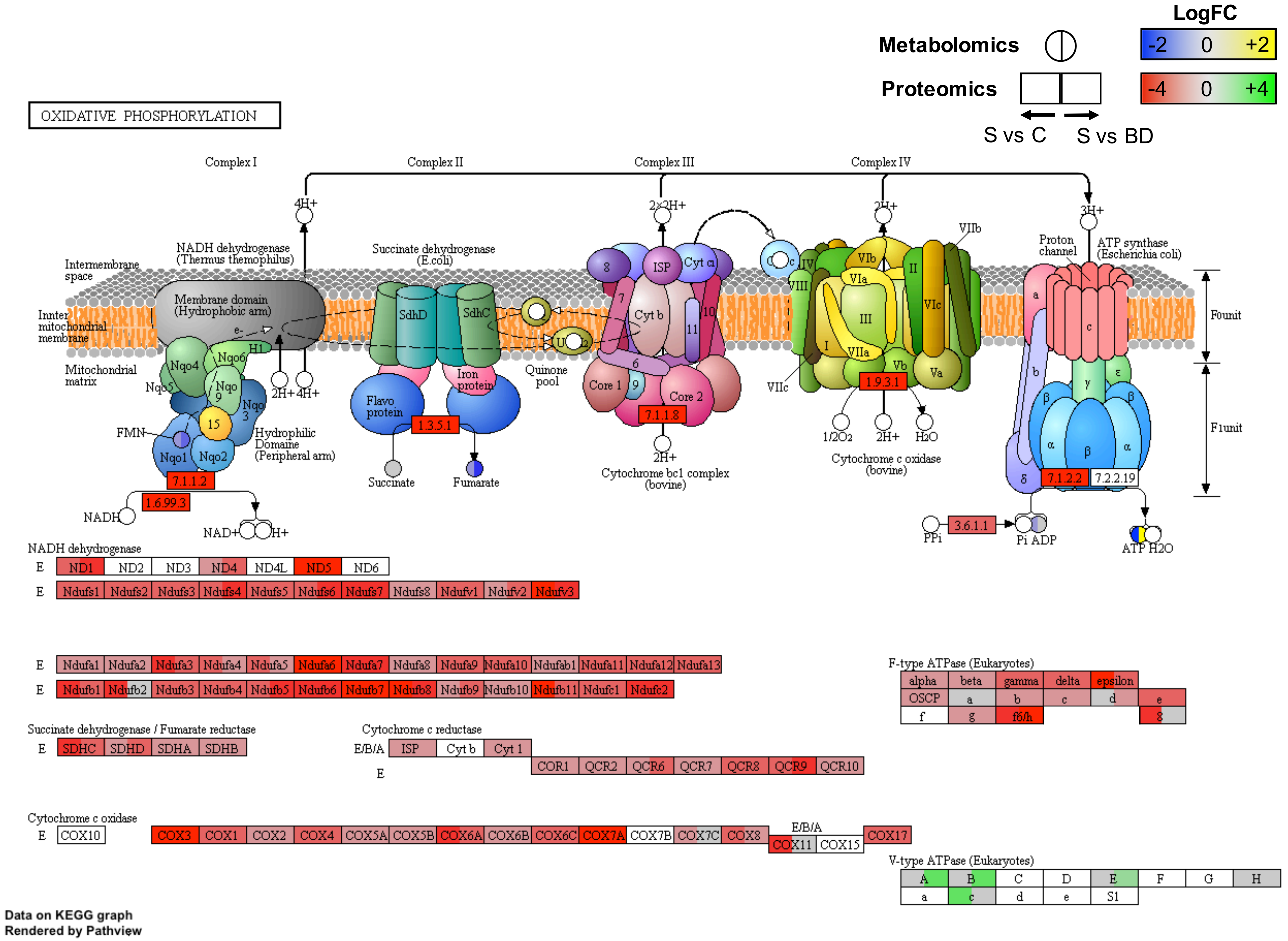
LogFC
Metabolomics
-2 0 +2
Proteomics
-4 0 +4
S vs C
S vs BD
OXIDATIVE PHOSPHORYLATION
Complex I
Complex II
Complex III
Complex IV
4H+
2×2H+
2H+
3H+
NADH dehydrogenase (Thermus themophilus)
Succinate dehydrogenase (E.coli)
Intermembrane space
Inner mitochondrial membrane
Mitochondrial matrix
Membrane domain (Hydrophobic arm)
e-
H1
Nqo4
Nqo6
Nqo5
Nqo 9
Nqo 3
15
FMN
Nqo1
Nqo2
Hydrophilic Domaine (Peripheral arm)
2H+ 4H+
7.1.1.2
1.6.99.3
NADH
NAD+
H+
SdhD
SdhC
Flavo protein
Iron protein
1.3.5.1
Succinate
Fumarate
Quinone pool
8
ISP
Cyt a
7
Cyt b
10
11
6
Core 1
9
Core 2
7.1.1.8
2H+
Cytochrome bc1 complex (bovine)
VIIb
IV
VIII
VIb
VIa
II
VIc
III
I
VIIa
Vb
Va
VIIc
1.9.3.1
1/2O2
2H+
H2O
Cytochrome c oxidase (bovine)
Proton channel
ATP synthase (Escherichia coli)
a
c
b
ε
γ
β
α
δ
7.1.2.2
7.2.2.19
Founit
F1unit
PPi
3.6.1.1
Pi ADP
ATP H2O
NADH dehydrogenase
E ND1 ND2 ND3 ND4 ND4L ND5 ND6
E Ndufs1 Ndufs2 Ndufs3 Ndufs4 Ndufs5 Ndufs6 Ndufs7 Ndufs8 Ndufv1 Ndufv2 Ndufv3
E Ndufa1 Ndufa2 Ndufa3 Ndufa4 Ndufa5 Ndufa6 Ndufa7 Ndufa8 Ndufa9 Ndufa10 Ndufab1 Ndufa11 Ndufa12 Ndufa13
E Ndufb1 Ndufb2 Ndufb3 Ndufb4 Ndufb5 Ndufb6 Ndufb7 Ndufb8 Ndufb9 Ndufb10 Ndufb11 Ndufc1 Ndufc2
F-type ATPase (Eukaryotes)
alpha beta gamma delta epsilon
OSCP a b c d e
f g f6/h 8
Succinate dehydrogenase / Fumarate reductase
E SDHC SDHD SDHA SDHB
Cytochrome c reductase
E/B/A ISP Cyt b Cyt 1
E COR1 QCR2 QCR6 QCR7 QCR8 QCR9 QCR10
Cytochrome c oxidase
E COX10
COX3 COX1 COX2 COX4 COX5A COX5B COX6A COX6B COX6C COX7A COX7B COX7C COX8
E/B/A
COX11 COX15
COX17
V-type ATPase (Eukaryotes)
A B C D E F G H
a c d e S1
Data on KEGG graph
Rendered by Pathview
